# Comparative genomics reveals cotton‐specific virulence factors in flexible genomic regions in *Verticillium dahliae* and evidence of horizontal gene transfer from *Fusarium*

**DOI:** 10.1111/nph.14861

**Published:** 2017-10-30

**Authors:** Jie‐Yin Chen, Chun Liu, Yue‐Jing Gui, Kai‐Wei Si, Dan‐Dan Zhang, Jie Wang, Dylan P. G. Short, Jin‐Qun Huang, Nan‐Yang Li, Yong Liang, Wen‐Qi Zhang, Lin Yang, Xue‐Feng Ma, Ting‐Gang Li, Lei Zhou, Bao‐Li Wang, Yu‐Ming Bao, Krishna V. Subbarao, Geng‐Yun Zhang, Xiao‐Feng Dai

**Affiliations:** ^1^ Laboratory of Cotton Disease Institute of Food Science and Technology Chinese Academy of Agricultural Sciences Beijing 100193 China; ^2^ BGI‐Shenzhen Shenzhen Guangdong 518083 China; ^3^ Department of Plant Pathology University of California Davis CA 95616 USA

**Keywords:** comparative genomics, dominant adaptation, horizontal gene transfer, lineage‐specific genes, *Verticillium dahliae*

## Abstract

- *Verticillium dahliae* isolates are most virulent on the host from which they were originally isolated. Mechanisms underlying these dominant host adaptations are currently unknown. We sequenced the genome of *V. dahliae* Vd991, which is highly virulent on its original host, cotton, and performed comparisons with the reference genomes of JR2 (from tomato) and VdLs.17 (from lettuce).
- Pathogenicity‐related factor prediction, orthology and multigene family classification, transcriptome analyses, phylogenetic analyses, and pathogenicity experiments were performed.
- The Vd991 genome harbored several exclusive, lineage‐specific (LS) genes within LS regions (LSRs). Deletion mutants of the seven genes within one LSR (G‐LSR2) in Vd991 were less virulent only on cotton. Integration of G‐LSR2 genes individually into JR2 and VdLs.17 resulted in significantly enhanced virulence on cotton but did not affect virulence on tomato or lettuce. Transcription levels of the seven LS genes in Vd991 were higher during the early stages of cotton infection, as compared with other hosts. Phylogenetic analyses suggested that G‐LSR2 was acquired from *Fusarium oxysporum* f. sp. *vasinfectum* through horizontal gene transfer.
- Our results provide evidence that horizontal gene transfer from *Fusarium* to Vd991 contributed significantly to its adaptation to cotton and may represent a significant mechanism in the evolution of an asexual plant pathogen.

## Introduction

*Verticillium dahliae* is an asexually reproducing, soilborne, vascular wilt‐causing phytopathogenic fungus that affects nearly 200 plant species. *V. dahliae* commonly causes serious economic losses worldwide in high‐value agricultural crops, including cotton, lettuce, olive, potato, strawberry and others (Pegg & Brady, 2002; Fradin & Thomma, 2006; Inderbitzin & Subbarao, 2014). The survival structures produced by the pathogen are microsclerotia that remain viable in the soil in the absence of a host for > 14 yr (Wilhelm, 1995). This characteristic plus its broad host range make *V. dahliae* a difficult pathogen to manage once it is established in soil.

The fungus *V. dahliae* has a highly clonal population structure (Fradin & Thomma, 2006; Milgroom *et al*., 2014). Large‐scale population genetic analyses revealed that *V. dahliae* has differentiated into several genetic clusters (clonal lineages) (Milgroom *et al*., 2014; Short *et al*., 2015). Genomic studies have provided evidence that chromosomal rearrangements and lineage‐specific (LS) genomic regions contribute significantly to virulence and niche adaptation (de Jonge *et al*., 2013) and that transposable elements (TEs) are the major generators of chromosomal rearrangements that facilitate the rapid development of novel genes involved in host adaptation (Klosterman *et al*., 2011; Amyotte *et al*., 2012; de Jonge *et al*., 2013). Furthermore, investigations into the molecular basis of *V. dahliae* pathogenicity over the past 10 yr have revealed that a diversity of genes are involved in the manipulation of host immunity, carbon and nitrogen metabolism, and signaling regulation, which are also related to virulence and niche adaptation (Klimes *et al*., 2015).

While *V. dahliae* causes vascular wilt in a broad range of dicotyledonous host plants (Pegg & Brady, 2002), the severity of symptoms on various host plants can vary considerably between individual strains, and is generally more severe on the host of origin than on other hosts (Tjamos, 1981; Bhat & Subbarao, 1999). This implies that host‐specific adaptations have arisen during the evolution of *V. dahliae*. For example, there is extensive genomic variation between *V. dahliae* isolates JR2 and VdLs.17, which cause serious Verticillium wilt on tomato and lettuce, respectively (Klosterman *et al*., 2011; de Jonge *et al*., 2013). Currently, the genetic bases of dominant adaptations are unknown.

To date, most investigations into the nature of host‐specific adaptations have focused on differences between species of plant pathogens, while fewer studies have been conducted to investigate and explain the intraspecific diversity of host‐specific adaptations. The *V. dahliae* isolate Vd991 (nonrace 1, defoliating), collected from *Gossypium hirsutum* (Chen *et al*., 2016), is highly virulent on its original host but is only moderately virulent on tomato and even less virulent on lettuce (Supporting Information Fig. S1). In this study, we sequenced the genome of strain Vd991 by combining PacBio RS II and Illumina sequencing technologies with the following objectives: to identify chromosomal rearrangements and predict genes in Vd991; to assign functional annotations and compare gene content between the three *V. dahliae* genomes and identify strain‐specific LS regions (LSRs) and LS genes in Vd991; to perform transcriptome analysis of Vd991 during infection of cotton, lettuce, and tomato roots; to investigate the functions of genes exclusive to Vd991 LSRs via targeted deletion experiments and transformation experiments using other *V. dahliae* strains; and to perform phylogenetic analyses on genes within the LSRs of Vd991 using genes from *Verticillium* and other fungi.

## Materials and Methods

### Sample preparation and sequencing

DNA from *Verticillium dahliae* strain Vd991 (an isolate from *G. hirsutum* originally collected by the Institute of Plant Protection, Jiangsu Academy of Agricultural Sciences) was extracted from a fresh mycelial suspension following 5 d of growth in potato dextrose broth at 25°C in the dark using a genomic DNA extraction kit (EZNA^®^ Fungal DNA Mini Kit, Omega, Norcross, GA, USA). Five micrograms of genomic DNA was sheared using a HydroShear (Genomic Instrumentation Services Inc., Foster City, CA, USA) with a large orifice ruby for 20 cycles at a speed code of 20. The large fragment library preparation was performed using the SMRT‐bell Template Prep Kit 1.0 (Code: 100‐259‐100; Pacific Biosciences, Menlo Park, CA, USA). The SMRT bell templates were selected using the BluePippin system (Sage Science Inc., Beverly, MA, USA) and then bound to polymerases using the DNA/Polymerase Binding Kit P5 (code: 100‐256‐000, Pacific Biosciences) and V2 primers. Polymerase‐template complexes were bound to magnetic beads using the Mag‐bead Binding Kit (code: 100‐134‐800, Pacific Biosciences). Sequencing was performed on the Pacific Biosciences Real‐time Sequencer RSII using C3 sequencing reagents with a 3 h movie capture for each cell. Subread filtering was performed by the Pacific Biosciences SMRT Analysis software (v.2.3.0). The short read sequencing was performed on the Illumina sequencing platform at Beijing Genomics Institute (Shenzhen, Guangdong, China), including 250 bp paired‐end reads with the MiSeq technology and the 10 kb mate paired library. The raw data of Vd991 genome sequencing has been deposited in GenBank under PRJNA302216.

### Genome assembly

The genome assembly was performed using the PacBio SMRT Analysis software (v.2.3.0) with default parameters. The Miseq PE250 reads were assembled by the Newbler assembler (v.2.8, Roche) using default parameters. The lengths of the preassembled contigs were extended after the removal of redundant sequence by Rabbit (v.2.6, BGI). Subsequently, the contigs were combined into scaffolds using the Sspace tool (Boetzer *et al*., 2011) following sequencing of 10 kb mate paired reads, and the gaps were closed using the PBJelly program (English *et al*., 2012). Finally, the signal bases and insertion/deletion errors were corrected in the Miseq sequencing reads by SOAPsnp (v.1.03, http://soap.genomics.org.cn/) and Gatk (DePristo *et al*., 2011). This Whole Genome Shotgun project has been deposited at DDBJ/ENA/GenBank under the accession NVYA00000000. The version described in this paper is version NVYA01000000.

### Alignment and identification of rearrangements

Whole‐genome alignments were generated by the NUCmer program from the MUMmer 3.0 package (Kurtz *et al*., 2004) using the high‐coverage parameter setting (‐b 5000 ‐c 500 ‐l 200 ‐g 10 000). The unmatched scaffolds in the Vd991 genome were realigned to the JR2 reference genome by Blast (Altschul *et al*., 1997). TEs were identified by RepeatMasker (open 3.2.8, detailed parameters: ‐no_is, ‐norna, ‐engine, ‐s, ‐parallel = 1, used Repbase v.15.08) and RepeatProteinMask (‐noLowSimple, ‐pvalue = 1e–4) (http://www.repeatmasker.org). The rearrangements of Vd991 were identified by the assembled sequences that matched different physical locations on the JR2/VdLs.17 genomes according to the alignment results. The primers spanning the predicted breakpoints in any one of the three genotypes were designed for selective amplification, and control primers were designed to amplify the syntenic sequences that flanked the breakpoints among the three *V. dahliae* genomes. The rearrangements were verified by PCR reactions on genomic DNA from Vd991, JR2 and VdLs.17 with the corresponding primers (Table S1).

### Gene prediction and annotation

Protein‐coding genes in the Vd991 genome were predicted using a combination of *de novo*‐based and homology‐based approaches as well as transcript evidence. For the *de novo* prediction, the gene prediction of repeat‐masked genomes was implemented by GeneMark‐ES (Borodovsky & McIninch, 1993) together with *V. dahliae* (VdLs.17 and VdJR2)‐trained Augustus (v.2.6) (Stanke *et al*., 2008) and the Snap program (Korf, 2004). For the homology‐based prediction, the protein‐coding genes of VdLs.17, VaMs.102 (*V. alfalfae*, previously named ‘*V. albo‐atrum*’) (Klosterman *et al*., 2011), JR2 (de Jonge *et al*., 2013), representatives from three phenotypically diverse species/species complexes of *Fusarium* (*F. graminearum*,* F. verticillioides* and *F. oxysporum*) (Ma *et al*., 2010), *Nectria haematococca* (*= F. solani* species complex Mating Population VI) (Coleman *et al*., 2009), and *Magnaporthe oryzae* (Dean *et al*., 2005) were collected and mapped onto the Vd991 genome using tBlastn. Homologous sequences aligned to the matching proteins were defined as gene models of Vd991 using the GeneWise program (Birney & Durbin, 2000). The RNA‐seq data obtained in this study were mapped to the Vd991 genome by Tophat (Trapnell *et al*., 2009), and the transcriptome‐based gene structures were determined by Cufflinks (v.2.2.1, http://cole-trapnell-lab.github.io/cufflinks/). Finally, all gene evidence was combined using Glean (Mackey *et al*., 2005). The general annotation of predicted proteins was performed with the following programs: putative functional annotations were interrogated to known databases using Blastp to identify the best homologs, including the databases *nr*, eggNOGs (Powell *et al*., 2012) and InterProScan (incorporating InterPro, GO and KEGG pathway annotation) (Jones *et al*., 2014).

### Orthology and multigene family classification

The reciprocal Blast analysis of the genes in the three *V. dahliae* genomes was performed using the Blastp program (e‐value < 1e–7) to find all pairwise matches. The Solar software (v.0.9.6, http://sourceforge.net/p/treesoft/code/HEAD/tree/branches/dev/) was used to remove redundant members (match rate < 50% and identities < 50%). The pairwise matches from the Blast results were clustered using the clustering application of Hcluster_sg (Alexeyenko *et al*., 2006), and the gene families among the three *V. dahliae* genomes were classified by orthologous clustering. The gene synteny among the Vd991, JR2 and VdLs.17 coding sequences was conducted with MUMmer 3.0 (Kurtz *et al*., 2004) using NUCmer with default settings (except for ‐l 15 and ‐maxmatch). Protein‐coding genes without synteny among the three *V. dahliae* genomes, and having identities or coverage ratios < 50% were collected for unique gene statistical analysis.

### Pathogenicity‐related factor prediction

Secretomes of the three *V. dahliae* proteomes were identified using four programs commonly employed to identify protein localization. As described previously (Klosterman *et al*., 2011), all putative extracellular proteins containing a signal peptide but lacking transmembrane domains were identified as secreted proteins. The annotation of putative carbohydrate active enzymes (CAZymes) was performed using the HMM‐based routine of the Carbohydrate‐Active‐EnZYmes database (Cantarel *et al*., 2009). Significant hits compared with the CAZymes database were performed in the set of putative CAZymes using Blast (e‐value < 1e–5 and similarity ≥ 30%) and were used to increase the accuracy of the CAZyme annotation. CAZymes involved in plant cell wall degradation were collected according to the classification methods in previous publications (Battaglia *et al*., 2011; Dong *et al*., 2014). The homologs of known pathogen–host interaction (PHI) genes were predicted using the PHI database (v.3.6, http://www.phi-base.org/) (Winnenburg *et al*., 2008). The protein kinases (PKs) were predicted by running HMM searches locally with Kinomer (v.1.0) (Martin *et al*., 2009).

### Phylogenetic and evolutionary analysis

The orthologs among the *Verticillium* spp. (four isolates from two species) with the *Fusarium* spp. (15 isolates including *Nectria*) were analyzed using *Magnaporthe oryzae* as the outgroup, and 1313 single‐copy gene families (match rate ≥ 50% and identities ≥ 50%) were collected for phylogenetic analysis. The 1313 protein‐coding genes in each species were concatenated in the same order and aligned with Muscle (Edgar, 2004). Maximum‐likelihood trees were constructed to highlight the phylogenetic relationships between genes from *Verticillium* spp. and *Fusarium* spp. using Mega (v.6.0, 1000 bootstraps, using default parameters) (Tamura *et al*., 2013). LS gene sequences from Vd991 and their orthologs in 19 other species (identified by best hit based on ortholog clustering) were collected and aligned separately. Phylogenetic analyses were performed on each LS gene alignment using the method described earlier.

### Preparation of infected plant samples and transcriptome analyses

To obtain the transcriptome of *V. dahliae* isolates during root infection, plant roots were dipped into a conidial suspension, incubated for 24 h, and then washed from the roots and collected. In detail, conidia from 7‐d‐old cultures of *V. dahliae* grown on potato dextrose agar (PDA) were collected using a small volume of sterile distilled water (SDW), and the concentration of the inoculum was adjusted to 2 × 10^9^ conidia ml^−1^. Subsequently, the roots of 4‐wk‐old seedlings of cotton (*G. hirsutum* var. Junmian 1, susceptible to Vd991), tomato (*Solanum lycopersicum* var. MoneyMaker, susceptible to JR2), and lettuce (*Lactuca sativa* var. *Capitata*, susceptible to VdLs.17), were washed with SDW and submerged in the conidia suspension for 5 min, carefully removed, and transferred into a sterilized glass box at 25°C to maintain humidity. After 24 h, the inoculated plants were washed with 50 ml of SDW to collect the conidia covering the roots and centrifuged. The conidial suspension washed from a PDA plate was harvested as a control sample. Four *V. dahliae* libraries (three samples from host plants and one control sample) were prepared according to the TruSeq™ RNA Sample Preparation Kit (Illumina, San Diego, CA, USA) and libraries were sequenced using an Illumina Hiseq 2000 according to standard Illumina protocols. The raw data of RNA‐seq have been deposited in GenBank under PRJNA302216. The raw Fastq format datasets were produced using the software Casava v.1.8.2 with quality controls. Reads contaminated with Illumina adapters were detected and removed, and high‐quality reads (Phred score ≥ 20) were collected for further analysis. Differentially expressed genes were identified based on the significance of the false discovery rate < 0.00001, *P *<* *0.01 and |log_2_Ratio| ≥ ≥ 1.0. The pathways in which the differentially expressed genes (DEGs) were involved were identified with the Kyoto Encyclopedia of Genes and Genomes (KEGG) database (Kanehisa *et al*., 2012).

### Pathogenicity experiments

In overview, to test whether genes within GLSR‐2 were involved in virulence of cotton, lettuce, and tomato, several experiments were conducted with mutants of Vd991, JR2 and VdLs.17. The GLSR‐2 region was deleted in Vd991 and the virulence of these mutants on cotton, lettuce, and tomato were compared with the virulence of the wild‐type. Further, seven genes from Vd991 GLSR‐2 were independently transferred to both JR2 and VdLs.17, and these ectopic mutants were inoculated on cotton to test whether they enhanced virulence. Mutants with enhanced virulence to cotton were also inoculated on lettuce and tomato, to test whether the genes also enhanced virulence on these hosts. Seven genes (VEDA_05193 – VEDA_05199) in G‐LSR2 were deposited in GenBank under accession MF946582.

For gene deletion experiments, *c*. 1 kb of each of the two sequences flanking the deletion target was amplified from the wild‐type strain Vd991, and the hygromycin‐resistance element was amplified from the pCT‐Hyg vector. The three amplicons were fused to one fragment by a fusion PCR reaction with the reverse complementary adaptor, and a nested PCR reaction was performed with primers that contained gateway BP reaction adaptors. The final amplicon was cloned into the pGKO2‐Gateway vector (Khang *et al*., 2005) by a BP recombinant reaction (Invitrogen). The homolog recombinant vector was introduced into *Agrobacterium* strain AGL1 for fungal transformation. *V. dahliae* transformation was performed as previously described (Liu *et al*., 2013).

To reintroduce the LS genes into JR2 or VdLs.17, the DNA fragment containing the full‐length coding sequence, *c*. 1.5 kb of promoter region and *c*. 0.5 kb of the downstream sequence was amplified by PCR and cloned into the donor vector pCT‐HN (Liu *et al*., 2013). The pathogenicity and virulence of the targeted deletion mutants and ectopic transformants were determined using a root‐dip method. The strains were subcultured on PDA for 1 wk at 25°C. The inoculum was prepared by harvesting conidia and adjusting the concentration to 5 × 10^6^ condia ml^−1^ in sterile water. The cotton (*G. hirsutum* var. Junmian 1), tomato (*S. lycopersicum* var. MoneyMaker), and lettuce (*L. sativa* var. *Capitata*) were grown in soil in the glasshouse at 25°C with a photoperiod of 14 : 10 h, light : dark. Two‐week‐old seedlings of cotton and 4‐wk‐old seedlings of tomato and lettuce were uprooted and the roots were rinsed with water to remove the residual soil. Subsequently, the roots were dipped into the prepared conidial suspension for 5 min and the plants were transplanted into soil in pots. For cotton, 30 plants (five plants per pot) were inoculated per experiment, and for tomato and lettuce, 12 plants were inoculated per experiment; the experiment was conducted three times. The plants were scored at 4 wk postinoculation for symptoms. Fungal biomass was determined using the method described previously (Santhanam & Thomma, 2013). Quantitative reverse transcription polymerase chain reaction (qRT‐PCR) was performed using an ABI QuantStudio 6 Flex machine (Applied Biosystems, Foster City, CA, USA) in combination with the qPCR SYBR premix Ex Taq II kit (TaKaRa, Kusatsu, Shiga, Japan). *Verticillium* elongation factor 1‐α (*EF‐1*α) was used to quantify fungal colonization and the cotton *18S* gene was used as an endogenous plant control. The statistical significance of the fungal biomass data was assessed with unpaired Student's *t*‐tests.

## Results

### The Vd991 genome sequence revealed high sequence identity to the reference genomes but encoded many unique LS genes

The genome sequence of Vd991 was sequenced by combining PacBio RS II and Illumina sequencing technologies. The size of the genome was 34.8 Mb and consisted of 165 scaffolds, and its N50 length was 951.7 kb (Tables S2–S5; Fig. S2). Genome alignment with MUMmer (Kurtz *et al*., 2004) showed that 55 scaffolds with a total length of 33.9 Mb (> 97% genome size) in the Vd991 genome showed high synteny with the reference genomes of both JR2 and VdLs.17 (Table S6). Similar to the findings of a previous study that documented chromosomal rearrangements between JR2 and VdLs.17, 12 assembled sequences of Vd991 aligned to physical locations other than the breakpoints in the assembly sequence of JR2 or VdLs.17 genomes (Table S7). Eight and 10 rearrangement sites were identified in the genome of Vd991, compared with the JR2 and VdLs.17 genomes, respectively (Table S7). PCR validation of three breakpoints confirmed that the chromosomal rearrangements indeed occurred among the three *V. dahliae* genomes (Fig. S3).

**Figure 1:**
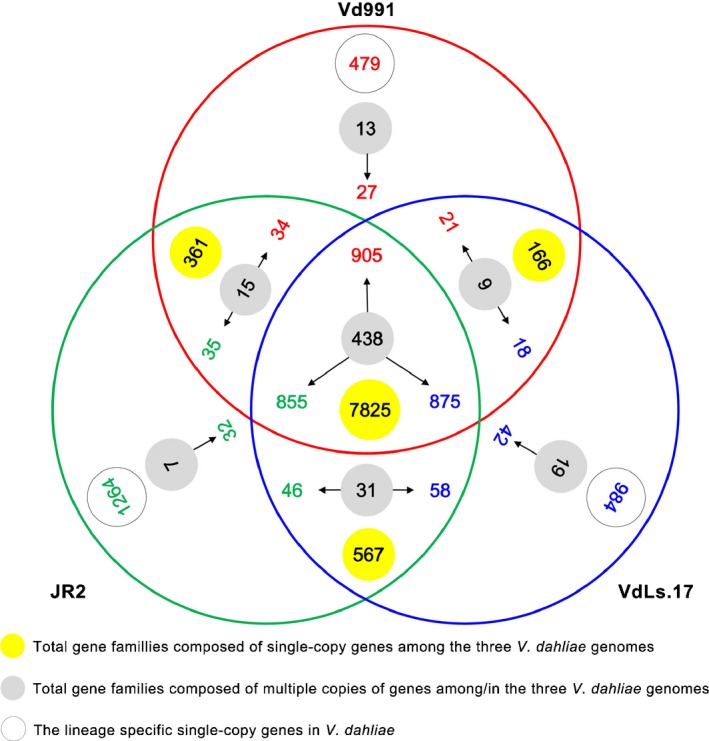
Ortholog gene clusters among the three *Verticillium dahliae* Vd991, JR2 and VdLs.17 genomes. The numbers in solid yellow circles represent the total gene families composed of single‐copy genes among the three *V. dahliae* genomes; the numbers in solid gray circles represent the total gene families composed of multiple copies of genes in the three *V. dahliae* genomes. Red, green and blue correspond to Vd991, JR2 and VdLs.17, respectively; the numbers in open gray circles represent the specific single‐copy genes in the Vd991, JR2 and VdLs.17 genomes.

By combining automated gene calling with *de novo*‐based and homology‐based prediction methods, we identified 9818 genes that were predicted to encode proteins (> 30 aa) in the genome of Vd991, 1167 and 717 less protein‐encoding genes than the JR2 and VdLs.17 genomes (Table S8), respectively. In detail, 7825 single copy and 438 multicopy genes were found in all three genomes, 361 single copy and 15 multicopy genes were shared between Vd991 and JR2, 166 single copy and nine multicopy genes were shared between Vd991 and VdLs.17, and 479 single copy and multicopy genes were exclusive to Vd991 (Fig. 1). While ortholog clustering analyses of all the protein‐coding genes showed that 7825 single‐copy genes were common to the three *V. dahliae* genomes, 905, 855, and 875 genes clustered into 438 multicopy gene families in Vd991, JR2 and VdLs.17, respectively (Fig. 1).

Large numbers of protein‐coding genes were identified as unique genes that were exclusive to specific *V. dahliae* strains by ortholog clustering: 506, 1296, and 1026 genes in Vd991, JR2, and VdLs.17 (Fig. 1), respectively (e.g. the Vd991 genome contained 479 unique single‐copy genes as well as 27 genes which grouped into 13 multicopy gene families). Additional analyses of protein coding gene sequences identified with Blast as having identities or coverage ratios of < 50% to genes in other *V. dahliae* genomes were classified as LS genes (Table S9). The suites of LS genes comprised 960, 1652 and 1390 genes in the genomes of Vd991, JR2 and VdLs.17 (Table S10), respectively. Thus, even though Vd991, JR2 and VdLs.17 belong to the same species and share high genomic sequence identity, gene content was significantly different as a result of the presence of many LS genes.

### Functional genomics of potential pathogenicity and virulence‐related factors in *V. dahliae*

The functional annotation of protein‐coding genes in Vd991 genome corroborates previous genomic studies of JR2 and VdLs.17, which showed expansions in gene families involved in carbohydrate transport and metabolism, energy production and conversion, and lipid transport and metabolism. These genes are postulated to have facilitated adaptation of *V. dahliae* to the unique ecological niche of the plant vascular system (Table S11). The Vd991 genome contained an arsenal of potential pathogenicity and virulence‐related factors including genes encoding secreted proteins, CAZymes, PHI proteins, transcription factors (TFs) and PKs, which may play significant roles in pathogenesis. In Vd991, 739 genes were predicted to encode secreted proteins, of which 127 hypothetical proteins encoded small cysteine‐rich proteins (< 400 amino acids, ≥ four cysteine residues), 605 genes encoded CAZymes, 268 were PHI proteins, 133 were TFs and 452 were PKs (Tables 1, S12). Although the genome of Vd991 had fewer protein‐coding genes, there was no evidence of loss of pathogenicity‐related factors compared with JR2 and VdLs.17 (Table S12). The multiscale comparison of the pathogenicity‐related factors encoded by LS genes highlights the notable differences among the three genomes (Tables S13–S15). However, the function of most LS genes is still unknown according to the annotation systems presently available (Table S16), which limits our complete understanding of the roles of LS genes in pathogenesis and host adaptation.

**Table 1:** Statistical analysis of the genes encoding pathogenicity‐related factors among the three *Verticillium dahliae* genomes

Annotation type	Total genes			Common genes^a^			Specific genes^a^		
	Vd991	JR2	VdLs.17	Vd991	JR2	VdLs.17	Vd991	JR2	VdLs.17
Secretome	739	767	749	624	629	629	62	62	43
CAZymes	273	284	284	248	253	254	10	11	7
Pectin degradation	65	62	56	58	55	51	4	3	1
Cellulose degradation	48	48	53	38	38	43	2	3	0
Hemicellulose degradation	19	23	26	18	23	25	1	0	1
Lignin degradation	37	38	36	35	36	34	0	2	0
PHI	49	47	50	41	42	41	2	1	3
SCRPs	127	115	123	96	87	87	21	13	13
LysM	3	3	4	3	3	3	0	0	1
NEP‐Like Protein	7	7	6	5	5	5	0	0	1
CAZymes	605	621	617	536	538	538	25	24	23
Pectin degradation	83	82	75	72	73	65	7	4	3
Cellulose degradation	77	76	76	63	62	63	3	6	1
Hemicellulose degradation	28	33	33	26	30	32	1	2	1
Ligin degradation	67	72	68	62	63	61	1	3	0
PHI	56	57	58	47	48	47	2	5	4
SCRPs	3	2	3	3	2	3	0	0	0
PHI	268	266	258	225	223	215	14	10	24
Pectin degradation	15	13	14	13	12	12	1	0	0
Cellulose degradation	12	12	12	8	10	8	0	2	0
Hemicellulose degradation	6	7	9	4	6	8	1	0	1
Ligin degradation	3	4	3	3	3	2	0	0	0

a Gene sequences with identities or coverage ratios < 50% of those of sequences in the other two genomes were designated ‘lineage‐specific’ genes. Only genes present in all three genomes were designated ‘common’ genes.

CAZymes, carbohydrate active enzymes; PHI, pathogen–host interaction; SCRPs, small cysteine‐rich proteins; LysM, lysin motifs; NEP, necrosis and ethylene‐inducing protein.

### *Verticillium dahliae* strains from different hosts of origin evolved diversified LSRs

**Figure 2:**
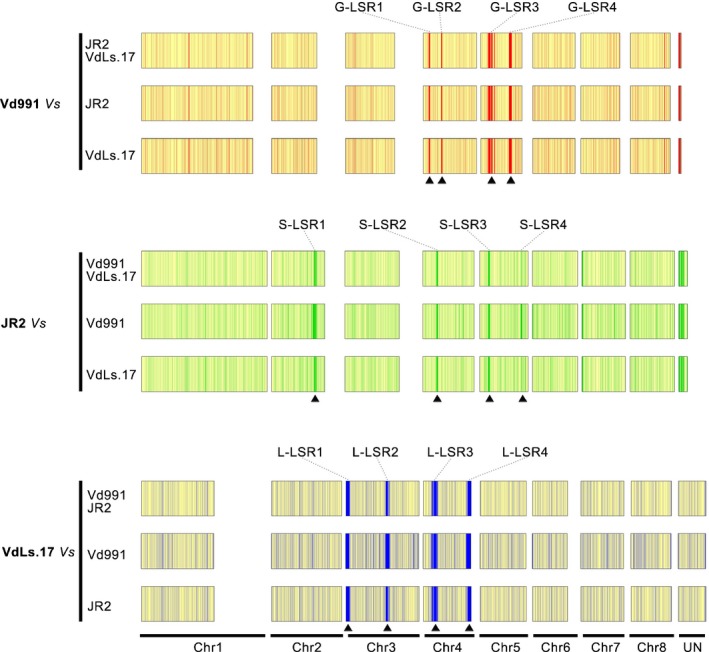
Presentation of lineage‐specific (LS) genes and gene synteny relationships among the three *Verticillium dahliae* strains Vd991, JR2 and VdLs.17. The genes were arranged by the ortholog clustering results with the physical location from chromosomes one to eight of the JR2 reference genome. UN indicates an unknown chromosome location of genes in *V. dahliae*. LS genes are marked in red, green and blue for Vd991, JR2 and VdLs.17, respectively. Triangular blocks indicate the LSRs.

Previous studies showed that the highly dynamic LSRs encoded many pathogenicity‐related genes, which contributed to virulence and niche adaptation in *V. dahliae* (Klosterman *et al*., 2011; de Jonge *et al*., 2013). Ortholog clustering and gene synteny analyses between Vd991 and JR2 revealed that many LS genes in Vd991 were significantly enriched in several narrow regions that formed the LSRs (Fig. 2), as previously reported in JR2 and VdLs.17 (Klosterman *et al*., 2011; de Jonge *et al*., 2013). In detail, at least four typical LSRs (G‐LSR1 through G‐LSR4) were identified in the Vd991 genome, which encoded 32, 23, 101 and 60 genes (Tables S17, S18), respectively. Investigations of gene function indicated that the LSRs are probably involved in virulence and niche adaptation as a result of the enrichment of pathogenicity‐related factors, e.g. the G‐LSR2 encoded 23 genes and six of them belong to secreted proteins, CAZymes, TFs, and PKs (Table S17). Furthermore, the LSRs were surrounded by transposons in Vd991 (Fig. S4), indicating that these regions are flexible and enable relatively rapid molecular evolution. While the LSRs in JR2 or VdLs.17 were enriched with pathogenicity‐related factors (Fig. 2; Table S17) (Klosterman *et al*., 2011; de Jonge *et al*., 2013), the three *V. dahliae* strains isolated from different hosts possessed differentiated LSRs, which provides evidence that pathogenicity‐related factors in the LSRs are associated with adaptation to different plants.

### Horizontal gene transfer is involved in the evolution of LSRs in *V. dahliae*

Although Vd991, JR2 and VdLs.17 belong to the same species, the genome of each strain encodes many exclusive LS genes (Tables S9, S10, S16). Further, 64 LS genes in Vd991 shared higher sequence identity with genes from other fungal genera rather than genes in the genomes of JR2 and VdLs.17 (e‐value < 1e–7, identities > 70%); 32 were homologs of genes found in *Fusarium* spp. genomes (Table S19). Interestingly, Vd991 LSRs appeared to be enriched with 21 that were homologous to genes in *Fusarium* spp., including 11 and six genes in G‐LSR2 and G‐LSR4, respectively (Table S19). These results indicated that some LS genes or portions of LSRs were probably acquired from other fungi, possibly *Fusarium* spp. that share ecological niches with *V. dahliae*, such as soil, rhizospheres, and plant vascular systems.

**Figure 3:**
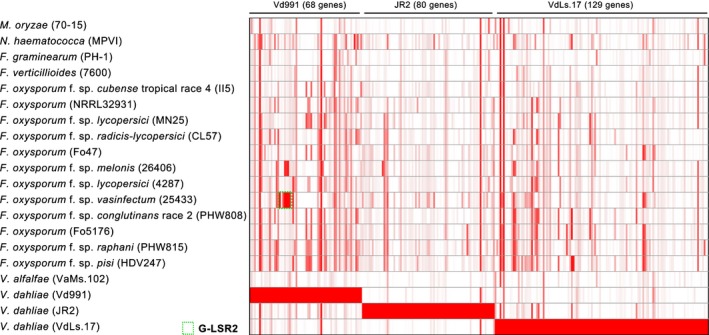
Homology analysis of lineage‐specific (LS) genes between *Verticillium dahliae* and *Fusarium* spp. Identity matrix of Vd991 LS genes and *Fusarium* spp. genes. The matrix was constructed using protein‐coding genes from Vd991 that had higher identities with *Fusarium* spp. genes than with the two other *V. dahliae* genes. The color gradient from white to red represents identities from 0 to 100%.

**Figure 4:**
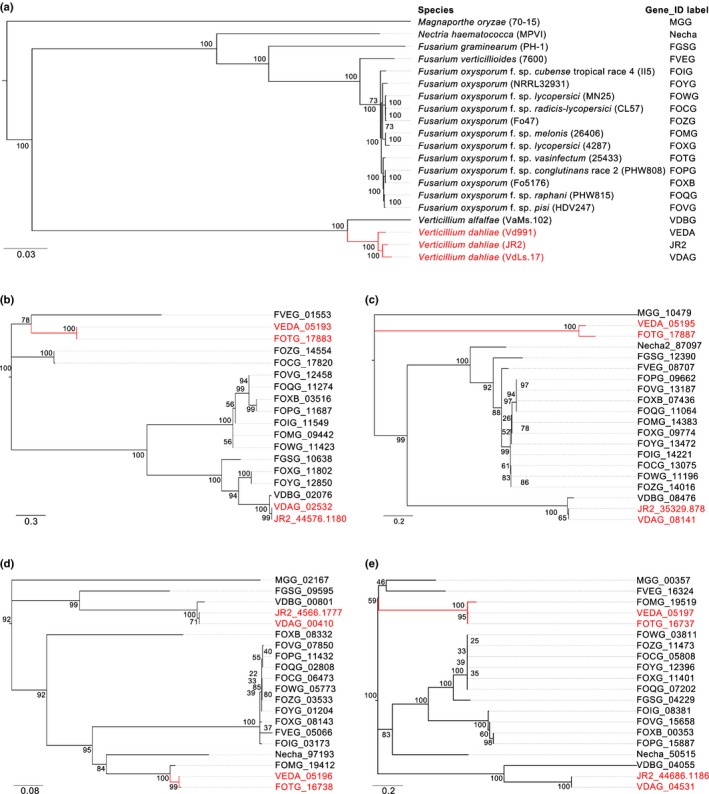
Phylogenetic relationships between lineage‐specific (LS) genes from *Verticillium dahliae* Vd991, JR2 d VdLs.17 and genes from *Fusarium* species. (a) Phylogenetic analyses were performed on nucleic acid sequences of orthologs of the single‐copy genes among 20 representative fusarias. *Magnaporthe oryzae* was set as the outgroup. *V. dahliae* strains are marked in red. (b–e) Evolutionary relationships between four LS genes from the lineage‐specific region G‐LRS2 in Vd991 and homologous genes in *Fusarium* spp. were inferred using the maximum likelihood method (1000 bootstraps). Genes for phylogenetic analysis of VEDA_05193 (b), VEDA_05195 (c), VEDA_05196 (d), and VEDA_05197 (e). Alphanumeric codes are standard gene accession numbers from reference genome databases. Clades that include genes from cotton wilt pathogens *V. dahliae* Vd991 and *Fusarium oxysporum* f. sp. *vasinfectum* are drawn in red.

To further investigate this possibility, all protein‐coding genes in Vd991 were systematically compared with the gene sets of 15 *Fusarium* spp. genomes (Broad Institute), which revealed that 68 Vd991 genes had higher identities to *Fusarium* spp. genes than to JR2 or VdLs.17 genes (Fig. 3; Table S20). Of these genes, seven LS genes (VEDA_05193‐VEDA_05199) in G‐LRS2 were extremely similar to the genes from *F. oxysporum* f. sp. *vasinfectum* NRRL 25433 (Fig. 3; Table S21). PCR validation confirmed that the seven genes within G‐LSR2 were present only in Vd991 and not in JR2 or VdLs.17 (Fig. S5). Contradicting the species phylogeny (Fig. 4a), phylogenetic analyses of the region comprising all seven protein‐coding genes VEDA_05193–VEDA_05199 indicated a close relationship to *F. oxysporum* f. sp*. vasinfectum* in all cases (Figs 4b–e, S6). *F. oxysporum* f. sp. *vasinfectum* is a soilborne fungus that also colonizes the plant vascular system and causes Fusarium wilt in cotton and often occupies the same soil niche as *V. dahliae*. Thus, it is plausible that Vd991 possessed the opportunity to acquire LS genes or G‐LSR2 from *F. oxysporum* f. sp. *vasinfectum* during coinfection of the cotton vascular system.

### Lineage‐specific region G‐LSR2 contributes to the adaptation of Vd991 to cotton

**Figure 5:**
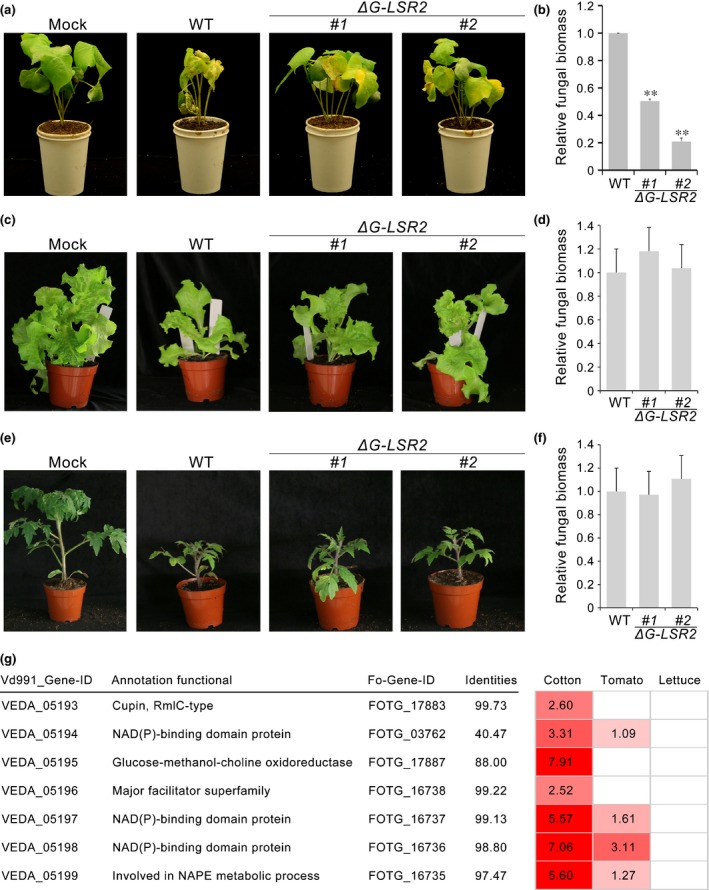
Identification of virulence factors in the lineage‐specific region (LSR) G‐LSR2 in Vd991. (a, c, e) Assessment of virulence of Vd991‐targeted deletion mutants after inoculation on cotton, lettuce, and tomato. Seven lineage‐specific (LS) genes in the Vd991 genomic region G‐LSR2 were knocked out; mutants were inoculated on cotton (a), lettuce (c), and tomato (e). The pathogenicity assay was carried out by the root‐dip methods using 2‐wk‐old seedlings of cotton and 4‐wk‐old seedlings of tomato and lettuce with 5 × 10^6^ condia ml^−1^ conidial suspension for 5 min. For cotton, 30 plants were inoculated per experiment, and for tomato and lettuce, 12 plants were inoculated per experiment; each experiment was conducted three times. Two independent targeted deletion strains (*∆G‐LSR2*) displayed reduced host adaptation of cotton compared with the wild‐type Vd991 at 21 d after inoculation. (b, d, f) Reduced fungal biomass accumulation of targeted deletion strains measured with quantitative reverse transcription polymerase chain reaction compared with wild‐type of Vd991 on cotton (b), lettuce (d), and tomato (f). The error bars represent SE; significant differences are indicated (unpaired Student's *t*‐test): **, *P *≤* *0.01. (g) Relative levels of transcription (log_2_Ratio) of seven LS genes within G‐LSR2 in Vd991during the first 24 h after infection in cotton, tomato and lettuce. Darker shades of red indicate higher levels of expression relative to expression in wild‐type Vd991.

To test the contribution of G‐LSR2 to the dominant adaption of Vd991 to cotton, knockout mutants of the region comprising all seven genes (VEDA_05193–VEDA_05199) were generated (Fig. S7). Remarkably, cotton plants inoculated with either of the two independent mutants showed significantly lower disease symptoms (Fig. 5a), and contained significantly less fungal biomass than those inoculated with the wild‐type strain (Fig. 5b). However, no change in symptom severity was observed on lettuce plants after inoculation with the mutants (Fig. 5c), and the fungal biomass was comparable to that of the wild‐type Vd991 strain (Fig. 5d). Deletion of GLSR‐2 genes from Vd991 similarly had little or no effect on its virulence on tomato (Fig. 5e,f). These results strongly suggested that, as a result of the deletion of seven genes, the mutants lost the dominant adaptation to the original host cotton, but their virulence on other hosts was not changed.

Additionally, RNA sequencing of Vd991 during root infection showed that the transcript abundances of the seven genes in G‐LSR2 were positively correlated to the virulence of Vd991 to cotton. In detail, all seven genes were highly up‐regulated during infection of cotton (log_2_Ratio from 2.52 to 7.91), while a lower degree of up‐regulation of only four genes was observed in tomato, and no up‐regulation was observed in lettuce (Figs 5g, S8). Thus, the LSR G‐LSR2 acquired from the Fusarium wilt pathogen, *F. oxysporum* f. sp. *vasinfectum*, greatly contributed to the virulence of Vd991 in cotton.

### Transfer of three genes from LSR G‐LSR2 to JR2 and VdLs.17 enhanced virulence to cotton but did not enhance virulence to lettuce or tomato

**Figure 6:**
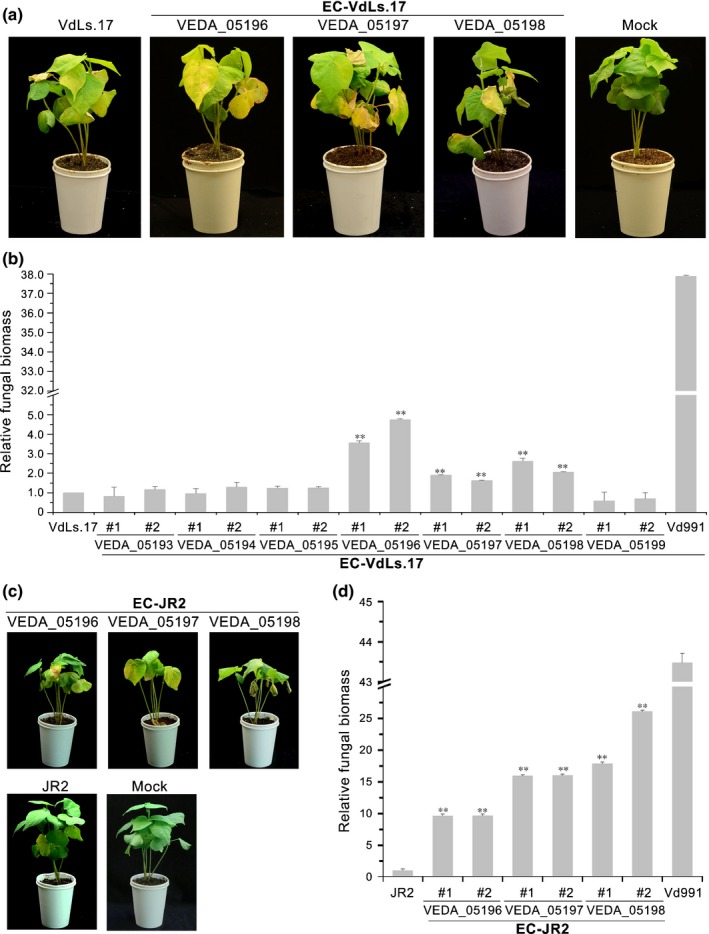
Transfer of genes from lineage‐specific region G‐LSR2 from Vd991 to VdLs.17 and JR2‐enhanced virulence and host adaptation to cotton. (a) Cotton pathogenicity assays using transformants of VdLs.17 strains that separately acquired each of the seven genes from Vd991 G‐LSR2. Separate introduction of three lineage‐specific (LS) genes (VEDA_05196, VEDA_05197 or VEDA_05198) from G‐LSR2 to VdLs.17 enhanced the virulence (host adaptation) on cotton. The pathogenicity assay was carried out by the root‐dip methods using 2‐wk‐old seedlings of cotton with 5 × 10^6^ condia ml^−1^ conidial suspension for 5 min, and 30 plants were inoculated per experiment; the experiment was conducted three times. (b) The development of fungal biomass of two independent ectopic transformants compared with wild‐type VdLs.17 21 d after inoculation. The error bars represent SE, and significant differences are indicated (unpaired Student's *t*‐test): **, *P *≤* *0.01. (c) Cotton pathogenicity assays using transformants of JR2 strain that separately acquired VEDA_05196, VEDA_05197 or VEDA_05198. The pathogenicity assay was carried out by the root‐dip methods using 2‐wk‐old seedlings of cotton with 5 × 10^6^ condia ml^−1^ conidial suspension for 5 min; 30 plants were inoculated per experiment and the experiment was conducted three times. (d) The development of fungal biomass of two independent ectopic transformants of JR2 compared with wild‐type JR2 21 d after inoculation. The error bars represent SE, and significant differences are indicated (unpaired Student's *t*‐test): **, *P *≤* *0.01.

The LSR G‐LSR2 was present in the genome of Vd991 but was absent in JR2 and VdLs.17 (Fig. 2). Strains JR2 and VdLs.17 showed high virulence to their host of origin but were less virulent to cotton (Fig. S1), implicating G‐LSR2 as the genetic basis of the dominant adaptation of Vd991 to cotton. Separate transfers of each of the seven genes from Vd991 G‐LSR2 into strain VdLs.17 were performed and subsequent virulence tests demonstrated that three genes resulted in enhanced virulence in cotton (Fig. 6a). The VdLs.17 transformants that received VEDA_05196, VEDA_05197, or VEDA_05198 from Vd991 G‐LSR2 were more virulent on cotton than on the wild‐type VdLs.17 (Fig. 6a). Quantification of fungal biomass by qRT‐PCR confirmed that the ectopic strains accumulated significantly more fungal biomass in cotton than in the wild‐type VdLs.17 (Fig. 6b). Pathogenicity experiments with JR2 transformants confirmed that VEDA_05196, VEDA_05197, and VEDA_05198 each conferred to the tomato strain the ability to infect cotton (Fig. 6c,d). The VEDA_05196 transformants caused more severe symptoms and developed more fungal biomass in cotton plants (Fig. 6), but still much less biomass than wild‐type Vd991 on cotton, suggesting that multiple G‐LSR2 genes are required for the observed virulence of Vd991 in cotton.

**Figure 7:**
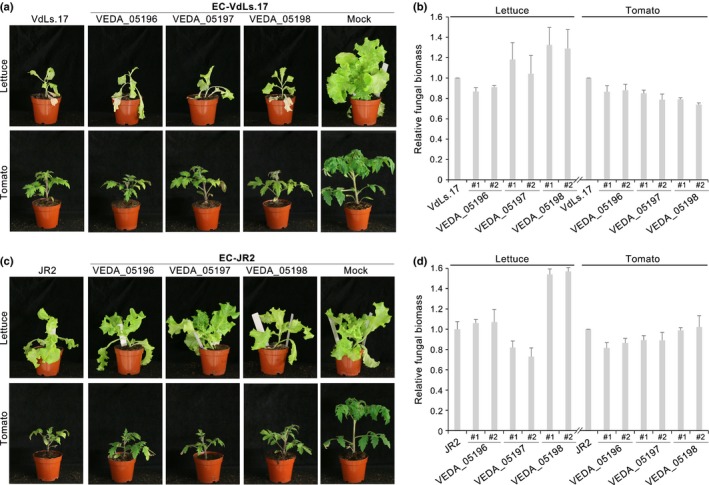
Transfer of single gene from Vd991 G‐LSR2 to VdLs.17 or JR2 did not result in increased virulence on lettuce and tomato. (a, c) Disease severity of lettuce and tomato plants inoculated with VdLs.17 (a) or JR2 transformants (c) 21 d after inoculation. The pathogenicity assay was carried out by the root‐dip methods using 4‐wk‐old seedlings of tomato and lettuce with 5 × 10^6^ condia ml^−1^ conidial suspension for 5 min; 12 plants were inoculated per experiment and the experiment was conducted three times. The transformants were generated by separate introduction of three lineage‐specific (LS) genes (VEDA_05196, VEDA_05197 or VEDA_05198) from Vd991 G‐LSR2 to VdLs.17 and JR2. (b, d) The development of fungal biomass *in planta* of two independent ectopic transformants of VdLs.17 (b) and JR2 (d) compared with the wild‐types; the error bars represent SE.

Transgenic JR2 and VdLs.17 mutants that received LS genes from Vd991 did not display enhanced virulence on lettuce or tomato. Independent transgenic VdLs.17 strains that received VEDA_05196, VEDA_05197, or VEDA_05198 from Vd991 G‐LSR2 caused wilt symptoms similar to the wild‐type VdLs.17 strain on lettuce and tomato plants (Fig. 7a). Fungal biomass accumulation *in planta* was similar across the transformants, although VEDA_05198 transformants developed slightly higher fungal biomass (Fig. 7b). Likewise, the JR2 transformants that received VEDA_05196, VEDA_05197, or VEDA_05198 from Vd991 G‐LSR2 caused disease symptoms similar to the wild‐type JR2 on lettuce and tomato; VEDA_05198 transformants developed slightly more fungal biomass in lettuce but not on tomato plants (Fig. 7c,d). These results suggested that the LSR G‐LSR2 in *V. dahliae* conferred a host adaptation only towards cotton.

Functional annotation of genes in G‐LSR2 showed that VEDA_05196 encoded a major facilitator superfamily protein, and VEDA_05197 and VEDA_05198 contained an NAD(P)‐binding domain (Fig. 5g), which are generally involved in redox reactions that facilitate pathogen escape from host defense responses involving oxidative stress. Therefore, genes from G‐LSR2 may enable *V. dahliae* to adapt to oxidative stress during infection of cotton.

## Discussion

*Verticillium dahliae* is a notorious pathogen that causes serious economic losses in high‐value agricultural crops. The genome sequences of JR2 and VdLs.17 facilitated investigation into the mechanisms underlying virulence and niche adaptation, including the expansion of pathogenicity‐related factors and the evolution of highly dynamic LS regions via extensive chromosomal rearrangements (Klosterman *et al*., 2011; de Jonge *et al*., 2013). Although *V. dahliae* is a soilborne plant pathogen with a broad host range (over 200 dicotyledonous plant species) (Fradin & Thomma, 2006), different strains vary in virulence on different hosts (Bhat & Subbarao, 1999). Generally, symptoms induced by a strain on its host of origin are more severe than symptoms on other hosts (Bhat & Subbarao, 1999; Vallad *et al*., 2016). This has been described as the dominant adaptation to the host of origin. Mechanisms underlying the dominant adaptation to the host of origin remain unclear. In this study, pathogenicity tests demonstrated that the strain Vd991, a typical *V. dahliae* strain isolated from cotton, was highly aggressive on cotton plants compared with either JR2 isolated from tomato or VdLs.17 isolated from lettuce (Fig. S1). Based on the high‐quality genome sequence of Vd991 (Fig. S2; Table S5), we elucidated the genetic basis of the dominant adaptation to cotton in *V. dahliae* through comparative genomics of the three strains.

The major molecular underpinnings of pathogenicity in *Verticillium* spp. have been described over the past 10 yr. Several factors involved in virulence and host adaptation have been identified, including secreted proteins, carbohydrate enzymes, PKs, TFs, metabolism‐related enzymes, etc. (Klimes *et al*., 2015). Comparative genomics have revealed that the *Verticillium* spp. have an outstanding capacity to degrade plant cell walls by the expansion of some CAZyme families, and that flexible genomic islands enriched for TEs and LS genes contribute to the development of virulence and host adaptation (Klosterman *et al*., 2011; de Jonge *et al*., 2013). Our results indicated that the *V. dahliae* genomes investigated encoded a large number of pathogenicity and virulence‐related factors that probably contribute to the adaptation to the plant vascular system. Furthermore, genomes of *V. dahliae* isolated from different hosts encoded many unique LS genes, which were functionally relevant to plant pathogenicity. The annotation of the seven genes within GLSR‐2 suggests a role in defense against oxidative stress, possibly related to the oxidative burst defense response observed in many plants (Robb *et al*., 2012).

In the current study, although the total number of protein‐coding genes was significantly different among the three *V. dahliae* genomes, a systematic comparison of gene counts indicated that the number of pathogenicity‐related factors were neither significantly expanded nor contracted as deduced from the multilayered statistics employed here (Tables 1, S13–S15). Evidence from orthologous clustering analyses suggested that the variability in virulence among *V. dahliae* isolated from different hosts could be explained by the large quantities of unique LS genes between strains (Fig. 1; Table S10). Comparative genomics confirmed that genetic flexibility is the likely mechanism by which many novel LS genes were generated within the three genomes of *V. dahliae* studied here (Fig. 1; Table S10). These results indicated that the LS genes of *V. dahliae* play significant roles in host–fungal interactions and pathogenesis. However, functional annotations of LS genes are currently limited and thus cannot fully explain host‐associated variation in virulence, as most of the genes were novel with no clear functions (Table S16).

Various mechanisms underlying the evolution of host adaptation (Felsenstein, 1974) in this asexual plant pathogen have been proposed based on large‐scale genomic and functional comparisons, including enrichment for TEs (Ohm *et al*., 2012; de Jonge *et al*., 2013; Kellner *et al*., 2014), extensive chromosomal rearrangements (Coleman *et al*., 2009; de Wit *et al*., 2012; de Jonge *et al*., 2013), additional dispensable chromosomes (Ma *et al*., 2010; Stukenbrock *et al*., 2010; Goodwin *et al*., 2011; Raffaele & Kamoun, 2012), a high proportion of positively selected genes (O'Connell *et al*., 2012; Brunner *et al*., 2013; Dong *et al*., 2014; Kellner *et al*., 2014; Sharma *et al*., 2014), horizontal gene transfer (HGT) (Mehrabi *et al*., 2011; Dhillon *et al*., 2015), gene loss or expansion (Haas *et al*., 2009; Baxter *et al*., 2010; Klosterman *et al*., 2011; O'Connell *et al*., 2012; Ohm *et al*., 2012; Gan *et al*., 2013; Sharma *et al*., 2014; Zhang *et al*., 2014), and gene‐sparse regions (Daboussi & Capy, 2003; Haas *et al*., 2009; Raffaele *et al*., 2010). A previous study suggested that chromosomal rearrangement is an important mechanism in the evolution of *V. dahliae*, which could rapidly evolve novel effector genes that contribute to virulence and niche adaptation (Klosterman *et al*., 2011; de Jonge *et al*., 2013). In the current study, although the genome content (size, GC%, etc.) of Vd991 was highly similar to the genome content of JR2 and VdLs.17 (Table S8), whole‐genome alignments showed that 12 intact assembly sequences of the Vd991 genome were located on different reference chromosomes (Fig. S3; Table S7). This observation supports the notion that chromosomal rearrangements could occur frequently and represent a mechanism of evolution of host adaptation and virulence.

Horizontal gene transfer has been shown to play an important role in host adaptation (Daboussi & Capy, 2003), especially between pathogens that cohabit the same unique ecological niche (Mehrabi *et al*., 2011). The transfer of a gene encoding a host selective toxin (*ToxA*) from *Stagonospora nodorum* that conferred virulence to *Pyrenophora tritici‐repentis* on wheat (Friesen *et al*., 2006) is one such example. Both VdGT2 and *Ave1* were acquired by *Verticillium* through horizontal gene transfer as well (Klimes *et al*., 2015). However, clear experimental evidence indicating that the transferred genetic material from another related or unrelated fungal species is lacking (Mehrabi *et al*., 2011). *V. dahliae* and *F. oxysporum* f. sp*. vasinfectum* share the same ecological niche of the plant vascular system as well as in the soil environment during survival and plant colonization. These habitats offer opportunities to exchange genetic material between such pathogens. Transfer of specific genome fragments from other fungi that are well adapted to the same environment could be a simple and efficient mechanism for *V. dahliae* to improve its host adaptation. In Vd991, several LS genes showed much higher similarity to genes in other fungal species (*Fusarium* spp., *Colletotrichum* spp., etc.) than with JR2 and VdLs.17 (Tables S19, S20). The phylogenetic analyses strongly supported the idea that several LS genes were probably acquired by horizontal gene transfers (Figs 4, S6). Blast and phylogenetic analyses found that part of LSR G‐LSR2 very probably originated from *F. oxysporum* f. sp. *vasinfectum*. Targeted deletion of genes in Vd991 G‐LSR2 resulted in reduced virulence to cotton (Fig. 5a,b), but did not affect their virulence on lettuce and tomato (Fig. 5c–f). Furthermore, strain VdLs.17 became virulent on cotton following transfer of part of LS genes from G‐LSR2 (Fig. 6), but the ectopic transformants (including ectopic expression of single genes from G‐LSR2 in JR2) did not appear to enhance virulence on lettuce and tomato (Fig. 7). This experimental evidence strongly suggests that Vd991 obtained the dominant adaptation to cotton through the horizontal transfer of LS genes or LSRs from *F. oxysporum* f. sp. *vasinfectum*. The annotation of the genes in G‐LSR2 suggested that they may protect the pathogen against host defense responses involving oxidative stress. However, it is currently unclear why these genes appeared to function specifically during cotton infection, as oxidative bursts are a common component in many plant defense responses.

In summary, the genome of Vd991, originally isolated from cotton, was sequenced and systematically compared with the reference genomes of JR2 and VdLs.17. The Vd991 genome showed very high sequence identity to the two reference genomes but encoded fewer genes. Comparative genomics indicated that significant expansions and contractions of shared pathogenicity‐related factors did not occur among the three *V. dahliae* genomes. Rather, the genome of Vd991 encoded many LS genes and a subset of them comprised the typical LSRs that were also present in the genomes of JR2 and VdLs.17. The seven‐gene fragment within G‐LSR2 in Vd991, acquired by horizontal gene transfer from *F. oxysporum* f. sp. *vasinfectum*, the cotton Fusarium wilt pathogen, contributed to the dominant adaptation of *V. dahliae* to cotton. Our comparative and functional genomics analyses among the three *V. dahliae* genomes from different hosts offered new insights into mechanisms of genetic variation and revealed the genetic basis of the dominant adaptation to a host of origin in *V. dahliae*.

## Author contributions

X‐F.D., G‐Y.Z. and K.V.S. conceived and designed the experiments. J‐Y.C., Y‐J.G., D‐D.Z., J.W., N‐Y.L., W‐Q.Z., L.Z. and B‐L.W. performed the experiments. J‐Y.C., C.L., K‐W.S., D.P.G.S., J‐Q.H., Y.L. and L.Y. analyzed the data. X‐F.M., T‐G.L. and Y‐M.B. prepared biological material. J‐Y.C., C.L., K‐W.S., D.P.G.S., J‐Q.H., Y.L. and L.Y. conducted the bioinformatic analysis. C.L., J‐Q.H., N‐Y.L. and X‐F.M. conducted the transcriptome analyses. X‐F.D., G‐Y.Z. and J‐Y.C. wrote the original draft. D.P.G.S. and K.V.S. edited and re‐wrote parts of the manuscript.

## Supporting information

Please note: Wiley Blackwell are not responsible for the content or functionality of any Supporting Information supplied by the authors. Any queries (other than missing material) should be directed to the *New Phytologist* Central Office.

**Fig. S1** Virulence phenotypes of hosts inoculated with *Verticillium dahliae*.
**Fig. S2** Lengths of scaffolds and GC content in the assembled Vd991 genome sequence.
**Fig. S3** Verification of rearrangement sites among the three *Verticillium dahliae* strains (Vd991, JR2 and VdLs.17) by PCR.
**Fig. S4** The distribution of transposable elements (TEs) in the lineage‐specific regions (LSRs) in the genome of *Verticillium dahliae* Vd991.
**Fig. S5** PCR verification of the lineage‐specific (LS) genes in putative *Gossypium* lineage‐specific region G‐LSR2 compared with the isolates JR2 and VdLs.17. F1–F5 represent five arbitrary fragments in G‐LSR2; M, 1 kb DNA ladder.
**Fig. S6** Phylogenetic relationships between Vd991 lineage‐specific (LS) genes within putative *Gossypium* lineage‐specific region G‐LSR2 and orthologous genes from *Fusarium* spp.
**Fig. S7** Gene expression patterns of genes harbored in the four putative *Gossypium* lineage‐specific regions (G‐LSRs 1‐4) of the genome of *Verticillium dahliae* Vd991 24 h after inoculation on cotton, tomato and lettuce roots.
**Fig. S8** Validation of the targeted deletions of G‐LSR2 in *Verticillium dahliae* Vd991 by PCR.
**Table S1** Primers used in the study
**Table S2** PacBio RS II and Illumina raw data of genome sequences
**Table S3** Key parameters of the genome assembly of the *Verticillium dahliae* strain Vd991by PacBio RS II biotechnology
**Table S4** Improvement in genome sequence quality of *Verticillium dahliae* strain Vd991 with Illumina MiSeq data
**Table S5** Key parameters of the genome assembly of the *Verticillium dahliae* Vd991
**Table S6** Physical locations of *Verticillium dahliae* Vd991 genomic regions relative to reference genomes of *V. dahliae* JR2 and VdLs.17
**Table S7** Location of rearrangements in the *Verticillium dahliae* Vd991 genome compared with JR2 and VdLs.17 genomes
**Table S8** Comparison of gene models among the three genomes of *Verticillium dahliae* Vd991, JR2, and VdLs.17
**Table S9** Gene synteny among the three genomes of *Verticillium dahliae* Vd991, JR2, and VdLs.17
**Table S10** Analysis and comparisons of specific gene content among the three genomes of *Verticillium dahliae* Vd991, JR2, and VdLs.17
**Table S11** Fungi nonsupervised orthologous groups (fuNOG) annotations of protein coding genes among the three genomes of *Verticillium dahliae* Vd991, JR2, and VdLs.17
**Table S12** Functional annotation of potential pathogenicity and virulence‐related factors among the three genomes of *Verticillium dahliae* Vd991, JR2, and VdLs.17
**Table S13** Classification of the subfamilies of CAZymes in the genomes of the three *Verticillium dahliae* isolates Vd991, JR2, and VdLs.17
**Table S14** Protein kinase annotation among the three genomes of *Verticillium dahliae* isolates Vd991, JR2 and VdLs.17
**Table S15** Annotation of transcription factors among the three genomes of *Verticillium dahliae* isolates Vd991, JR2, and VdLs.17
**Table S16** Percentages of protein‐coding genes with functional annotations in the genomes of the three *Verticillium dahliae* isolates Vd991, JR2, and VdLs.17.
**Table S17** Pathogenicity‐related factors in the lineage‐specific regions (LSRs) in the genomes of *Verticillium dahliae* isolates Vd991, JR2 and VdLs.17
**Table S18** Annotations of the protein‐coding genes within LSRs among the three genomes of *Verticillium dahliae* isolates Vd991, JR2 and VdLs.17
**Table S19** List of Vd991 genes homologous to genes from fungal genera other than *Verticillium*
**Table S20** List of genes in certain *Verticillium dahliae* strains with best hits to protein‐coding genes in *Fusarium*
**Table S21** Blast analysis of seven genes in putative *Gossypium* lineage‐specific region G‐LSR2 by the *nr* database
