# Supplementary material for: Comparative genomics reveals cotton‐specific virulence factors in flexible genomic regions in Verticillium dahliae and evidence of horizontal gene transfer from Fusarium
Source: New Phytol. 2017 Oct 30;217(2):756–70. doi: 10.1111/nph.14861 (PMC5765495; doi:10.1111/nph.14861)
Supplement: Supplementary file 1 — Fig. S1 Virulence phenotypes of hosts inoculated with Verticillium dahliae. Fig. S2 Lengths of scaffolds and GC content in the assembled Vd991 genome sequence. Fig. S3 Verification of rearrangement sites among the three Verticillium dahliae strains (Vd991, JR2 and VdLs.17) by PCR. Fig. S4 The distribution of transposable elements (TEs) in the lineage‐specific regions (LSRs) in the genome of Verticillium dahliae Vd991. Fig. S5 PCR verification of the lineage‐specific (LS) genes in putative Gossypium lineage‐specific region G‐LSR2 compared with the isolates JR2 and VdLs.17. F1–F5 represent five arbitrary fragments in G‐LSR2; M, 1 kb DNA ladder. Fig. S6 Phylogenetic relationships between Vd991 lineage‐specific (LS) genes within putative Gossypium lineage‐specific region G‐LSR2 and orthologous genes from Fusarium spp. Fig. S7 Gene expression patterns of genes harbored in the four putative Gossypium lineage‐specific regions (G‐LSRs 1‐4) of the genome of Verticillium dahliae Vd991 24 h after inoculation on cotton, tomato and lettuce roots. Fig. S8 Validation of the targeted deletions of G‐LSR2 in Verticillium dahliae Vd991 by PCR. Table S1 Primers used in the study Table S2 PacBio RS II and Illumina raw data of genome sequences Table S3 Key parameters of the genome assembly of the Verticillium dahliae strain Vd991by PacBio RS II biotechnology Table S4 Improvement in genome sequence quality of Verticillium dahliae strain Vd991 with Illumina MiSeq data Table S5 Key parameters of the genome assembly of the Verticillium dahliae Vd991 Table S6 Physical locations of Verticillium dahliae Vd991 genomic regions relative to reference genomes of V. dahliae JR2 and VdLs.17 Table S7 Location of rearrangements in the Verticillium dahliae Vd991 genome compared with JR2 and VdLs.17 genomes Table S8 Comparison of gene models among the three genomes of Verticillium dahliae Vd991, JR2, and VdLs.17 Table S9 Gene synteny among the three genomes of Verticillium dahliae Vd991, JR2, and VdLs.17 [file NPH-217-756-s001.pdf]

New Phytologist Supporting Information Figs S1–S8, Tables S1-S21

Article title: **Comparative Genomics Reveals Cotton-specific Virulence Factors in Flexible Genomic Regions in *Verticillium dahliae* and Evidence of Horizontal Gene Transfer from *Fusarium***

Authors: Jie-Yin Chen[1#], Chun Liu[2#], Yue-Jing Gui[1#], Kai-Wei Si[2#], Dan-Dan Zhang[1#], Jie Wang[1#], Dylan P. G. Short[3], Jin-Qun Huang[2], Nan-Yang Li[1], Yong Liang[2], Wen-Qi Zhang[1], Lin Yang[2], Xue-Feng Ma[1], Ting-Gang Li[1], Lei Zhou[1], Bao-Li Wang[1], Yu-Ming Bao[1], Krishna V. Subbarao[3*], Geng-Yun Zhang[2*], Xiao-Feng Dai[1*]

Article acceptance date: 21 September 2017

The following Supporting Information is available for this article:

Fig. S1 Virulence phenotypes of hosts inoculated with *Verticillium dahliae*.

Fig. S2 Lengths of scaffolds and GC content in the assembled Vd991 genome sequence.

Fig. S3 Verification of rearrangement sites among the three *V. dahliae* strains (Vd991, JR2 and VdLs.17) by PCR.

Fig. S4 The distribution of transposable elements (TEs) in the lineage specific regions (LSRs) in the genome of *V. dahliae* Vd991.

Fig. S5 PCR verification of the lineage specific (LS) genes in putative *Gossypium* lineage specific region G-LSR2 compared to the isolates JR2 and VdLs.17. F1 to F5 represents five arbitrary fragments in G-LSR2; M, 1 kb DNA ladder.

Fig. S6 Phylogenetic relationships between Vd991 lineage specific (LS) genes within putative *Gossypium* lineage specific region G-LSR2 and orthologous genes from *Fusarium* spp.

Fig. S7 Gene expression patterns of genes harbored in the four putative *Gossypium* lineage specific regions (G-LSRs 1-4) of the genome of *Verticillium dahliae* Vd991 24 hours after inoculation on cotton, tomato and lettuce roots.

Fig. S8 Validation of the targeted deletions of G-LSR2 in *Verticillium dahliae* Vd991 by PCR.

Table S1 Primers used in the study.

Table S2 PacBio RS II and Illumina raw data of genome sequences.

Table S3 Key parameters of the genome assembly of the *Verticillium dahliae* strain Vd991by PacBio RS II biotechnology.

Table S4 Improvement in genome sequence quality of *Verticillium dahliae* strain Vd991 with Illumina MiSeq data.

Table S5 Key parameters of the genome assembly of the *Verticillium dahliae* Vd991.

Table S6 Physical locations of *Verticillium dahliae* Vd991 genomic regions relative to reference genomes of *V. dahliae* JR2 and VdLs.17.

Table S7 Location of rearrangements in the *Verticillium dahliae* Vd991 genome compared to JR2 and VdLs.17 genomes.

Table S8. Comparison of gene models in the three genomes of *Verticillium dahliae* Vd991, JR2 and VdLs.17.

Table S9 Gene synteny among the three genomes of *Verticillium dahliae* Vd991, JR2, and VdLs.17.

Table S10 Analysis and comparisons of specific gene content among the three genomes of *Verticillium dahliae* Vd991, JR2, and VdLs.17.

Table S11 Fungi non-supervised orthologous groups (fuNOG) annotations of protein coding genes among the three genomes of *Verticillium dahliae* Vd991, JR2, and VdLs.17.

Table S12 Functional annotation of potential pathogenicity and virulence-related factors among the three genomes of *Verticillium dahliae* Vd991, JR2, and VdLs.17.

Table S13 Classification of the sub-families of CAZymes in the genomes of the three *Verticillium dahliae* isolates Vd991, JR2, and VdLs.17.

Table S14 Protein kinase annotation among the three genomes of *Verticillium dahliae* isolates Vd991, JR2 and VdLs.17.

Table S15 Annotation of transcription factors among the three genomes of *Verticillium dahliae* isolates Vd991, JR2, and VdLs.17.

Table S16 Percentages of protein-coding genes with functional annotations in the genomes of the three *Verticillium dahliae* isolates Vd991, JR2, and VdLs.17.

Table S17 Pathogenicity-related factors in the lineage specific regions (LSRs) in the genomes of *Verticillium dahliae* isolates Vd991, JR2 and VdLs.17.

Table S18 Annotations of the protein-coding genes within LSRs among the three genomes of *Verticillium dahliae* isolates Vd991, JR2 and VdLs.17.

Table S19 List of Vd991 genes homologous to genes from fungal genera other than *Verticillium*.

Table S20 List of genes in certain *Verticillium dahliae* strains with best hits to protein-coding genes in *Fusarium*.

Table S21 BLAST analysis of seven genes in putative *Gossypium* lineage specific region G-LSR2 by the *nr* database.

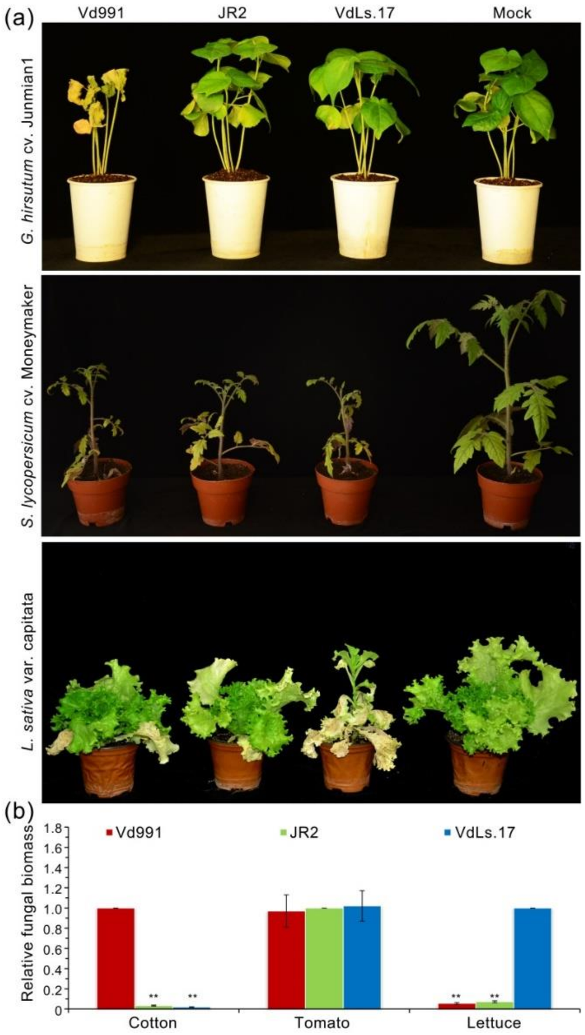

Fig. S1 Virulence phenotypes of hosts inoculated with *Verticillium dahliae*. (a) The *V. dahliae* strains Vd991, JR2 and VdLs.17 originated from cotton, tomato and lettuce, respectively, and were used to infect cotton, tomato and lettuce. For the *V. dahliae* inoculations, two-week-old seedlings of the host plants were mock inoculated (control) or inoculated with 3 mL suspension of 5×10^6 condia/ml in sterile water using a root-dip method. Photographs of the virulence phenotypes were taken 14 days after *V. dahliae* inoculation. **(b)** Detection of fungal biomass in three host plants inoculated with the three *V. dahliae* strains. Roots of three inoculated plants were harvested at 14 days after *V. dahliae* inoculation, ground to powder, and genomic DNA was isolated. The relative fungal biomass was detected by using Real-time PCR on genomic DNA. Error bars represent standard errors. **represent statistical significance ($P < 0.01$), according to unpaired Student's *t*-tests.

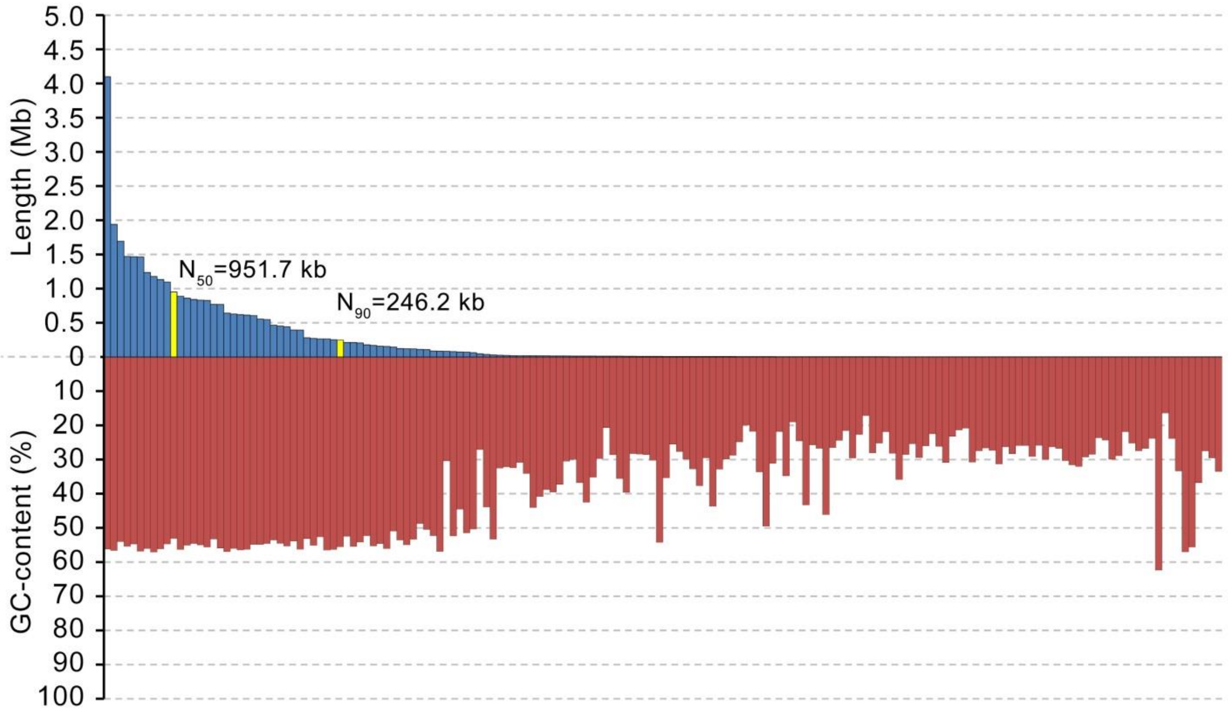

Fig. S2 Lengths of scaffolds and GC content in the assembled Vd991 genome sequence. The N50 (951.7 kb) and N90 (246.2 kb) are colored in yellow.

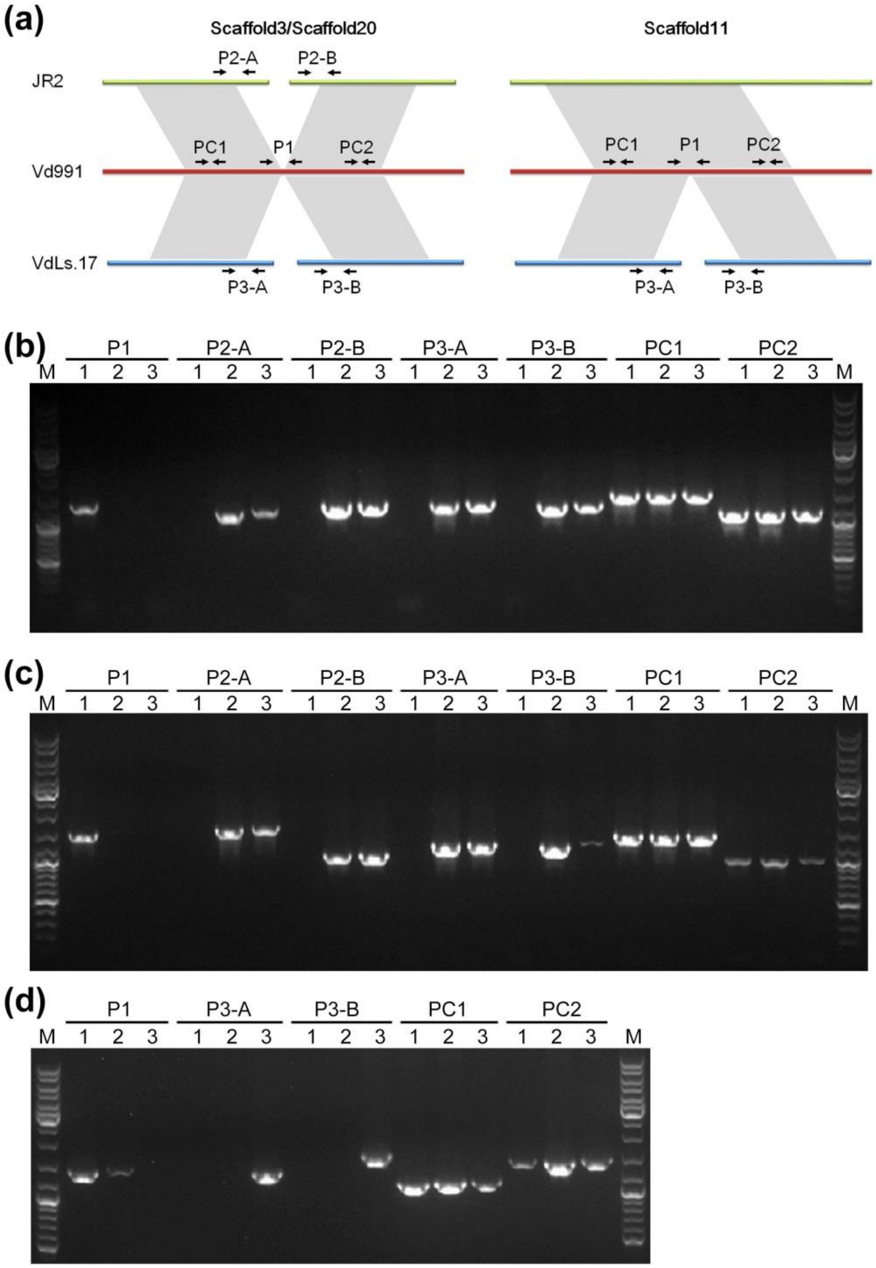

Fig. S3 Verification of rearrangements sites among the three *Verticillium dahliae* strains (Vd991, JR2 and VdLs.17) by PCR. (a) Schematic representation of the PCR targets for the three rearrangements of Scaffold3, Scaffold20 and Scaffold11 in Vd991 compared to the JR2 and VdLs.17 genomes. **(b-d)**, PCR amplification of the structural rearrangements sites in Scaffold3 (**a**), Scaffold20 (**b**) and Scaffold11 (**c**) among the three strains. Numbers 1, 2 and 3 represent the samples Vd991, JR2 and VdLs17, respectively; M, 1 kb DNA ladder.

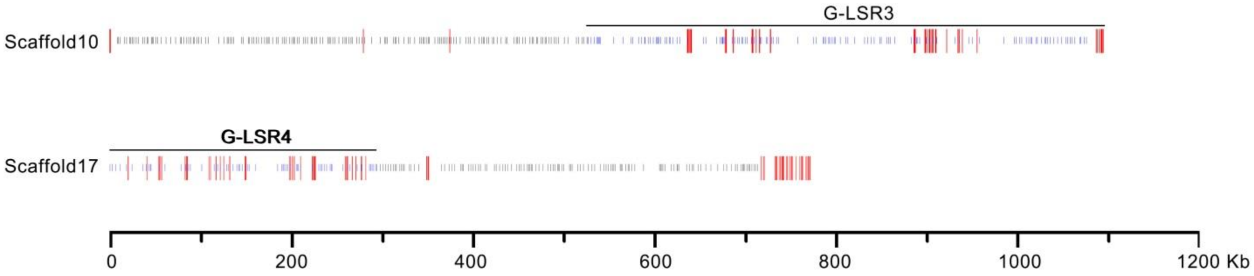

Fig. S4 The distribution of transposable elements (TEs) in the lineage specific regions LSRs in the genome of *Verticillium dahliae* Vd991. G-LSR3 and G-LSR4 are two LSRs in the scaffolds of the Vd991 genome; genes within LSRs are marked in blue and red lines represent the TEs.

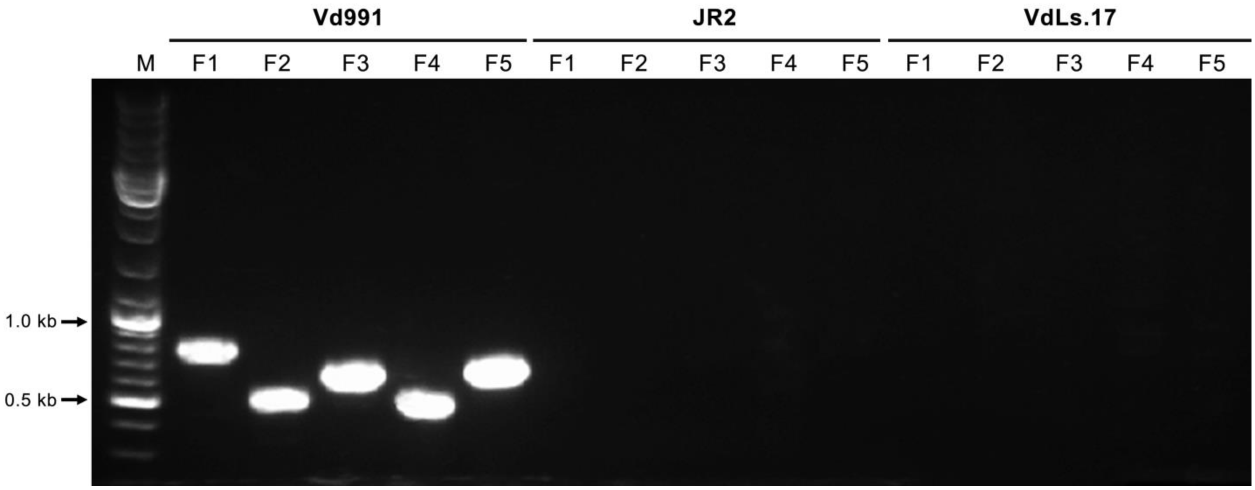

Fig. S5 PCR verification of the lineage specific (LS) genes in putative *Gossypium* lineage specific region G-LSR2 compared to the isolates JR2 and VdLs.17. F1 to F5 represents five arbitrary fragments in G-LSR2; M, 1 kb DNA ladder.

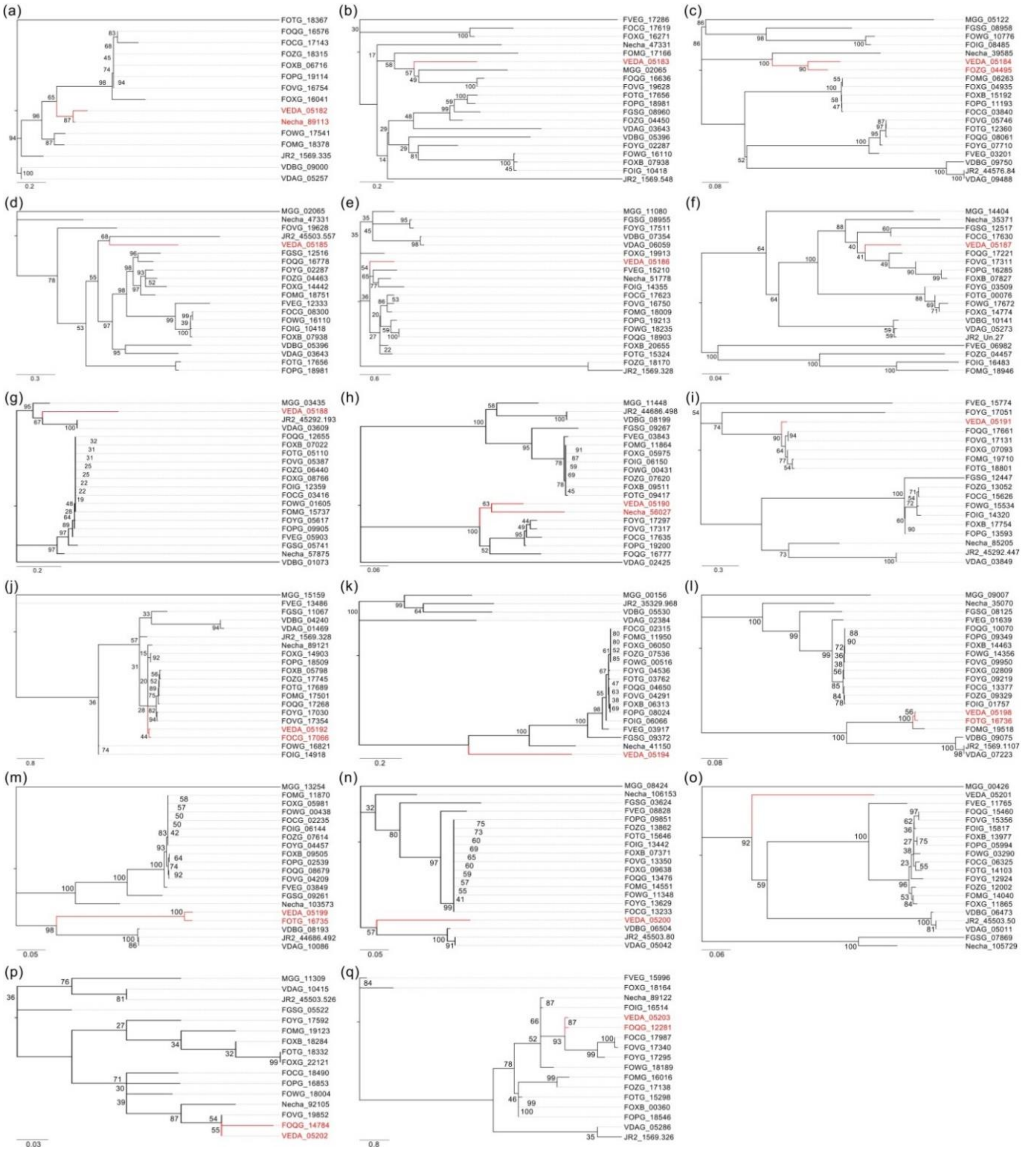

Fig. S6 Phylogenetic relationships between Vd991 lineage specific (LS) genes within putative *Gossypium* lineage specific region G-LSR2 and orthologous genes from *Fusarium* spp. Phylogenetic analyses were performed using nucleic acid sequences of genes in G-LSR2 and the maximum-likelihood method (1,000 bootstraps). (**a-q**), phylogenetic trees of 17 protein-coding genes in G-LSR2. The branches including genes from G-LSR2 and *Fusarium* spp. are drawn in red.

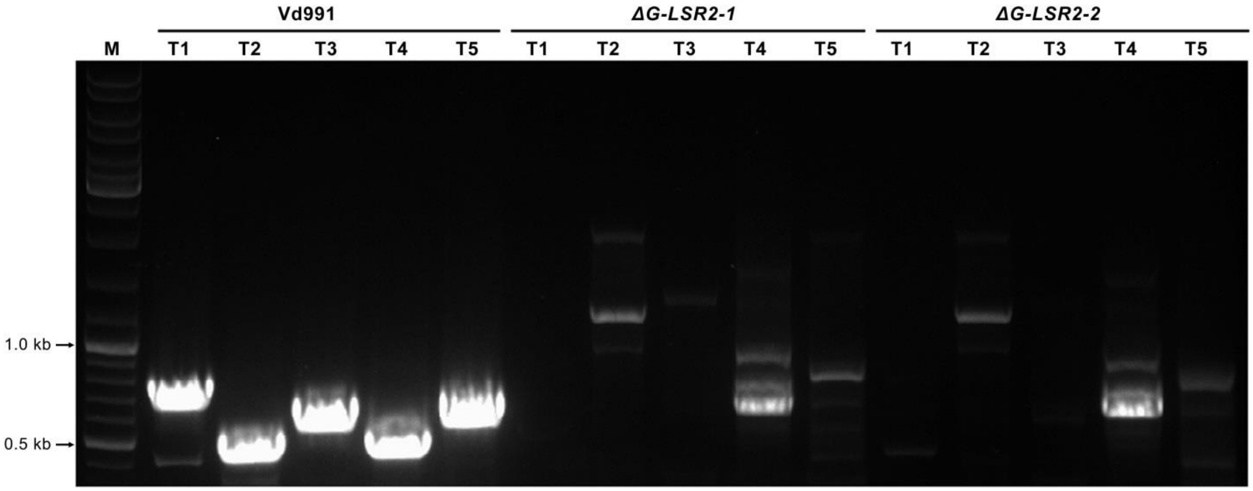

Fig. S7 Validation of the targeted deletions of G-LSR2 in *Verticillium dahliae* Vd991 by PCR. The targeted deletion strains cannot amplify the five detecting fragments (T1 to T5 in G-LSR2) compared with wide-type of Vd991. Δ*G-LSR2-1* and Δ*G-LSR2-1* represents two independent of G-LSR2 deletion strains; M, 1 kb DNA ladder.

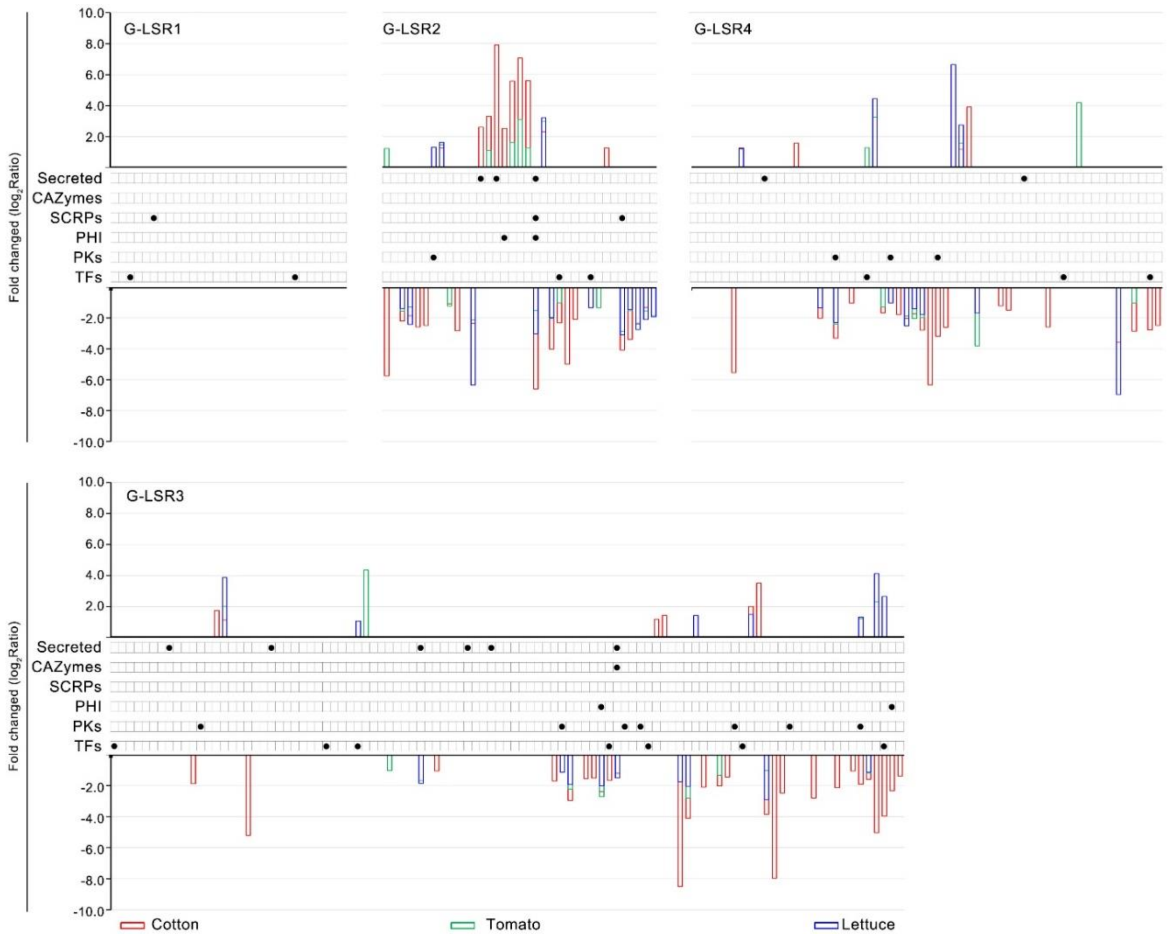

Fig. S8 Gene expression patterns of genes harbored in the four putative *Gossypium* lineage specific regions (G-LSRs 1-4) of the genome of *Verticillium dahliae* Vd991 24 hours after inoculation on cotton, tomato and lettuce roots. 12 four-week old seedlings of cotton (*G. hirsutum* var. Junmian 1), tomato (*Solanum lycopersicum* var. MoneyMaker), and lettuce (*Lactuca sativa* var. *capitata* L.) roots were washed and soaked in a *V. dahliae* Vd991 condial suspension (2×10^9 conidia/ml) and then incubated at 21° C in a sterile glass box for 24 hours. Conidia were then collected from the rootswith 50 mL of sterile distilled water. The collected conidial were centrifuged and for collection. The conidial suspension from a PDA plate of Vd991 was harvested as a control sample. One experiment was performed consisting of four *V. dahliae* libraries (three infected samples on host plants and one control were generated. The red, green and blue columns represent the transcription value (log_2Ratio) of genes encoded in putative lineage specific regions (G-LSRs) and represent that fungal transcription

responses to cotton, tomato and lettuce, respectively. The genes encoding pathogenicity-related factors in the G-LSRs are marked with black dots.

Table S1. Primers used in the study.

Primers name	Sequence (5'-3')
Rearrangement site in Scaffold3	
S3-P1-F	GACACTCATTTACCTCACAACAAC
S3-P1-R	AGCGACGGACAGACAGTATC
S3-P2A-F	ACTCATTTACCTCACAACAACTAAC
S3-P2A-R	GGACTGACAAGATCGGGAATC
S3-P2B-F	AGATATTGCTGCCTGGATATTGAC
S3-P2B-R	ATGACGCTGATGACAAGTTGAC
S3-P3A-F	ACCTCACAACAACTAACCCAAAG
S3-P3A-R	TGCCGAAGAAGGACTGACAG
S3-P3B-F	ATGACGCTGATGACAAGTTGAC
S3-P3B-R	AGATATTGCTGCCTGGATATTGAC
S3-PC1-F	AAGCAGTCGGCAGAGAAGAG
S3-PC1-R	ATGAGCGTTGGTGTATGTCTAATC
S3-PC2-F	AGACGGTAGTTGACGGAGAAG
S3-PC2-R	CCTTGCTGCCTCTGGAATTG
Rearrangement site in Scaffold20	
S20-P1-F	TCCTCTTCTCCACGACCCA
S20-P1-R	CATCTCCTCCCGCATCAAC
S20-P2A-F	TTGATCCGAACCGTCTCCTC
S20-P2A-R	TGTTGCGTACTTCTCCTGATTG
S20-P2B-F	ACTCATTTACCTCACAACAACTAAC
S20-P2B-R	GGACTGACAAGATCGGGAATC
S20-P3A-F	ATGACGCTGATGACAAGTTGAC
S20-P3A-R	AGATATTGCTGCCTGGATATTGAC
S20-P3B-F	ACCTCACAACAACTAACCCAAAG
S20-P3B-R	TGCCGAAGAAGGACTGACAG
S20-PC1-F	GCATCTGTGGCTGAGTTGTG
S20-PC1-R	ACGGCAGTGGCATCATTATC
S20-PC2-F	AGGCGGATGAGTGGTTGTG
S20-PC2-R	TCAAGGTGGCAGCAGAAGAG
Rearrangement site in Scaffold11	
S11-P1-F	TAAGTTGGTGTACGAGATCAGTC
S11-P1-R	GAGGTTGTGAGGAAGGATGTC
S11-P3A-F	CACAATTTCTCCAGCACACTAAAG
S11-P3A-R	TCGTGGTTCAGATGGTCAGAG
S11-P3B-F	ACTGTGTTGAGGATGTATTTCTGG
S11-P3B-R	GAGGTTGTGAGGAAGGATGTTG
S11-PC1-F	GCTTCATTGCTCGTTTGGAAATC
S11-PC1-R	AGTCTGTCATCTGGTCGTAGTATG
S11-PC2-F	CCATCAAGTTCAATCAATCCAATTAG
S11-PC2-R	TCCATCGTCATCCTGTCCTAC
For the G-LSR2 detecting among three *V. dahliae* strains	

G-LSR2-F1-F	AGTGAGTCAGGCAACGAAAT
G-LSR2-F1-R	AGAAGTCAGCGGAAATAGGA
G-LSR2-F2-F	GCCTCAGCAAGCCCACCCAT
G-LSR2-F2-R	ACAGTAACGCCGCCGTATTT
G-LSR2-F3-F	TTGAATCATGCGGGTTGTCC
G-LSR2-F3-R	CCTTGCCGACCTTGAGTTGC
G-LSR2-F4-F	CTTACTAATGCCACCAGGAACA
G-LSR2-F4-R	AACAGGAAGCAGACCAACCC
G-LSR2-F5-F	CAACCCAGCGGACCCTACCT
G-LSR2-F5-R	CCAACACGGATGGACAGAGC

For targeted deletion of the seven genes in G-LSR2

G-LSR2-P1-F	TCTTCTGGGTAGACCATCTCA
G-LSR2-P3-R	GCCCAAAAATGCTCCTTCAACCTCTTACACCTAACCTTTCG
G-LSR2-P4-F	CCCTGGGTTCGCAAAGATAACTTTCGGGATAAGTGCCTAG
G-LSR2-P6-R	TGGAATCTTACGAAGGGTGT
G-LSR2-P2-F	GGGGACAAGTTTGTACAAAAAAGCAGGCTATTACGCAGCATGTCGGAGGG
G-LSR2-P5-R	GGGGACCACTTTGTACAAGAAAGCTGGGTAAGGTCGCAGCCGGTATTGA
Hyg-F	TTGAAGGAGCATTTTTGGGC
Hyg-R	TTATCTTTGCGAACCCAGGG

For the validation of seven genes targeted deletion strain

G-LSR2-T1-F	AGTGAGTCAGGCAACGAAAT
G-LSR2-T1-R	AGAAGTCAGCGGAAATAGGA
G-LSR2-T2-F	GCCTCAGCAAGCCCACCCAT
G-LSR2-T2-R	ACAGTAACGCCGCCGTATTT
G-LSR2-T3-F	TTGAATCATGCGGGTTGTCC
G-LSR2-T3-R	CCTTGCCGACCTTGAGTTGC
G-LSR2-T4-F	CTTACTAATGCCACCAGGAACA
G-LSR2-T4-R	AACAGGAAGCAGACCAACCC
G-LSR2-T5-F	CAACCCAGCGGACCCTACCT
G-LSR2-T5-R	CCAACACGGATGGACAGAGC

For introduce the LS genes of G-LSR2 to JR2 and VdLs.17

VEDA_05193-F	CCGGAATTCCAGCCGATATCATGACGT
VEDA_05193-R	CGCGGATCCTTAATTGGCCATTTCTTT
VEDA_05194-F	CCGGAATTCTCTGCTGACGCGGCTTCC
VEDA_05194-R	CGCGGATCCTTGAGGTTGACCTTCTCT
VEDA_05195-F	CCGGAATTCGCACTCGTAATATTCGCC
VEDA_05195-R	CCCCCCGGGGCAATGAACAGGTCAAGG
VEDA_05196-F	CCGGAATTCAGGATAACGTAGTCGATG
VEDA_05196-R	CGCGGATCCCCACACATGACCTCGGGT
VEDA_05197-F	CCGGAATTCCAGCGATGGCCCTGCCGA
VEDA_05197-R	CGCGGATCCAGCAGCCATTCTCGCGCT
VEDA_05198-F	CGCGGATCCATATACAAGTTTGGTTAC
VEDA_05198-R	TGCTCTAGATGGGGCCATTCATCCCAG
VEDA_05199-F	CGCGGATCCCGACACACAAAAGGGCCT
VEDA_05199-R	TGCTCTAGATATTGAGGATATCTAGGA

For fungal biomass detection	
Vd-EF-1α-F	TGAGTTCGAGGCTGGTATCT
Vd-EF-1α-R	CACTTGGTGGTGTCCATCTT
Gh-18S-F	CGGCTACCACATCCAAGGAA
Gh-18S-R	TGTCACTACCTCCCCGTGTCA

Table S2. Raw data of genome sequencing by PacBio RS II and Illumina technologies.

**Data type**		**PacBio RS II**	**Illumina MiSeq (PE)**	**Illumina HiSeq (PE)**
Insert size (bp)		10,000	400	10,000
	Read counts	524,931	11,819,618	13,234,057
	Read length (bp)	-	250	100
	Total bases (bp)	2,263,648,017	2,954,904,500	2,646,811,400
	Read counts	460,854	11,389,280	13,680,620
	Read length (bp)	-	250	100
	Total bases (bp)	2,227,210,130	2,474,046,912	1,368,062,000

Table S3. Key parameters of the genome assembly of the *Verticillium dahliae* strain Vd991by PacBio RS II biotechnology.

Parameter	Contigs
Total number	257
Total length of (bp)	33273465
Gap number (bp)	0
Average length (bp)	129468
N50 length (bp)	556844
N90 length (bp)	113768
Maximum length (bp)	1888474
Minimum length (bp)	258
GC content (%)	55.75

Table S4. Improvement in genome sequence quality of *Verticillium dahliae* strain Vd991 with Illumina MiSeq data.

**Parameter**	**Contigs**
Total number	476
Total length of (bp)	34256470
Gap number (bp)	0
Average length (bp)	70924
N50 length (bp)	599791
N90 length (bp)	97469
Maximum length (bp)	1934785
Minimum length (bp)	289
GC content (%)	55.02

Table S5. Key parameters of the genome assembly of the Vd991 strain.

Parameter	Scaffolds	Contigs
Total number	165	382
Total length of (bp)	34759399	34551100
Gap number (bp)	208299	0
Average length (bp)	210663	90448
N50 length (bp)	951741	630613
N90 length (bp)	246165	98963
Maximum length (bp)	4099674	1938240
Minimum length (bp)	568	200
GC content (%)	54.8	54.8

Table S6. Physical locations of *Verticillium dahliae* strain Vd991 genomic regions relative to reference genomes of V. dahliae JR2 and VdLs.17.

		Vd991			JR2				VdLs.17			
		**Start**	**End**	**Length**	**Start**	**End**	**Length**	**Chr**	**Start**	**End**	**Length**	**Chr**
Scaffold29	394153	2145	370324	368180	17215	384858	367644	1	5518808	5887373	368566	1
Scaffold53	78450	15146	74995	59850	403557	463434	59878	1	5438318	5498613	60296	1
**Scaffold7**	1236054	11252	1106150	1094899	594125	1708278	1114154	1	4186567	5302124	1115558	1
Scaffold35	252238	8368	251460	243093	3942082	4186575	244494	1	1708270	1953367	245098	1
Scaffold12	889730	77464	881074	803611	1953362	2758884	805523	1	3138738	3942087	803350	1
Scaffold12	889730	140	77462	77323	2758885	2835587	76703	1	5085070	5161708	76639	2
Scaffold19	642043	387	635263	634877	3002195	3639806	637612	1	4326463	4961784	635322	2
**Scaffold1**	4099674	72729	4099674	4026946	3681150	7899230	4218081	1	58977	4299631	4240655	2
**Scaffold18**	769016	10038	341838	331801	7899231	8230835	331605	1	939450	1284296	344847	3
Scaffold38	213455	9475	206602	197128	8230828	8434463	203636	1	742307	939457	197151	3
Scaffold42	158061	749	150953	150205	8441127	8590773	149647	1	578737	727919	149183	3
Scaffold48	111714	11582	69083	57502	8590771	8646551	55781	1	510647	565486	54840	3
Scaffold32	270261	15314	269821	254508	8653092	8915283	262192	1	246709	510646	263938	3
**Scaffold7**	1236054	1126182	1235141	108960	8915275	9024326	109052	1	137678	246717	109040	3
**Scaffold44**	145608	18644	131074	112431	9024177	9143047	118871	1	16273	137830	121558	3
Scaffold33	266435	3444	256498	253055	26	231560	231535	2	3041276	3272756	231481	7
Scaffold34	266139	15	263935	263921	231560	499860	268301	2	802	269904	269103	7
Scaffold25	549517	25528	536780	511253	654475	1167068	512594	2	2124261	2634645	510385	7
**Scaffold3**	1691980	1297928	1685432	387505	1168025	1565608	397584	2	1742993	2123288	380296	7

**Scaffold20**	627608	20029	469617	449589	1565609	2017032	451424	2	1292808	1742992	450185	7
Scaffold39	205420	6021	199099	193079	2017027	2192839	175813	2	1117464	1292813	175350	7
Scaffold22	613111	4524	608573	604050	2192835	2803066	610232	2	507189	1117468	610280	7
**Scaffold40**	177890	817	65027	64211	2803066	2867351	64286	2	442982	507189	64208	7
**Scaffold40**	177890	76276	164133	87858	3028888	3116199	87312	2	2193127	2281265	88139	3
**Scaffold17**	770001	186911	199715	12805	3270546	3283348	12803	2	/	/	/	/
**Scaffold10**	1096014	777901	800675	22775	3320252	3348597	28346	2	3660908	3683745	22838	4
Scaffold45	123429	6232	118636	112405	3660828	3773640	112813	2	2037338	2151911	114574	3
**Scaffold18**	769016	341834	747519	405686	3809901	4215853	405953	2	1284292	1690468	406177	3
**Scaffold1**	4099674	53383	72732	19350	4215852	4233942	18091	2	40960	58979	18020	2
Scaffold26	463925	26005	429115	403111	14473	418586	404114	3	5788281	6209716	421436	2
Scaffold30	393782	527	390669	390143	420442	819525	399084	3	5387626	5778356	390731	2
Scaffold11	951741	151364	371508	220145	819521	1040524	221004	3	5167581	5387630	220050	2
Scaffold11	951741	371504	942823	571320	1040520	1632084	591565	3	2543583	3132864	589282	1
Scaffold21	619576	6377	612742	606366	1793865	2403445	609581	3	1766414	2373234	606821	1
Scaffold6	1464317	10238	1463244	1453007	2441672	3904892	1463221	3	268984	1728780	1459797	1
**Scaffold41**	169014	45396	168225	122830	4033702	4156785	123084	3	2326	125278	122953	1
**Scaffold24**	555505	529	420774	420246	2975	406519	403545	4	346	402850	402505	5
**Scaffold23**	606739	69480	580082	510603	406509	932096	525588	4	402840	937108	534269	5
**Scaffold10**	1096014	718224	749970	31747	1278911	1311088	32178	4	3554931	3587368	32438	4
**Scaffold10**	1096014	635998	651374	15377	1312951	1328327	15377	4	3482710	3544091	61382	4
**Scaffold23**	606739	3610	69487	65878	1448084	1513838	65755	4	937101	1002656	65556	5

Scaffold4	1469601	11395	1458727	1447333	1516954	2854026	1337073	4	1005771	2349818	1344048	5
**Scaffold5**	1466777	537125	1466708	929584	3027801	3938573	910773	4	2541564	3442910	901347	5
Scaffold50	87113	6507	86988	80482	3945983	4026214	80232	4	3482669	3571761	89093	5
**Scaffold54**	73901	23854	52055	28202	23	27640	27618	5	4280420	4308036	27617	4
**Scaffold10**	1096014	6095	508270	502176	34209	538425	504217	5	3733668	4274247	540580	4
**Scaffold10**	1096014	836719	826237	10483	639190	649674	10485	5	/	/	/	/
**Scaffold54**	73901	52054	71296	19243	916603	935844	19242	5	4308035	4327276	19242	4
Scaffold13	861191	53068	848955	795888	1015307	1817508	802202	5	2232314	3050483	818170	4
**Scaffold24**	555505	427374	545226	117853	1698966	1817109	118144	5	2233438	2351268	117831	4
Scaffold36	246165	3947	102136	98190	1973317	2072577	99261	5	1972887	2071874	98988	4
Scaffold36	246165	105222	245402	140181	2128862	2270015	141154	5	1755173	1895475	140303	4
**Scaffold5**	1466777	12781	527011	514231	2307210	2819252	512043	5	1134860	1646406	511547	4
**Scaffold17**	770001	284528	714421	429894	2819557	3242036	422480	5	706976	1128640	421665	4
**Scaffold10**	1096014	995863	1080184	84322	3242192	3326586	84395	5	617160	706820	89661	4
Scaffold27	452810	9095	451572	442478	3643048	4065492	422445	5	4676	426962	422287	7
Scaffold15	831454	12166	831147	818982	1486	846536	845051	6	5007327	5882771	875445	3
Scaffold3	1691980	30576	297119	266544	846532	1082705	236174	6	4771131	5006654	235524	3
**Scaffold3**	1691980	334027	1297929	963903	1082700	2005559	922860	6	3850693	4771136	920444	3
**Scaffold20**	627608	469765	619011	149247	2005733	2154850	149118	6	3695520	3850693	155174	3
Scaffold16	827563	7139	821149	814011	2309063	3155482	846420	6	2695328	3545712	850385	3
Scaffold31	280543	242287	269138	26852	3158365	3185246	26882	6	2665822	2692709	26888	3

Scaffold31	280543	11259	242287	231029	3192377	3410149	217773	6	89179	307573	218395	4
**Scaffold8**	1177875	20	1016591	1016572	327847	1346073	1018227	7	230733	1241252	1010520	8
Scaffold2	1940918	8031	1929679	1921649	1430516	3361777	1931262	7	1339357	3290788	1951432	8
Scaffold28	440952	14952	435704	420753	1	415291	415291	8	2962359	3402483	440125	6
Scaffold37	213648	14078	195123	181046	468337	658558	190222	8	2740507	2933922	193416	6
Scaffold9	1133803	11184	1133803	1122620	671026	1784547	1113522	8	1599998	2727588	1127591	6
Scaffold49	108471	14389	108471	94083	1926000	2020176	94177	8	1343821	1437844	94024	6
Scaffold43	154559	227	150196	149970	2079884	2230183	150300	8	1157120	1308975	151856	6
Scaffold55	70805	8528	64949	56422	2352618	2409148	56531	8	964607	1021103	56497	6
Scaffold14	841791	8170	836313	828144	147708	964611	816904	8	2409144	3220071	810928	6
Scaffold46	118562	47	108571	108525	3220067	3351732	131666	8	17532	147712	130181	6
**Scaffold10**	1096014	587989	604110	16122	123568	139680	16113	Un	3271974	3288146	16173	4
**Scaffold17**	770001	87805	154270	66466	437815	504653	66839	Un	3138418	3234742	96325	4
Scaffold58	37212	1	11265	11265	583440	594704	11265	Un	2292	21815	19524	4
**Scaffold44**	145608	132642	145086	12445	908629	921049	12421	Un	2285	14705	12421	3
**Scaffold10**	1096014	570661	587928	17268	1192782	1210041	17260	Un	3288205	3305467	17263	4
**Scaffold1**	4099674	1051	20761	19711	1263221	1282925	19705	Un	9825	30359	20535	2
**Scaffold41**	169014	3154	45400	42247	1496267	1538553	42287	Un	176295	218537	42243	1
**Scaffold8**	1177875	1016808	1168019	151212	1553891	1705789	151899	Un	91	160819	160729	8
Scaffold47	118290	1	15739	15739	/	/	/	/	5969299	5985136	15838	1
Scaffold47	118290	20927	45654	24728	/	/	/	/	5960789	5985621	24833	1
Scaffold47	118290	31126	85748	54623	/	/	/	/	5941463	5959055	17593	1

Scaffold59	29388	12	17665	17654	/	/	/	/	5972032	5989820	17789	1
Scaffold17	770001	18262	3871	14392	/	/	/	/	3305108	3319527	14420	4
Scaffold17	770001	31871	56160	24290	3692791	3722564	29774	Un	3692791	3722564	29774	4
Scaffold17	770001	229108	239193	10086	/	/	/	/	3741437	3751658	10222	4
Scaffold17	770001	239196	262251	23056	3518114	3541168	23055	Un	3518114	3541168	23055	4
**Total length of core genome**	**32051825**				**32030818**				**32364238**			

Note: the length of alignment block cut off >10 kb. Scaffolds marked in red color were aligned to different physical location in JR2 or VdLs.17 genome. The Scaffolds were ordered by the chromosome number in JR2 genome from top to bottom. Un, represents the physical location of assemble sequences cannot map to the chromosome in JR2 genome.

Table S7. Location of rearrangements in Vd991 genome compared to JR2 and VdLs.17 genomes.

	Vd991					JR2				VdLs.17			
	Scaffold	Breakpoints	Start	End	Length	Chr	Start	End		Chr	Start	End	
			334027	1297929	963903	6	1082700	2005559		3	4771136	3850693	
			1297928	1685432	387505	2	1565608	1168025		7	1742993	2123288	
			12781	527011	514231	5	2307210	2819252		4	1646406	1134860	
			537125	1466708	929584	4	3027801	3938573		5	2541564	3442910	
			11252	1106150	1094899	1	594125	1708278		1	5302124	4186567	
			1126182	1235141	108960	1	8915275	9024326		3	246717	137678	
			151364	371508	220145	3	819521	1040524		2	5387630	5167581	
			371504	942823	571320	3	1040520	1632084		1	3132864	2543583	
			140	77462	77323	1	2835587	2758885		2	5085070	5161708	
			77464	881074	803611	1	2758884	1953362		1	3138738	3942087	
			10038	341838	331801	1	8230835	7899231		3	939450	1284296	
			341834	747519	405686	2	4215853	3809901		3	1284292	1690468	
			20029	469617	449589	2	2017032	1565609		7	1292808	1742992	
			469765	619011	149247	6	2005733	2154850		3	3850693	3695520	
			529	420774	420246	4	2975	406519		5	346	402850	
			427374	545226	117853	5	1698966	1817109		4	2351268	2233438	
			11259	242286	231029	6	3410149	3192377		4	89179	307573	
			242287	269138	26852	6	3185246	3158365		3	2665822	2692709	
			817	65027	64211	2	2803066	2867351		7	507189	442982	
			76276	164133	87858	2	3028888	3116199		3	2281265	2193127	
			3610	69487	65878	4	1513838	1448084		5	1002656	937101	
			69480	580082	510603	4	932096	406509		5	937108	402840	
			3947	102136	98190	5	1973317	2072577		4	2071874	1972887	
			105222	245402	140181	5	2128862	2270015		4	1755173	1895475	

* Re-arrangement (RA) compared to Vd991. IE, inter-chromosomal rearrangement (occurs in different chromosomes); IA, intra-chromosomal rearrangement (occurs in the same chromosomes); Syn, synteny

Table S8. Comparison of gene models among the three *V. dahliae* Vd991, JR2 and VdLs.17 genomes

**Parameter**	**Vd991 (this study)**	**JR2[*]**	**VdLs.17[#]**
Host origin	cotton	tomato	lettuce
Genome size (bp)	34759399	36213039	33828453
Protein coding genes	9818	10985	10535
Mean gene length (bp)	1643.51	1538.18	1,615.24
Coding gene with introns	2453 (25.0%)	2913 (26.5%)	2204 (20.9%)
Mean exons per gene	2.67	2.71	2.81
Mean exons length (bp)	554.14	511.53	509.85
Mean intron length (bp)	98.97	88.56	99.69
Mean intergenic length (bp)	1853.03	1875.86	1632.1
GC content (%)	54.8	55.46	55.85%

*The JR2 genome assembly published by de Jonge et al. (de Jonge R, Bolton MD, Kombrink A, van den Berg GC, Yadeta KA, Thomma BP. 2013. Extensive chromosomal reshuffling drives evolution of virulence in an asexual pathogen. Genome Research 23:1271–1282); [#]The VdLs.17 genome assembly published by Klosterman et al. (Klosterman SJ, Subbarao KV, Kang S, Veronese P, Gold SE, Thomma BP, Chen Z, Henrissat B, Lee YH, Park J et al. 2011. Comparative genomics yields insights into niche adaptation of plant vascular wilt pathogens. PLOS Pathogens 7:e1002137).

Table S10. Analysis and comparisons of specific genes among the three *V. dahliae* Vd991, JR2 and VdLs.17 genomes.

Parameter	Vd991	JR2	VdLs.17
Protein coding genes	9818	10985	10535
Genes unable to be clustered into orthologues	506	1296	1026
Identities or coverage ratio <50%	737	1560	1288
Without synteny and multi-copies	175	62	71
**Combined to calling specific genes**	**960**[*]	**1652**[&]	**1390**[$]
**Common genes (Synteny genes)**[#]		**8049**	

[*]Vd991 *vs* JR2 and VdLs.17; [&]JR2 *vs* Vd991 and VdLs.17; [$]VdLs.17 *vs* Vd991 and JR2; [#]common genes among Vd991, VdLs.17 and JR2.

Table S11. Fungi non-supervised orthologous groups (fuNOG) annotation of encoding genes among the three *V. dahliae* Vd991, JR2 and VdLs.17 genomes

		Genome			Common genes			Specific genes		
		**Vd991**	**JR2**	**VdLs.17**	**Vd991**	**JR2**	**VdLs.17**	**Vd991**	**JR2**	**VdLs.17**
D	Cell cycle control, cell division, chromosome partitioning	112	111	110	94	94	94	8	2	8
M	Cell wall/membrane/envelope biogenesis	23	24	25	20	21	21	2	1	2
N	Cell motility	2	2	2	2	2	2	0	0	0
O	Posttranslational modification, protein turnover, chaperones	442	444	448	398	399	399	18	13	21
T	Signal transduction mechanisms	207	211	219	178	178	179	11	5	24
U	Intracellular trafficking, secretion, and vesicular transport	308	305	310	277	277	277	9	6	14
V	Defense mechanisms	34	34	33	29	30	30	5	0	0
W	Extracellular structures	2	2	2	2	2	2	0	0	0
Y	Nuclear structure	3	3	3	3	3	3	0	0	0
Z	Cytoskeleton	91	89	88	78	78	78	8	4	5
A	RNA processing and modification	271	276	267	241	241	240	8	13	9
B	Chromatin structure and dynamics	73	72	71	62	61	61	6	4	5
J	Translation, ribosomal structure and biogenesis	353	358	359	311	312	312	11	16	16
K	Transcription	429	450	474	357	355	360	26	24	47
L	Replication, recombination and repair	199	206	202	183	183	183	6	4	6
C	Energy production and conversion	312	306	308	278	278	278	17	5	12
E	Amino acid transport and metabolism	264	268	270	244	245	244	6	4	17

F	Nucleotide transport and metabolism	81	80	81	74	73	73	3	2	3
G	Carbohydrate transport and metabolism	564	578	578	506	504	504	28	20	34
H	Coenzyme transport and metabolism	91	86	87	77	75	77	8	3	4
I	Lipid transport and metabolism	263	267	268	234	232	233	12	11	19
P	Inorganic ion transport and metabolism	211	222	229	188	188	188	9	7	22
Q	Secondary metabolites biosynthesis, transport and catabolism	299	318	320	261	262	261	17	18	27
R	General function prediction only	684	705	694	585	585	584	39	35	45
S	Function unknown	2438	2516	2522	2061	2069	2070	180	132	180

Note: the annotation of fuNOG using by eggNOG v3.0, http://eggnog.embl.de/version_3.0/

Table S12. Functional annotation of potential pathogenicity and virulence-related factors among the three *Verticillium dahliae* strains Vd991, JR2 and VdLs.17 genomes.

Types	Vd991	JR2	VdLs.17
Secretome	**739 (7.53%)**	767 (6.98%)	749 (7.11%)
SCRPs	**127 (1.29%)**	115 (1.05%)	123 (1.17%)
CAZymes	**605 (6.16%)**	621 (5.65%)	617 (5.86%)
PHI proteins	**268 (2.73%)**	266 (2.42%)	258 (2.45%)
PKs	**133 (1.35%)**	131 (1.19%)	145 (1.38%)
TFs	**452 (4.60%)**	503 (4.58%)	489 (4.64%)

Note: Secretome, secreted protein predicted with the intergated four programs of WoLF PSORT (Horton P, Park KJ, Obayashi T, Fujita N, Harada H, Adams-Collier CJ, Nakai K. 2007. WoLF PSORT: protein localization predictor. Nucleic Acids Res. 35(Web Server issue):W585-587), SignalP (Version 4.1, Nielsen H. 2017. Predicting Secretory Proteins with SignalP. Methods Mol Biol. 1611:59-73), Phobius (Kall L, Krogh A, Sonnhammer ELL. 2007. Advantages of combined transmembrane topology and signal peptide prediction–the Phobius web server. Nucl Acids Res 35: W429-432), and TMHMM (Version 2.0, Krogh A, Larsson B, von Heijne G, Sonnhammer ELL. 2001. Predicting transmembrane protein topology with a hidden Markov model: Application to complete genomes. J Mol Biol 305:567-580); SCRPs, small cysteine-rich proteins; the secreted protein filtered with <400 amino acids, ≥4 cysteine residues; CAZymes, carbohydrate-active enzymes; performed using HMM-based routine of the Carbohydrate-Active-EnZymes database (http://www.cazy. org/, Cantarel BL, Coutinho PM, Rancurel C, Bernard T, Lombard V, Henrissat B. 2009. The Carbohydrate-Active enZymes database (CAZy): an expert resource for glycogenomics. Nucleic Acids Res 37: D233-D238) and BLASTP (Altschul SF, Madden TL, Schaffer AA, Zhang J, Zhang Z, Miller W, Lipman DJ. 1997. Gapped BLAST and PSI-BLAST: a new generation of protein database search programs. Nucleic Acids Research 25:3389–3402); PHI, pathogen-host interaction proteins; annotation with the PHI-database (Version 3.6, http://www.phi-base.org/, Winnenburg, R., Urban, M., Beacham, A., Baldwin, T.K., Holland, S., Lindeberg, M., Hansen, H., Rawlings, C., Hammond-Kosack, K.E., and Kohler, J. 2008. PHI-base update: additions to the pathogen host interaction database. Nucleic Acids Res 36:D572-576); PKs, protein kinases; annotation with Kinomer (Version 1.0, Martin DM, Miranda-Saavedra D, Barton GJ. Kinomer v. 1.0: a database of systematically classified eukaryotic protein kinases. Nucleic Acids Res. 2009 Jan;37(Database issue):D244-250); TFs, transcription factors; annotation using the domain analysis using InterProScan 5 (Jones P , Binns D, Chang HY, Fraser M, Li W, McAnulla C, McWilliam H, Maslen J, Mitchell A, Nuka G, Pesseat S, Quinn AF, Sangrador-Vegas A, Scheremetjew M, Yong SY, Lopez R, Hunter S. 2014. InterProScan 5: genome-scale protein function classification. Bioinformatics. 30:1236-1240).

Table S13. Classification of the sub-families of CAZymes in the genomes of the three *Verticillium dahliae* isolates Vd991, JR2 and VdLs.17.

	Total genes			Common genes			Specific genes		
	Vd991	JR2	VdLs.17	Vd991	JR2	VdLs.17	Vd991	JR2	VdLs.17
AA1	2(1)	3(1)	3(1)	2(1)	3(1)	3(1)	0	0	0
AA2	8(5)	9(6)	8(5)	8(5)	8(5)	8(5)	0	1(1)	0
AA3	23(10)	24(10)	23(8)	22(9)	22(9)	21(7)	0	1(1)	0
AA4	2(0)	1(0)	0	2(0)	1(0)	0	0	0	0
AA5	2(2)	2(2)	2(1)	2(2)	2(2)	2(1)	0	0	0
AA6	1(0)	1(0)	1(0)	1(0)	1(0)	1(0)	0	0	0
AA7	26(17)	29(18)	29(19)	24(17)	25(18)	25(19)	1(0)	0	0
AA8	3(2)	3(1)	2(2)	1(1)	1(1)	1(1)	0	1(0)	0
AA9	25(16)	27(17)	25(19)	21(13)	21(13)	21(16)	1(1)	2(1)	0
AA9, CBM1	3(2)	2(2)	3(1)	2(2)	2(2)	2(1)	0	0	0
CBM1	3(3)	4(2)	5(4)	0	2(2)	2(2)	1(1)	2(0)	1(0)
CBM1, CE15	1(1)	1(1)	1(1)	1(1)	1(1)	1(1)	0	0	0
CBM1, GH5	2(2)	2(2)	2(2)	1(1)	1(1)	1(1)	0	1(1)	0
CBM1, GH5, CBM46	1(1)	0	0	1(1)	0	0	0	0	0
CBM1, GH6	1(1)	1(1)	1(1)	1(1)	1(1)	1(1)	0	0	0
CBM1, PL1	1(1)	2(2)	2(2)	1(1)	1(1)	1(1)	0	0	0
CBM1, PL3	0	0	0	0	0	0	0	0	0
CBM13	1(1)	1(1)	1(1)	1(1)	1(1)	1(1)	0	0	0
CBM18	1(1)	2(2)	3(3)	0	0	0	1(1)	1(1)	2(2)
CBM18, CE4	0	1(1)	1(1)	0	1(1)	1(1)	0	0	0
CBM18, GH16	0	1(1)	1(0)	0	0	0	0	0	0

CBM18, GH18	2(0)	2(0)	1(0)	1(0)	2(0)	1(0)	0	0	0
CBM20	3(1)	3(1)	3(1)	3(1)	3(1)	3(1)	0	0	0
CBM21	1(0)	1(0)	0	0	0	0	0	0	0
CBM35	0	1(1)	1(1)	0	1(1)	1(1)	0	0	0
CBM35, GH26	1(1)	1(1)	1(1)	1(1)	1(1)	1(1)	0	0	0
CBM38, GH32	1(0)	0	1(0)	0	0	0	0	0	0
CBM44	1(0)	1(0)	1(0)	1(0)	1(0)	1(0)	0	0	0
CBM48, GH13	2(0)	1(0)	1(0)	1(0)	1(0)	1(0)	1(0)	0	0
CBM50	4(1)	4(1)	4(1)	3(1)	3(1)	3(1)	0	0	0
CBM50, CBM18	0	1(0)	1(0)	0	0	0	0	0	0
CBM50, CBM18, GH18	0	0	0	0	0	0	0	0	0
CBM63	1(1)	1(1)	1(1)	0	0	0	0	0	0
CBM67	0	0	1(0)	0	0	0	0	0	1(0)
CBM67, GH78	5(0)	5(0)	4(0)	4(0)	4(0)	4(0)	1(0)	1(0)	0
CE1	16(5)	15(6)	16(5)	14(5)	14(6)	15(5)	0	0	0
CE1, CBM1	3(3)	1(1)	2(2)	3(3)	1(1)	2(2)	0	0	0
CE10	40(14)	41(16)	42(17)	34(13)	32(14)	34(15)	5(1)	0	1(0)
CE12	5(4)	5(4)	5(4)	5(4)	5(4)	5(4)	0	0	0
CE14	0	1(0)	1(0)	0	1(0)	1(0)	0	0	0
CE16	3(3)	3(3)	3(3)	3(3)	3(3)	3(3)	0	0	0
CE2	2(2)	1(1)	1(1)	1(1)	1(1)	1(1)	1(1)	0	0
CE3	6(4)	7(5)	7(4)	6(4)	6(4)	5(4)	0	0	1(0)
CE4	8(3)	6(3)	6(3)	7(3)	5(2)	5(2)	1(0)	0	0
CE4, CBM18	1(0)	2(0)	2(0)	1(0)	2(0)	2(0)	0	0	0
CE5	12(11)	12(10)	13(10)	10(9)	10(8)	10(8)	0	1(1)	2(1)

CE5, CBM1	1(1)	1(1)	1(1)	1(1)	1(1)	1(1)	0	0	0
CE7	0	0	1(0)	0	0	1(0)	0	0	0
CE8	5(5)	5(5)	4(4)	3(3)	3(3)	3(3)	1(1)	1(1)	0
CE9	1(0)	1(0)	1(0)	1(0)	1(0)	1(0)	0	0	0
GH1	2(0)	2(0)	2(0)	2(0)	2(0)	2(0)	0	0	0
GH10	3(2)	4(3)	4(3)	3(2)	4(3)	4(3)	0	0	0
GH10, CBM1	1(1)	0	0	1(1)	0	0	0	0	0
GH105	5(3)	5(3)	5(4)	5(3)	5(3)	5(4)	0	0	0
GH106	1(0)	1(0)	1(0)	1(0)	1(0)	1(0)	0	0	0
GH109	14(0)	13(0)	13(0)	11(0)	11(0)	11(0)	0	0	1(0)
GH11	5(4)	4(3)	4(4)	3(3)	3(3)	3(3)	1(1)	0	1(1)
GH11, CBM1	0	1(1)	1(1)	0	1(1)	1(1)	0	0	0
GH114	3(0)	3(1)	3(1)	3(0)	3(1)	3(1)	0	0	0
GH115	3(0)	3(0)	3(0)	3(0)	3(0)	3(0)	0	0	0
GH12	3(0)	5(2)	5(2)	3(0)	4(1)	4(1)	0	0	0
GH12, CBM1	2(2)	1(1)	1(1)	2(2)	1(1)	1(1)	0	0	0
GH120	1(0)	0	1(1)	1(0)	0	1(1)	0	0	0
GH125	1(1)	1(1)	1(1)	1(1)	1(1)	1(1)	0	0	0
GH127	1(1)	1(1)	1(1)	1(1)	1(1)	1(1)	0	0	0
GH128	3(1)	3(1)	4(3)	2(1)	2(1)	2(2)	1(0)	1(0)	2(1)
GH13	6(1)	6(1)	6(1)	6(1)	6(1)	6(1)	0	0	0
GH131	2(2)	1(1)	1(1)	2(2)	1(1)	1(1)	0	0	0
GH131, CBM1	0	1(1)	1(1)	0	1(1)	1(1)	0	0	0
GH132	2(1)	2(1)	2(2)	2(1)	2(1)	2(2)	0	0	0
GH15	1(0)	1(0)	1(0)	1(0)	1(0)	1(0)	0	0	0

GH15, CBM20	3(2)	3(2)	3(2)	3(2)	3(2)	3(2)	0	0	0
GH16	12(4)	11(5)	11(5)	10(4)	10(5)	10(5)	1(0)	0	1(0)
GH17	7(4)	7(4)	7(4)	6(4)	6(4)	6(4)	0	0	1(0)
GH18	12(3)	15(6)	15(5)	11(3)	10(5)	11(4)	1(0)	4(1)	3(1)
GH2	6(1)	6(1)	6(1)	6(1)	6(1)	6(1)	0	0	0
GH20	3(2)	3(2)	3(1)	3(2)	3(2)	3(1)	0	0	0
GH24	1(1)	1(1)	1(1)	1(1)	1(1)	1(1)	0	0	0
GH27	3(3)	4(3)	4(3)	3(3)	4(3)	4(3)	0	0	0
GH28	12(11)	12(10)	12(11)	12(11)	12(10)	12(11)	0	0	0
GH3	17(5)	17(8)	16(7)	15(5)	15(6)	14(5)	1(0)	0	0
GH30	1(1)	1(1)	1(1)	1(1)	1(1)	1(1)	0	0	0
GH31	7(1)	7(1)	6(1)	6(1)	6(1)	6(1)	1(0)	0	0
GH32	2(1)	2(1)	2(1)	2(1)	2(1)	2(1)	0	0	0
GH33	0	1(0)	1(0)	0	0	0	0	0	0
GH35	3(2)	4(3)	4(2)	3(2)	4(3)	4(2)	0	0	0
GH36	1(0)	1(0)	0	0	0	0	0	0	0
GH37	2(1)	2(1)	2(1)	2(1)	2(1)	2(1)	0	0	0
GH38	1(0)	2(0)	2(0)	1(0)	1(0)	1(0)	0	0	0
GH43	17(11)	20(13)	17(12)	17(11)	18(13)	17(12)	0	2(0)	0
GH43, CBM1	0	0	1(0)	0	0	1(0)	0	0	0
GH43, CBM35	1(1)	1(1)	1(1)	1(1)	1(1)	1(1)	0	0	0
GH43, CBM42	1(0)	1(0)	1(1)	1(0)	1(0)	1(1)	0	0	0
GH45	1(1)	1(1)	1(1)	1(1)	1(1)	1(1)	0	0	0
GH45, CBM1	1(1)	1(1)	1(1)	1(1)	1(1)	1(1)	0	0	0
GH47	6(1)	7(1)	7(1)	6(1)	6(1)	6(1)	0	0	0

GH49	1(1)	1(1)	1(1)	1(1)	1(1)	1(1)	0	0	0
GH5	8(4)	9(5)	8(4)	7(3)	8(4)	8(4)	0	0	0
GH5, CBM1	1(1)	1(1)	1(1)	1(1)	1(1)	1(1)	0	0	0
GH51	2(1)	2(1)	2(1)	2(1)	2(1)	2(1)	0	0	0
GH53	1(1)	1(1)	1(1)	1(1)	1(1)	1(1)	0	0	0
GH54, CBM42	1(1)	1(1)	1(1)	1(1)	1(1)	1(1)	0	0	0
GH55	3(3)	3(2)	3(2)	3(3)	3(2)	3(2)	0	0	0
GH6	3(3)	3(3)	3(3)	3(3)	3(3)	3(3)	0	0	0
GH63	1(1)	1(1)	1(1)	1(1)	1(1)	1(1)	0	0	0
GH64	2(1)	2(1)	2(1)	2(1)	2(1)	2(1)	0	0	0
GH67	1(1)	1(1)	1(1)	1(1)	1(1)	1(1)	0	0	0
GH7	3(3)	3(3)	3(3)	2(2)	2(2)	2(2)	0	1(1)	0
GH7, CBM1	3(3)	3(3)	3(3)	3(3)	3(3)	3(3)	0	0	0
GH71, CBM24	2(1)	2(1)	2(1)	2(1)	2(1)	2(1)	0	0	0
GH72	3(1)	3(1)	3(1)	3(1)	3(1)	3(1)	0	0	0
GH72, CBM43	1(0)	1(0)	1(0)	1(0)	1(0)	1(0)	0	0	0
GH74	2(1)	1(1)	3(2)	2(1)	1(1)	3(2)	0	0	0
GH74, CBM1	1(1)	1(1)	0	1(1)	1(1)	0	0	0	0
GH74, GH33	1(1)	1(0)	1(0)	1(1)	1(0)	1(0)	0	0	0
GH75	1(1)	1(1)	1(0)	1(1)	1(1)	1(0)	0	0	0
GH76	8(2)	8(2)	8(2)	8(2)	8(2)	8(2)	0	0	0
GH78	3(3)	5(4)	4(3)	3(3)	4(3)	4(3)	0	1(1)	0
GH79	2(1)	2(1)	2(1)	2(1)	2(1)	2(1)	0	0	0
GH81	2(1)	2(1)	2(1)	1(1)	1(1)	1(1)	0	0	1(0)
GH88	4(0)	3(0)	4(0)	3(0)	3(0)	3(0)	1(0)	0	0

GH92	3(1)	3(0)	3(0)	3(1)	3(0)	3(0)	0	0	0
GH93	1(1)	1(1)	1(1)	1(1)	1(1)	1(1)	0	0	0
GH95	1(0)	2(1)	2(1)	1(0)	1(0)	1(0)	0	0	0
GT1	9(0)	10(0)	10(0)	9(0)	9(0)	9(0)	0	1(0)	1(0)
GT15	5(1)	5(1)	5(1)	4(1)	4(1)	4(1)	0	0	1(0)
GT2	17(0)	17(0)	16(0)	15(0)	14(0)	13(0)	0	1(0)	0
GT20	3(0)	2(0)	2(0)	3(0)	2(0)	2(0)	0	0	0
GT21	1(0)	1(0)	1(0)	1(0)	1(0)	1(0)	0	0	0
GT22	4(0)	4(0)	4(0)	4(0)	4(0)	4(0)	0	0	0
GT24	1(0)	1(0)	1(0)	1(0)	1(0)	1(0)	0	0	0
GT28	1(0)	1(0)	1(0)	1(0)	1(0)	1(0)	0	0	0
GT3	1(0)	1(0)	1(0)	1(0)	1(0)	1(0)	0	0	0
GT31	7(0)	6(0)	6(0)	6(0)	6(0)	5(0)	0	0	0
GT32	4(0)	5(0)	4(0)	4(0)	4(0)	3(0)	0	0	0
GT33	1(0)	1(0)	1(0)	1(0)	1(0)	1(0)	0	0	0
GT34	6(0)	6(0)	6(0)	6(0)	6(0)	6(0)	0	0	0
GT35	1(0)	1(0)	1(0)	1(0)	1(0)	1(0)	0	0	0
GT39	3(0)	3(0)	3(0)	3(0)	3(0)	3(0)	0	0	0
GT4	6(0)	6(0)	6(0)	5(0)	5(0)	5(0)	0	0	0
GT41	2(0)	2(0)	2(0)	2(0)	2(0)	2(0)	0	0	0
GT48	1(0)	1(0)	1(0)	0	0	0	0	0	0
GT50	1(0)	1(0)	1(0)	1(0)	1(0)	1(0)	0	0	0
GT57	2(0)	2(0)	2(0)	2(0)	2(0)	2(0)	0	0	0
GT58	1(0)	1(0)	1(0)	1(0)	1(0)	1(0)	0	0	0
GT59	1(0)	1(0)	1(0)	1(0)	1(0)	1(0)	0	0	0

GT62	3(0)	3(0)	3(0)	3(0)	3(0)	3(0)	0	0	0
GT64	2(0)	2(0)	2(0)	2(0)	2(0)	2(0)	0	0	0
GT66	1(0)	1(0)	1(0)	1(0)	1(0)	1(0)	0	0	0
GT69	2(0)	2(0)	2(0)	2(0)	2(0)	2(0)	0	0	0
GT76	1(0)	1(0)	1(0)	1(0)	1(0)	1(0)	0	0	0
GT8	4(0)	4(0)	4(0)	3(0)	3(0)	3(0)	0	0	1(0)
GT90	5(0)	6(0)	5(0)	4(0)	5(0)	5(0)	0	0	0
PL1	15(14)	15(14)	14(13)	14(14)	14(13)	14(13)	0	1(1)	0
PL1, CBM1	1(1)	0	1(1)	0	0	0	0	0	0
PL11	0	0	1(0)	0	0	0	0	0	0
PL3	11(9)	10(8)	10(8)	9(8)	8(7)	8(7)	2(1)	0	0
PL4	4(4)	4(3)	5(3)	3(3)	3(2)	3(2)	0	0	2(1)
PL9	4(4)	2(2)	2(2)	2(2)	2(2)	2(2)	2(2)	0	0

Note: Annotation using HMM-based routine of the Carbohydrate-Active-EnZymes database (http://www.cazy. org/, Cantarel BL, Coutinho PM, Rancurel C, Bernard T, Lombard V, Henrissat B. 2009. The Carbohydrate-Active enZymes database (CAZy): an expert resource for glycogenomics. Nucleic Acids Res 37: D233-D238) and BLASTP (Altschul SF, Madden TL, Schaffer AA, Zhang J, Zhang Z, Miller W, Lipman DJ. 1997. Gapped BLAST and PSI-BLAST: a new generation of protein database search programs. Nucleic Acids Research 25:3389–3402); Number in the bracket are secreted protein.

Table S14. Protein kinases annotation among the three genomes of *V. dahliae* isolates Vd991, JR2 and VdLs.17.

	Total genes			Common genes			Specific genes		
	Vd991	JR2	VdLs.17	Vd991	JR2	VdLs.17	Vd991	JR2	VdLs.17
Protein Kinase[#]	133	131	145	107	106	107	19	8	18
AGC	18	23	24	18	19	18	0	2	3
CMGC	30	33	34	21	23	22	6	2	5
CAMK	38	36	41	31	29	31	6	2	4
CK1	6	6	7	4	4	4	2	1	2
STE	17	14	16	13	13	13	4	0	3
TK	4	1	3	1	0	0	0	1	1
T-KL	8	6	8	7	6	7	1	0	0
PDHK	3	3	3	3	3	3	0	0	0
PIKK	7	7	7	7	7	7	0	0	0
RIO	2	2	2	2	2	2	0	0	0

[#]Annotation with Kinomer (Version 1.0, Martin DM, Miranda-Saavedra D, Barton GJ. Kinomer v. 1.0: a database of systematically classified eukaryotic protein kinases. Nucleic Acids Res. 2009 Jan;37(Database issue):D244-250);

AGC: cyclic-nucleotide and calcium-phospholipid-dependent kinases, ribosomal S6-phosphorylating kinases, G protein-coupled kinases, and all close relatives of these groups;

CAMK: calmodulin-regulated kinases;

CK1: casein kinase 1;

CMGC: cyclin-dependent kinases, mitogen-activated protein kinases, CDK-like kinases and glycogen synthase kinase;

PDHK: pyruvate dehydrogenase kinases; PIKK: phosphatidyl inositol 30 kinase-related kinases;

RIO: "right open reading frame" as it was one of two adjacent genes that were found to be transcribed divergently from the same intergenic region 7;

STE: including many kinases functioning in MAP kinase cascades;

TK: tyrosine kinases;

TKL: tyrosine kinase-like kinases

Table S15. Annotations of transcription factors among the three genomes of *V. dahliae* isolates Vd991, JR2 and VdLs.17.

	Total genes			Common genes			Specific genes		
	Vd991	JR2	VdLs.17	Vd991	JR2	VdLs.17	Vd991	JR2	VdLs.17
Total TFs	452	503	489	373	390	373	32	35	59
APSES	5	5	5	5	5	5	0	0	0
AT-rich interaction region	4	4	4	4	4	4	0	0	0
Bromodomain transcription factor	2	3	2	2	3	2	0	0	0
bZIP	22	22	24	12	12	13	3	0	9
C_2H_2 zinc finger	64	70	67	53	54	51	5	7	10
CCR4-Not complex component, Not1	1	1	1	1	1	1	0	0	0
Centromere protein B, DNA-binding region	2	6	3	2	2	2	0	4	1
DDT	1	1	1	0	0	0	0	0	0
Forkhead	4	4	4	4	4	4	0	0	0
GATA type zinc finger	7	6	6	4	3	3	0	0	0
GCN5-like 1	1	1	1	1	1	1	0	0	0
Grainyhead/CP2	1	1	1	1	1	1	0	0	0
Helix-turn-helix type 3	1	1	1	1	1	1	0	0	0
Heteromeric CCAAT factors	7	7	6	5	4	4	0	0	1
HMG	9	9	10	8	8	7	1	0	1
Homeobox	9	13	14	9	9	9	0	0	1
Homeodomain-like	7	5	5	6	4	4	1	1	0
Lambda repressor-like, DNA-binding	1	1	1	1	1	1	0	0	0
MADS-box	9	4	7	2	2	2	6	2	5
Myb	14	18	20	13	15	17	0	0	1

Negative transcriptional regulator	1	1	2	1	1	1	0	0	1
p53-like transcription factor	3	3	3	3	3	3	0	0	0
RFX DNA-binding domain	1	1	1	1	1	1	0	0	0
SART1	1	1	1	1	1	1	0	0	0
SGT1	1	1	1	1	1	1	0	0	0
ssDNA-binding transcriptional regulator	1	1	1	1	1	1	0	0	0
TEA/ATTS	1	1	1	1	1	1	0	0	0
Transcription factor TFIIS	4	4	2	2	2	2	1	1	0
Winged helix repressor DNA-binding	35	34	35	33	34	34	2	0	1
YL1 nuclear protein	1	1	1	1	1	1	0	0	0
Zinc finger, BED-type predicted	0	1	1	0	0	0	0	0	0
Zinc finger, CCHC-type	12	17	10	10	11	10	2	6	0
Zinc finger, DHHC-type	6	6	6	6	6	6	0	0	0
Zinc finger, GRF-type	2	2	2	2	2	2	0	0	0
Zinc finger, MIZ-type	3	3	2	1	1	1	1	0	0
Zinc finger, NF-X1-type	0	1	1	0	1	1	0	0	0
Zinc finger, PARP-type	1	1	1	0	0	1	0	0	0
Zinc finger, Rad18-type putative	2	3	0	1	1	0	0	0	0
Zn_2Cys_6	206	239	235	174	188	174	10	14	28

Note: the transcription factors were annotated by the domain prediction of InterProScan 5 (Jones P , Binns D, Chang HY, Fraser M, Li W, McAnulla C, McWilliam H, Maslen J, Mitchell A, Nuka G, Pesseat S, Quinn AF, Sangrador-Vegas A, Scheremetjew M, Yong SY, Lopez R, Hunter S. 2014. InterProScan 5: genome-scale protein function classification. Bioinformatics. 30:1236-1240).

Table S16. Percentages of protein-coding genes with functional annotations in the genomes of the three *V. dahliae* isolates Vd991, JR2 and VdLs.17.

	Annotation database		
	fuNOG*	Gene Ontology$	InterPro$
**Vd991**	**7934 (80.81%)**	**5726 (58.32%)**	**7343 (74.79%)**
JR2	8116 (73.88%)	5885 (53.57%)	7522 (68.48%)
VdLs.17	8148 (77.34%)	5932 (56.31%)	7565 (71.81%)
**Vd991**	**6899 (85.71%)**	**5010 (62.24%)**	**6408 (79.61%)**
JR2	6903 (85.76%)	5037 (62.58%)	6407 (79.60%)
VdLs.17	6908 (85.82%)	5034 (62.54%)	6395 (79.45%)
**Vd991**	**448 (46.67%)**	**311 (32.40%)**	**416 (43.33%)**
JR2	338 (20.46%)	252 (15.25%)	320 (19.37%)
VdLs.17	529 (38.06%)	401 (28.85%)	498 (35.83%)

* Annotation with fuNOG v3.0, http://eggnog.embl.de/version_3.0/; $ Annotation with InterProScan V5. The bold text represents the information of Vd991 genome.

Table S17. Pathogenicity-related factors in the lineage specific regions (LSRs) in the genomes of *V. dahliae* isolates Vd991, JR2 and VdLs.17.

Strains	LS regions	Coverage genes	Number of encoding genes	Pathogenicity-related factors	Secreted proteins	LysM	NLP	SCRPs	CAZymes	PHI	PKs	TFs
Vd991	G-LSR1	VEDA_04892 - VEDA_04923	32	3	0	0	0	0	1	0	0	2
Vd991	G-LSR2	VEDA_05181 - VEDA_05203	23	6	3	0	0	0	1	2	1	1
Vd991	G-LSR3	VEDA_06170 - VEDA_06270	101	21	6	0	0	1	0	2	7	7
Vd991	G-LSR4	VEDA_06654 - VEDA_06713	60	8	2	0	0	0	0	0	3	3
JR2	S-LSR1	evm.model.contig44576.973 - 44576.1042	70	10	4	0	0	4	0	1	1	4
JR2	S-LSR2	evm.model.contig1569.316 - 1569.360	46	10	3	0	0	1	0	0	2	5
JR2	S-LSR3	evm.model.contig45503.186 - 45503.242	57	10	2	0	0	0	3	0	0	6
JR2	S-LSR4	evm.model.contig45503.948 - 45503.990	43	9	0	0	0	0	3	1	2	3
VdLs.17	L-LSR1	VDAG_02345 - VDAG_02433	89	18	3	0	0	1	4	4	3	9
VdLs.17	L-LSR2	VDAG_04831 - VDAG_04927	97	14	3	0	1	2	2	3	1	6
VdLs.17	L-LSR3	VDAG_05143 - VDAG_05291	149	24	1	1	0	1	0	2	6	15
VdLs.17	L-LSR4	VDAG_09118 - VDAG_09227	110	10	3	0	0	1	1	0	2	6

Table S19. List of Vd991 genes homologous to genes from other fungi rather than other *Verticillium* spp.

Gene-ID	NR_ID	NR_define	Species	Identity	*E*-value	LSRs
VEDA_00257	gi|342878268|gb|EGU79623.1|	hypothetical protein	*Fusarium oxysporum* Fo5176	73.19	0	
VEDA_01110	gi|302891093|ref|XP_003044429.1|	hypothetical protein	*Nectria haematococca* mpVI 77-13-4	79.76	5E-41	
VEDA_01409	gi|342886136|gb|EGU86064.1|	hypothetical protein	*Fusarium oxysporum* Fo5176	74.27	0	
VEDA_01555	gi|310795553|gb|EFQ31014.1|	LSM domain-containing protein	*Colletotrichum graminicola* M1.001	96.55	1E-54	
VEDA_02172	gi|477530038|gb|ENH81780.1|	hypothetical protein	*Colletotrichum orbiculare* MAFF 240422	76.92	1E-24	
VEDA_02452	gi|530476025|gb|EQB55989.1|	methyltransferase domain-containing protein	*Colletotrichum gloeosporioides* Cg-14	73.46	4E-174	
VEDA_02491	gi|358388261|gb|EHK25855.1|	serine threonine protein kinase, CMGC group	*Trichoderma virens* Gv29-8	74.41	8E-110	
VEDA_02576	gi|751745657|gb|KIM93851.1|	hypothetical protein	*Oidiodendron maius* Zn	88.82	0	
VEDA_02653	gi|530462071|gb|EQB45048.1|	hypothetical protein	*Colletotrichum gloeosporioides* Cg-14	86.95	0	
VEDA_03089	gi|596719724|ref|XP_007285989.1|	GTPase-activator protein for Ras-like GTPase, partial	*Colletotrichum gloeosporioides* Nara gc5	79.04	0	
VEDA_03864	gi|380474097|emb|CCF45959.1|	hypothetical protein	*Colletotrichum higginsianum*	73.91	1E-39	
VEDA_04201	gi|596672602|ref|XP_007277272.1|	hypothetical protein	*Colletotrichum gloeosporioides* Nara gc5	70.41	2E-130	
VEDA_04353	gi|666403572|gb|KEY69233.1|	hypothetical protein	*Stachybotrys chartarum* IBT 7711	76.06	2E-29	
VEDA_04439	gi|477537128|gb|ENH88588.1|	small nuclear ribonucleoprotein lsm2	*Colletotrichum orbiculare* MAFF 240422	94.9	4E-62	
VEDA_04670	gi|310791025|gb|EFQ26558.1|	protein phosphatase 2C, partial	*Colletotrichum graminicola* M1.001	72.06	0	
VEDA_04893	gi|380492722|emb|CCF34392.1|	hypothetical protein	*Colletotrichum higginsianum*	74.56	0	G-LSR1
VEDA_04912	gi|342876367|gb|EGU77990.1|	hypothetical protein	*Fusarium oxysporum* Fo5176	97.22	4E-41	G-LSR1
VEDA_04916	gi|615440362|ref|XP_007591461.1|	DNA repair helicase	*Colletotrichum fioriniae* PJ7	92.41	0	G-LSR1
VEDA_04920	gi|85077297|ref|XP_956003.1|	histone H3	*Neurospora crassa* OR74A	100	1E-93	G-LSR1
VEDA_04921	gi|699035063|emb|CEF85879.1|	unnamed protein product	*Fusarium graminearum*	99.03	2E-65	G-LSR1
VEDA_05182	gi|590051258|gb|EXK78782.1|	hypothetical protein	*Fusarium oxysporum* f. sp. *raphani* NRRL 54005	79.46	0	G-LSR2
VEDA_05187	gi|512187464|gb|EPE03243.1|	serine threonine protein kinase	*Ophiostoma piceae* UAMH 11346	84.83	0	G-LSR2
VEDA_05190	gi|591404648|gb|EXL39785.1|	hypothetical protein	*Fusarium oxysporum* f. sp. *radicis-lycopersici* NRRL 26381	78.24	0	G-LSR2
VEDA_05191	gi|590050117|gb|EXK77641.1|	hypothetical protein	*Fusarium oxysporum* f. sp. *raphani* NRRL 54005	86.53	1E-113	G-LSR2

VEDA_05192	gi|591405307|gb|EXL40444.1|	hypothetical protein	*Fusarium oxysporum* f. sp. *radicis-lycopersici* NRRL 26381	85.44	0	G-LSR2
VEDA_05193	gi|591483560|gb|EXM13674.1|	hypothetical protein	*Fusarium oxysporum* f. sp. *vasinfectum* NRRL 25433	99.73	0	G-LSR2
VEDA_05194	gi|682380930|gb|KFY64054.1|	hypothetical protein	*Pseudogymnoascus pannorum* VKM F-4515 (FW-2607)	81.47	0	G-LSR2
VEDA_05195	gi|591483564|gb|EXM13678.1|	hypothetical protein	*Fusarium oxysporum* f. sp. *vasinfectum* NRRL 25433	88	0	G-LSR2
VEDA_05196	gi|591484976|gb|EXM14904.1|	hypothetical protein	*Fusarium oxysporum* f. sp. *vasinfectum* NRRL 25433	99.22	0	G-LSR2
VEDA_05197	gi|591484975|gb|EXM14903.1|	hypothetical protein	*Fusarium oxysporum* f. sp. *vasinfectum* NRRL 25433	99.13	0	G-LSR2
VEDA_05198	gi|591484974|gb|EXM14902.1|	hypothetical protein	*Fusarium oxysporum* f. sp. *vasinfectum* NRRL 25433	98.8	0	G-LSR2
VEDA_05199	gi|591484973|gb|EXM14901.1|	hypothetical protein	*Fusarium oxysporum* f. sp. *vasinfectum* NRRL 25433	97.47	0	G-LSR2
VEDA_05200	gi|302913666|ref|XP_003050975.1|	hypothetical protein	*Nectria haematococca* mpVI 77-13-4	72.97	2E-112	G-LSR2
VEDA_05202	gi|743656380|gb|KID83784.1|	ATP-dependent DNA helicase PIF1	*Metarhizium guizhouense* ARSEF 977	89.9	0	G-LSR2
VEDA_05203	gi|590055895|gb|EXK83419.1|	hypothetical protein	*Fusarium oxysporum* f. sp. *raphani* NRRL 54005	76.59	0	G-LSR2
VEDA_05891	gi|685858595|ref|XP_009256330.1|	hypothetical protein	*Fusarium pseudograminearum* CS3096	70.77	2E-54	
VEDA_06249	gi|310792557|gb|EFQ28084.1|	protein kinase domain-containing protein	*Colletotrichum graminicola* M1.001	84.51	0	G-LSR3
VEDA_06267	gi|154272351|ref|XP_001537028.1|	predicted protein	*Histoplasma capsulatum* NAm1	72.8	0	G-LSR3
VEDA_06269	gi|477525916|gb|ENH77788.1|	ABC multidrug transporter	*Colletotrichum orbiculare* MAFF 240422	91.61	0	G-LSR3
VEDA_06270	gi|590051258|gb|EXK78782.1|	hypothetical protein	*Fusarium oxysporum* f. sp. *raphani* NRRL 54005	81.86	0	G-LSR3
VEDA_06316	gi|666870284|gb|KEZ46387.1|	putative Bifunctional fatty acid transporter/acyl-CoA synthetase	*Scedosporium apiospermum*	71.76	0	
VEDA_06571	gi|342876367|gb|EGU77990.1|	hypothetical protein	*Fusarium oxysporum* Fo5176	97.22	4E-41	
VEDA_06677	gi|590022251|gb|EXK24109.1|	hypothetical protein	*Fusarium oxysporum* f. sp. *melonis* NRRL 26406	76.67	1E-144	G-LSR4
VEDA_06689	gi|590023180|gb|EXK25038.1|	hypothetical protein	*Fusarium oxysporum* f. sp. *melonis* NRRL 26406	94.59	0	G-LSR4
VEDA_06690	gi|590023181|gb|EXK25039.1|	hypothetical protein	*Fusarium oxysporum* f. sp. *melonis* NRRL 26406	83.57	1E-80	G-LSR4
VEDA_06691	gi|590022313|gb|EXK24171.1|	hypothetical protein	*Fusarium oxysporum* f. sp. *melonis* NRRL 26406	91.76	0	G-LSR4
VEDA_06692	gi|590022312|gb|EXK24170.1|	hypothetical protein	*Fusarium oxysporum* f. sp. *melonis* NRRL 26406	92.97	0	G-LSR4
VEDA_06693	gi|590022311|gb|EXK24169.1|	hypothetical protein	*Fusarium oxysporum* f. sp. *melonis* NRRL 26406	84.95	0	G-LSR4
VEDA_06712	gi|116194860|ref|XP_001223242.1|	predicted protein	*Chaetomium globosum* CBS 148.51	71.84	1E-92	G-LSR4
VEDA_06797	gi|587663149|gb|EWY85490.1|	hypothetical protein	*Fusarium oxysporum* FOSC 3-a	85.38	8E-107	

VEDA_06798	gi|591497510|gb|EXM26980.1|	hypothetical protein	*Fusarium oxysporum* f. sp. *vasinfectum* NRRL 25433	77.51	0
VEDA_07052	gi|310791459|gb|EFQ26986.1|	PX domain-containing protein	*Colletotrichum graminicola* M1.001	74.19	0
VEDA_07753	gi|302902149|ref|XP_003048592.1|	predicted protein	*Nectria haematococca* mpVI 77-13-4	74.44	0
VEDA_07811	gi|596658526|ref|XP_007274630.1|	60s ribosomal protein l13	*Colletotrichum gloeosporioides* Nara gc5	75	5E-24
VEDA_09414	gi|640923562|gb|KDN67714.1|	putative asparaginase	*Colletotrichum sublineola*	70.76	6E-173
VEDA_09629	gi|590051258|gb|EXK78782.1|	hypothetical protein	*Fusarium oxysporum* f. sp. *raphani* NRRL 54005	80	0
VEDA_09633	gi|591484035|gb|EXM14074.1|	hypothetical protein	*Fusarium oxysporum* f. sp. *vasinfectum* NRRL 25433	76.16	3E-175
VEDA_09635	gi|302881203|ref|XP_003039520.1|	predicted protein	*Nectria haematococca* mpVI 77-13-4	94.67	7E-99
VEDA_09636	gi|591404641|gb|EXL39778.1|	hypothetical protein	*Fusarium oxysporum* f. sp. *radicis-lycopersici* NRRL 26381	84.8	0
VEDA_09637	gi|591404648|gb|EXL39785.1|	hypothetical protein	*Fusarium oxysporum* f. sp. *radicis-lycopersici* NRRL 26381	76.28	0
VEDA_09640	gi|591458520|gb|EXL90226.1|	hypothetical protein	*Fusarium oxysporum* f. sp. *cubense* tropical race 4 NRRL 54006	81.82	0
VEDA_09767	gi|629666091|ref|XP_007795292.1|	putative atp synthase delta mitochondrial protein	*Eutypa lata* UCREL1	87.35	2E-97
VEDA_09780	gi|751354823|gb|KIL92545.1|	ras-like protein	*Fusarium avenaceum*	98.01	4E-145
VEDA_09805	gi|667728547|gb|KFA69517.1|	hypothetical protein	*Stachybotrys chlorohalonata* IBT 40285	79.19	0

Table S21. BLAST analysis of seven genes in G-LSR2 by the *nr* database.

Gene-ID	Scaffold:position	GeneBank-ID	Description	Species	Coverage	Identities	E-value
VEDA_05193	Scaffold4:317053-318168	EXM13674.1	hypothetical protein	*Fusarium oxysporum* f. sp. *vasinfectum* NRRL 25433	100	99.73	0
VEDA_05194	Scaffold4:323081-324022	KFY64054.1	hypothetical protein	*Pseudogymnoascus pannorum* VKM F-4515 FW-2607	100	81.47	0
VEDA_05195	Scaffold4:327568-328705	EXM13678.1	hypothetical protein	*Fusarium oxysporum* f. sp. *vasinfectum* NRRL 25433	97	88	0
VEDA_05196	Scaffold4:332126-333935	EXM14904.1	hypothetical protein	*Fusarium oxysporum* f. sp. *vasinfectum* NRRL 25433	100	99.22	0
VEDA_05197	Scaffold4:335027-336067	EXM14903.1	hypothetical protein	*Fusarium oxysporum* f. sp. *vasinfectum* NRRL 25433	100	99.13	0
VEDA_05198	Scaffold4:336752-337753	EXM14902.1	hypothetical protein	*Fusarium oxysporum* f. sp. *vasinfectum* NRRL 25433	100	98.8	0
VEDA_05199	Scaffold4:338456-339726	EXM14901.1	hypothetical protein	*Fusarium oxysporum* f. sp. *vasinfectum* NRRL 25433	100	97.47	0
