# Supplementary material for: Comparative genomics reveals cotton‐specific virulence factors in flexible genomic regions in Verticillium dahliae and evidence of horizontal gene transfer from Fusarium
Source: New Phytol. 2017 Oct 30;217(2):756–70. doi: 10.1111/nph.14861 (PMC5765495; doi:10.1111/nph.14861)
Supplement: Supplementary file 2 [file NPH-217-756-s002.pdf]

Table S9. Gene synteny among the three genomes of *Verticillium dahliae* Vd991, JR2, and VdLs.17

Vd991 vs JR2 and VdLs.17

Scaffold-ID	Star	End	Gene-ID	Type#	Similarity or coverage ratio lower than 50%*	Specific of orthologue clusters*	Locus variation*	JR2	Similarity	Coverage Ratio of JR2	Coverage Ratio of Vd991	VdLs.17	Similarity	Coverage Ratio of VdLs.17	Coverage Ratio of Vd991
[illegible]	[illegible]	[illegible]	[illegible]	[illegible]	[illegible]	[illegible]	[illegible]	[illegible]	[illegible]	[illegible]	[illegible]	[illegible]	[illegible]	[illegible]	[illegible]

JR2 vs Vd991 and VdLs.17

Chr	Star	End	JR2	Type	Similarity or coverage ratio lower than 50%	Specific of orthologue clusters	Locus variation	Vd991	Similarity	Coverage Ratio of Vd991	Coverage Ratio of JR2	VdLs.17	Similarity	Coverage Ratio of VdLs.17	Coverage Ratio of JR2
[illegible]	[illegible]	[illegible]	[illegible]	[illegible]	[illegible]	[illegible]	[illegible]	[illegible]	[illegible]	[illegible]	[illegible]	[illegible]	[illegible]	[illegible]	[illegible]

VdLs.17 vs Vd991 and JR2

Chr	Scaffold	Star	End	Gene-ID	Type	Similarity or coverage ratio lower than 50%	Specific of orthologue clusters	Locus variation	Vd991	Similarity	Coverage Ratio of Vd991	Coverage Ratio of VdLs.17	JR2	Similarity	Coverage Ratio of JR2	Coverage Ratio of VdLs.17
[illegible]	[illegible]	[illegible]	[illegible]	[illegible]	[illegible]	[illegible]	[illegible]	[illegible]	[illegible]	[illegible]	[illegible]	[illegible]	[illegible]	[illegible]	[illegible]	[illegible]

Vd991 vs JR2 and VdLs.17															
Scaffold-ID	Star	End	Gene-ID	Type#	Similarity or coverage ratio lower than 50%*	Specific of orthologue clusters*	Locus variation*	JR2	Similarity	Coverage Ratio of JR2	Coverage Ratio of Vd991	VdLs.17	Similarity	Coverage Ratio of VdLs.17	Coverage Ratio of Vd991
[illegible]	[illegible]	[illegible]	[illegible]	[illegible]	[illegible]	[illegible]	[illegible]	[illegible]	[illegible]	[illegible]	[illegible]	[illegible]	[illegible]	[illegible]	[illegible]

JR2 vs Vd991 and VdLs.17															
Chr	Star	End	JR2	Type	Similarity or coverage ratio lower than 50%	Specific of orthologue clusters	Locus variation	Vd991	Similarity	Coverage Ratio of Vd991	Coverage Ratio of JR2	VdLs.17	Similarity	Coverage Ratio of VdLs.17	Coverage Ratio of JR2
[illegible]	[illegible]	[illegible]	[illegible]	[illegible]	[illegible]	[illegible]	[illegible]	[illegible]	[illegible]	[illegible]	[illegible]	[illegible]	[illegible]	[illegible]	[illegible]

VdLs.17 vs Vd991 and JR2																
Chr	Scaffold	Star	End	Gene-ID	Type	Similarity or coverage ratio lower than 50%	Specific of orthologue clusters	Locus variation	Vd991	Similarity	Coverage Ratio of Vd991	Coverage Ratio of VdLs.17	JR2	Similarity	Coverage Ratio of JR2	Coverage Ratio of VdLs.17
[illegible]	[illegible]	[illegible]	[illegible]	[illegible]	[illegible]	[illegible]	[illegible]	[illegible]	[illegible]	[illegible]	[illegible]	[illegible]	[illegible]	[illegible]	[illegible]	[illegible]

								Vd991 vs JR2 and VdLs.17							
Scaffold-ID	Star	End	Gene-ID	Type[a]	Similarity or coverage ratio lower than 50%[b]	Specific of orthologue clusters[c]	Locus variation[d]	JR2	Similarity	Coverage Ratio of JR2	Coverage Ratio of Vd991	VdLs.17	Similarity	Coverage Ratio of VdLs.17	Coverage Ratio of Vd991
[illegible]	[illegible]	[illegible]	[illegible]	[illegible]	[illegible]	[illegible]	[illegible]	[illegible]	[illegible]	[illegible]	[illegible]	[illegible]	[illegible]	[illegible]	[illegible]

								JR2 vs Vd991 and VdLs.17							
Chr	Star	End	JR2	Type	Similarity or coverage ratio lower than 50%	Specific of orthologue clusters	Locus variation	Vd991	Similarity	Coverage Ratio of Vd991	Coverage Ratio of JR2	VdLs.17	Similarity	Coverage Ratio of VdLs.17	Coverage Ratio of JR2
[illegible]	[illegible]	[illegible]	[illegible]	[illegible]	[illegible]	[illegible]	[illegible]	[illegible]	[illegible]	[illegible]	[illegible]	[illegible]	[illegible]	[illegible]	[illegible]

									VdLs.17 vs Vd991 and JR2							
Chr	Scaffold	Star	End	Gene-ID	Type	Similarity or coverage ratio lower than 50%	Specific of orthologue clusters	Locus variation	Vd991	Similarity	Coverage Ratio of Vd991	Coverage Ratio of VdLs.17	JR2	Similarity	Coverage Ratio of JR2	Coverage Ratio of VdLs.17
[illegible]	[illegible]	[illegible]	[illegible]	[illegible]	[illegible]	[illegible]	[illegible]	[illegible]	[illegible]	[illegible]	[illegible]	[illegible]	[illegible]	[illegible]	[illegible]	[illegible]

Vd991 vs JR2 and VdLs.17																	
Scaffold-ID	Star	End	Gene-ID	Type[a]	Similarity or coverage ratio lower than 50%[*]	Specific of orthologue clusters[*]	Locus variation[*]	JR2	Similarity	Coverage Ratio of JR2	Coverage Ratio of Vd991	VdLs.17	Similarity	Coverage Ratio of VdLs.17	Coverage Ratio of Vd991		
[illegible]	[illegible]	[illegible]	[illegible]	[illegible]	[illegible]	[illegible]	[illegible]	[illegible]	[illegible]	[illegible]	[illegible]	[illegible]	[illegible]	[illegible]	[illegible]		

JR2 vs Vd991 and VdLs.17																	
Chr	Star	End	JR2	Type	Similarity or coverage ratio lower than 50%	Specific of orthologue clusters	Locus variation	Vd991	Similarity	Coverage Ratio of Vd991	Coverage Ratio of JR2	VdLs.17	Similarity	Coverage Ratio of VdLs.17	Coverage Ratio of JR2		
[illegible]	[illegible]	[illegible]	[illegible]	[illegible]	[illegible]	[illegible]	[illegible]	[illegible]	[illegible]	[illegible]	[illegible]	[illegible]	[illegible]	[illegible]	[illegible]		

VdLs.17 vs Vd991 and JR2																	
Chr	Scaffold	Star	End	Gene-ID	Type	Similarity or coverage ratio lower than 50%	Specific of orthologue clusters	Locus variation	Vd991	Similarity	Coverage Ratio of Vd991	Coverage Ratio of VdLs.17	JR2	Similarity	Coverage Ratio of JR2	Coverage Ratio of VdLs.17	
[illegible]	[illegible]	[illegible]	[illegible]	[illegible]	[illegible]	[illegible]	[illegible]	[illegible]	[illegible]	[illegible]	[illegible]	[illegible]	[illegible]	[illegible]	[illegible]	[illegible]	

Vd991 vs JR2 and VdLs.17															
Scaffold-ID	Star	End	Gene-ID	Type#	Similarity or coverage ratio lower then 50%*	Specific of orthologue clusters*	Locus variation*	JR2	Similarity	Coverage Ratio of JR2	Coverage Ratio of Vd991	VdLs.17	Similarity	Coverage Ratio of VdLs.17	Coverage Ratio of Vd991
[illegible]	[illegible]	[illegible]	[illegible]	[illegible]	[illegible]	[illegible]	[illegible]	[illegible]	[illegible]	[illegible]	[illegible]	[illegible]	[illegible]	[illegible]	[illegible]

JR2 vs Vd991 and VdLs.17															
Chr	Star	End	JR2	Type	Similarity or coverage ratio lower than 50%	Specific of orthologue clusters	Locus variation	Vd991	Similarity	Coverage Ratio of Vd991	Coverage Ratio of JR2	VdLs.17	Similarity	Coverage Ratio of VdLs.17	Coverage Ratio of JR2
[illegible]	[illegible]	[illegible]	[illegible]	[illegible]	[illegible]	[illegible]	[illegible]	[illegible]	[illegible]	[illegible]	[illegible]	[illegible]	[illegible]	[illegible]	[illegible]

VdLs.17 vs Vd991 and JR2																
Chr	Scaffold	Star	End	Gene-ID	Type	Similarity or coverage ratio lower than 50%	Specific of orthologue clusters	Locus variation	Vd991	Similarity	Coverage Ratio of Vd991	Coverage Ratio of VdLs.17	JR2	Similarity	Coverage Ratio of JR2	Coverage Ratio of VdLs.17
[illegible]	[illegible]	[illegible]	[illegible]	[illegible]	[illegible]	[illegible]	[illegible]	[illegible]	[illegible]	[illegible]	[illegible]	[illegible]	[illegible]	[illegible]	[illegible]	[illegible]

Vd991 vs JR2 and VdLs.17

Scaffold-ID	Star	End	Gene-ID	Type[#]	Similarity or coverage ratio lower then 50%[*]	Specific of orthologue clusters[*]	Locus variation[*]	JR2	Similarity	Coverage Ratio of JR2	Coverage Ratio of Vd991	VdLs.17	Similarity	Coverage Ratio of VdLs.17	Coverage Ratio of Vd991
[illegible]	[illegible]	[illegible]	[illegible]	[illegible]	[illegible]	[illegible]	[illegible]	[illegible]	[illegible]	[illegible]	[illegible]	[illegible]	[illegible]	[illegible]	[illegible]

JR2 vs Vd991 and VdLs.17

Chr	Star	End	JR2	Type	Similarity or coverage ratio lower than 50%	Specific of orthologue clusters	Locus variation	Vd991	Similarity	Coverage Ratio of Vd991	Coverage Ratio of JR2	VdLs.17	Similarity	Coverage Ratio of VdLs.17	Coverage Ratio of JR2
[illegible]	[illegible]	[illegible]	[illegible]	[illegible]	[illegible]	[illegible]	[illegible]	[illegible]	[illegible]	[illegible]	[illegible]	[illegible]	[illegible]	[illegible]	[illegible]

VdLs.17 vs Vd991 and JR2

Chr	Scaffold	Star	End	Gene-ID	Type	Similarity or coverage ratio lower than 50%	Specific of orthologue clusters	Locus variation	Vd991	Similarity	Coverage Ratio of Vd991	Coverage Ratio of VdLs.17	JR2	Similarity	Coverage Ratio of JR2	Coverage Ratio of VdLs.17
[illegible]	[illegible]	[illegible]	[illegible]	[illegible]	[illegible]	[illegible]	[illegible]	[illegible]	[illegible]	[illegible]	[illegible]	[illegible]	[illegible]	[illegible]	[illegible]	[illegible]

Vd991 vs JR2 and VdLs.17															
Scaffold-ID	Star	End	Gene-ID	Type	Similarity or coverage ratio lower than 50%*	Specific of orthologue clusters*	Locus variation*	JR2	Similarity	Coverage Ratio of JR2	Coverage Ratio of Vd991	VdLs.17	Similarity	Coverage Ratio of VdLs.17	Coverage Ratio of Vd991
[illegible]	[illegible]	[illegible]	[illegible]	[illegible]	[illegible]	[illegible]	[illegible]	[illegible]	[illegible]	[illegible]	[illegible]	[illegible]	[illegible]	[illegible]	[illegible]

JR2 vs Vd991 and VdLs.17															
Chr	Star	End	JR2	Type	Similarity or coverage ratio lower than 50%	Specific of orthologue clusters	Locus variation	Vd991	Similarity	Coverage Ratio of Vd991	Coverage Ratio of JR2	VdLs.17	Similarity	Coverage Ratio of VdLs.17	Coverage Ratio of JR2
[illegible]	[illegible]	[illegible]	[illegible]	[illegible]	[illegible]	[illegible]	[illegible]	[illegible]	[illegible]	[illegible]	[illegible]	[illegible]	[illegible]	[illegible]	[illegible]

VdLs.17 vs Vd991 and JR2																
Chr	Scaffold	Star	End	Gene-ID	Type	Similarity or coverage ratio lower than 50%	Specific of orthologue clusters	Locus variation	Vd991	Similarity	Coverage Ratio of Vd991	Coverage Ratio of VdLs.17	JR2	Similarity	Coverage Ratio of JR2	Coverage Ratio of VdLs.17
[illegible]	[illegible]	[illegible]	[illegible]	[illegible]	[illegible]	[illegible]	[illegible]	[illegible]	[illegible]	[illegible]	[illegible]	[illegible]	[illegible]	[illegible]	[illegible]	[illegible]

Vd991 vs JR2 and VdLs.17															
Scaffold-ID	Star	End	Gene-ID	Type#	Similarity or coverage ratio lower than 50%*	Specific of orthologue clusters*	Locus variation*	JR2	Similarity	Coverage Ratio of JR2	Coverage Ratio of Vd991	VdLs.17	Similarity	Coverage Ratio of VdLs.17	Coverage Ratio of Vd991
[illegible]	[illegible]	[illegible]	[illegible]	[illegible]	[illegible]	[illegible]	[illegible]	[illegible]	[illegible]	[illegible]	[illegible]	[illegible]	[illegible]	[illegible]	[illegible]

JR2 vs Vd991 and VdLs.17															
Chr	Star	End	JR2	Type	Similarity or coverage ratio lower than 50%	Specific of orthologue clusters	Locus variation	Vd991	Similarity	Coverage Ratio of Vd991	Coverage Ratio of JR2	VdLs.17	Similarity	Coverage Ratio of VdLs.17	Coverage Ratio of JR2
[illegible]	[illegible]	[illegible]	[illegible]	[illegible]	[illegible]	[illegible]	[illegible]	[illegible]	[illegible]	[illegible]	[illegible]	[illegible]	[illegible]	[illegible]	[illegible]

VdLs.17 vs Vd991 and JR2																
Chr	Scaffold	Star	End	Gene-ID	Type	Similarity or coverage ratio lower than 50%	Specific of orthologue clusters	Locus variation	Vd991	Similarity	Coverage Ratio of Vd991	Coverage Ratio of VdLs.17	JR2	Similarity	Coverage Ratio of JR2	Coverage Ratio of VdLs.17
[illegible]	[illegible]	[illegible]	[illegible]	[illegible]	[illegible]	[illegible]	[illegible]	[illegible]	[illegible]	[illegible]	[illegible]	[illegible]	[illegible]	[illegible]	[illegible]	[illegible]

Vd991 vs JR2 and VdLs.17															
Scaffold-ID	Star	End	Gene-ID	Type#	Similarity or coverage ratio lower than 50%*	Specific of orthologue clusters*	Locus variation*	JR2	Similarity	Coverage Ratio of JR2	Coverage Ratio of Vd991	VdLs.17	Similarity	Coverage Ratio of VdLs.17	Coverage Ratio of Vd991
[illegible]	[illegible]	[illegible]	[illegible]	[illegible]	[illegible]	[illegible]	[illegible]	[illegible]	[illegible]	[illegible]	[illegible]	[illegible]	[illegible]	[illegible]	[illegible]

JR2 vs Vd991 and VdLs.17															
Chr	Star	End	JR2	Type	Similarity or coverage ratio lower than 50%	Specific of orthologue clusters	Locus variation	Vd991	Similarity	Coverage Ratio of Vd991	Coverage Ratio of JR2	VdLs.17	Similarity	Coverage Ratio of VdLs.17	Coverage Ratio of JR2
[illegible]	[illegible]	[illegible]	[illegible]	[illegible]	[illegible]	[illegible]	[illegible]	[illegible]	[illegible]	[illegible]	[illegible]	[illegible]	[illegible]	[illegible]	[illegible]

VdLs.17 vs Vd991 and JR2																
Chr	Scaffold	Star	End	Gene-ID	Type	Similarity or coverage ratio lower than 50%	Specific of orthologue clusters	Locus variation	Vd991	Similarity	Coverage Ratio of Vd991	Coverage Ratio of VdLs.17	JR2	Similarity	Coverage Ratio of JR2	Coverage Ratio of VdLs.17
[illegible]	[illegible]	[illegible]	[illegible]	[illegible]	[illegible]	[illegible]	[illegible]	[illegible]	[illegible]	[illegible]	[illegible]	[illegible]	[illegible]	[illegible]	[illegible]	[illegible]

Vd991 vs JR2 and VdLs.17

Scaffold-ID	Star	End	Gene-ID	Type#	Similarity or coverage ratio lower than 50%*	Specific of orthologue clusters*	Locus variation*	JR2	Similarity	Coverage Ratio of JR2	Coverage Ratio of Vd991	VdLs.17	Similarity	Coverage Ratio of VdLs.17	Coverage Ratio of Vd991
[illegible]	[illegible]	[illegible]	[illegible]	[illegible]	[illegible]	[illegible]	[illegible]	[illegible]	[illegible]	[illegible]	[illegible]	[illegible]	[illegible]	[illegible]	[illegible]

JR2 vs Vd991 and VdLs.17

Chr	Star	End	JR2	Type	Similarity or coverage ratio lower than 50%	Specific of orthologue clusters	Locus variation	Vd991	Similarity	Coverage Ratio of Vd991	Coverage Ratio of JR2	VdLs.17	Similarity	Coverage Ratio of VdLs.17	Coverage Ratio of JR2
[illegible]	[illegible]	[illegible]	[illegible]	[illegible]	[illegible]	[illegible]	[illegible]	[illegible]	[illegible]	[illegible]	[illegible]	[illegible]	[illegible]	[illegible]	[illegible]

VdLs.17 vs Vd991 and JR2

Chr	Scaffold	Star	End	Gene-ID	Type	Similarity or coverage ratio lower than 50%	Specific of orthologue clusters	Locus variation	Vd991	Similarity	Coverage Ratio of Vd991	Coverage Ratio of VdLs.17	JR2	Similarity	Coverage Ratio of JR2	Coverage Ratio of VdLs.17
[illegible]	[illegible]	[illegible]	[illegible]	[illegible]	[illegible]	[illegible]	[illegible]	[illegible]	[illegible]	[illegible]	[illegible]	[illegible]	[illegible]	[illegible]	[illegible]	[illegible]

Vd991 vs JR2 and VdLs.17																	
Scaffold-ID	Star	End	Gene-ID	Type[#]	Similarity or coverage ratio lower than 50%[*]	Specific of orthologue clusters[*]	Locus variation[*]	JR2	Similarity	Coverage Ratio of JR2	Coverage Ratio of Vd991	VdLs.17	Similarity	Coverage Ratio of VdLs.17	Coverage Ratio of Vd991		
[illegible]																	

JR2 vs Vd991 and VdLs.17																	
Chr	Star	End	JR2	Type	Similarity or coverage ratio lower than 50%	Specific of orthologue clusters	Locus variation	Vd991	Similarity	Coverage Ratio of Vd991	Coverage Ratio of JR2	VdLs.17	Similarity	Coverage Ratio of VdLs.17	Coverage Ratio of JR2		
[illegible]																	

VdLs.17 vs Vd991 and JR2																	
Chr	Scaffold	Star	End	Gene-ID	Type	Similarity or coverage ratio lower than 50%	Specific of orthologue clusters	Locus variation	Vd991	Similarity	Coverage Ratio of Vd991	Coverage Ratio of VdLs.17	JR2	Similarity	Coverage Ratio of JR2	Coverage Ratio of VdLs.17	
[illegible]																	

Vd991 vs JR2 and VdLs.17

Scaffold-ID	Star	End	Gene-ID	Type[a]	Similarity or coverage ratio lower than 50%[*]	Specific of orthologue clusters[*]	Locus variation[*]	JR2	Similarity	Coverage Ratio of JR2	Coverage Ratio of Vd991	VdLs.17	Similarity	Coverage Ratio of VdLs.17	Coverage Ratio of Vd991
[illegible]	[illegible]	[illegible]	[illegible]	[illegible]	[illegible]	[illegible]	[illegible]	[illegible]	[illegible]	[illegible]	[illegible]	[illegible]	[illegible]	[illegible]	[illegible]

JR2 vs Vd991 and VdLs.17

Chr	Star	End	JR2	Type	Similarity or coverage ratio lower than 50%	Specific of orthologue clusters	Locus variation	Vd991	Similarity	Coverage Ratio of Vd991	Coverage Ratio of JR2	VdLs.17	Similarity	Coverage Ratio of VdLs.17	Coverage Ratio of JR2
[illegible]	[illegible]	[illegible]	[illegible]	[illegible]	[illegible]	[illegible]	[illegible]	[illegible]	[illegible]	[illegible]	[illegible]	[illegible]	[illegible]	[illegible]	[illegible]

VdLs.17 vs Vd991 and JR2

Chr	Scaffold	Star	End	Gene-ID	Type	Similarity or coverage ratio lower than 50%	Specific of orthologue clusters	Locus variation	Vd991	Similarity	Coverage Ratio of Vd991	Coverage Ratio of VdLs.17	JR2	Similarity	Coverage Ratio of JR2	Coverage Ratio of VdLs.17
[illegible]	[illegible]	[illegible]	[illegible]	[illegible]	[illegible]	[illegible]	[illegible]	[illegible]	[illegible]	[illegible]	[illegible]	[illegible]	[illegible]	[illegible]	[illegible]	[illegible]

Scaffold-ID	Star	End	Gene-ID	Type[a]	Similarity or coverage ratio lower than 50%[b]	Specific of orthologue clusters[c]	Locus variation[d]	Vd991 vs JR2 and VdLs.17: JR2	Similarity	Coverage Ratio of JR2	Coverage Ratio of Vd991	VdLs.17	Similarity	Coverage Ratio of VdLs.17	Coverage Ratio of Vd991
[illegible]	[illegible]	[illegible]	[illegible]	[illegible]	[illegible]	[illegible]	[illegible]	[illegible]	[illegible]	[illegible]	[illegible]	[illegible]	[illegible]	[illegible]	[illegible]

Chr	Star	End	JR2	Type	Similarity or coverage ratio lower than 50%	Specific of orthologue clusters	Locus variation	JR2 vs Vd991 and VdLs.17: Vd991	Similarity	Coverage Ratio of Vd991	Coverage Ratio of JR2	VdLs.17	Similarity	Coverage Ratio of VdLs.17	Coverage Ratio of JR2
[illegible]	[illegible]	[illegible]	[illegible]	[illegible]	[illegible]	[illegible]	[illegible]	[illegible]	[illegible]	[illegible]	[illegible]	[illegible]	[illegible]	[illegible]	[illegible]

Chr	Scaffold	Star	End	Gene-ID	Type	Similarity or coverage ratio lower than 50%	Specific of orthologue clusters	Locus variation	VdLs.17 vs Vd991 and JR2: Vd991	Similarity	Coverage Ratio of Vd991	Coverage Ratio of VdLs.17	JR2	Similarity	Coverage Ratio of JR2	Coverage Ratio of VdLs.17
[illegible]	[illegible]	[illegible]	[illegible]	[illegible]	[illegible]	[illegible]	[illegible]	[illegible]	[illegible]	[illegible]	[illegible]	[illegible]	[illegible]	[illegible]	[illegible]	[illegible

Vd991 vs JR2 and VdLs.17																
Scaffold-ID	Star	End	Gene-ID	Type[a]	Similarity or coverage ratio lower then 50%[b]	Specific of orthologue clusters[c]	Locus variation[d]	JR2	Similarity	Coverage Ratio of JR2	Coverage Ratio of Vd991	VdLs.17	Similarity	Coverage Ratio of VdLs.17	Coverage Ratio of Vd991	
[illegible]																

JR2 vs Vd991 and VdLs.17																	
Chr	Star	End	JR2	Type	Similarity or coverage ratio lower than 50%	Specific of orthologue clusters	Locus variation	Vd991	Similarity	Coverage Ratio of Vd991	Coverage Ratio of JR2	VdLs.17	Similarity	Coverage Ratio of VdLs.17	Coverage Ratio of JR2		
[illegible]																	

VdLs.17 vs Vd991 and JR2																	
Chr	Scaffold	Star	End	Gene-ID	Type	Similarity or coverage ratio lower than 50%	Specific of orthologue clusters	Locus variation	Vd991	Similarity	Coverage Ratio of Vd991	Coverage Ratio of VdLs.17	JR2	Similarity	Coverage Ratio of JR2	Coverage Ratio of VdLs.17	
[illegible]																	

Vd991 vs JR2 and VdLs.17															
Scaffold-ID	Star	End	Gene-ID	Type*	Similarity or coverage ratio lower than 50%*	Specific of orthologue clusters*	Locus variation*	JR2	Similarity	Coverage Ratio of JR2	Coverage Ratio of Vd991	VdLs.17	Similarity	Coverage Ratio of VdLs.17	Coverage Ratio of Vd991
[illegible]	[illegible]	[illegible]	[illegible]	[illegible]	[illegible]	[illegible]	[illegible]	[illegible]	[illegible]	[illegible]	[illegible]	[illegible]	[illegible]	[illegible]	[illegible]

JR2 vs Vd991 and VdLs.17															
Chr	Star	End	JR2	Type	Similarity or coverage ratio lower than 50%	Specific of orthologue clusters	Locus variation	Vd991	Similarity	Coverage Ratio of Vd991	Coverage Ratio of JR2	VdLs.17	Similarity	Coverage Ratio of VdLs.17	Coverage Ratio of JR2
[illegible]	[illegible]	[illegible]	[illegible]	[illegible]	[illegible]	[illegible]	[illegible]	[illegible]	[illegible]	[illegible]	[illegible]	[illegible]	[illegible]	[illegible]	[illegible]

VdLs.17 vs Vd991 and JR2																
Chr	Scaffold	Star	End	Gene-ID	Type	Similarity or coverage ratio lower than 50%	Specific of orthologue clusters	Locus variation	Vd991	Similarity	Coverage Ratio of Vd991	Coverage Ratio of VdLs.17	JR2	Similarity	Coverage Ratio of JR2	Coverage Ratio of VdLs.17
[illegible]	[illegible]	[illegible]	[illegible]	[illegible]	[illegible]	[illegible]	[illegible]	[illegible]	[illegible]	[illegible]	[illegible]	[illegible]	[illegible]	[illegible]	[illegible]	[illegible]

Vd991 vs JR2 and VdLs.17															
Scaffold-ID	Star	End	Gene-ID	Type[a]	Similarity or coverage ratio lower than 50%[b]	Specific of orthologue clusters[c]	Locus variation[d]	JR2	Similarity	Coverage Ratio of JR2	Coverage Ratio of Vd991	VdLs.17	Similarity	Coverage Ratio of VdLs.17	Coverage Ratio of Vd991
[illegible]	[illegible]	[illegible]	[illegible]	[illegible]	[illegible]	[illegible]	[illegible]	[illegible]	[illegible]	[illegible]	[illegible]	[illegible]	[illegible]	[illegible]	[illegible]

JR2 vs Vd991 and VdLs.17																
Chr	Star	End	JR2	Type	Similarity or coverage ratio lower than 50%	Specific of orthologue clusters	Locus variation	Vd991	Similarity	Coverage Ratio of Vd991	Coverage Ratio of JR2	VdLs.17	Similarity	Coverage Ratio of VdLs.17	Coverage Ratio of JR2
[illegible]	[illegible]	[illegible]	[illegible]	[illegible]	[illegible]	[illegible]	[illegible]	[illegible]	[illegible]	[illegible]	[illegible]	[illegible]	[illegible]	[illegible]	[illegible]

VdLs.17 vs Vd991 and JR2																	
Chr	Scaffold	Star	End	Gene-ID	Type	Similarity or coverage ratio lower than 50%	Specific of orthologue clusters	Locus variation	Vd991	Similarity	Coverage Ratio of Vd991	Coverage Ratio of VdLs.17	JR2	Similarity	Coverage Ratio of JR2	Coverage Ratio of VdLs.17
[illegible]	[illegible]	[illegible]	[illegible]	[illegible]	[illegible]	[illegible]	[illegible]	[illegible]	[illegible]	[illegible]	[illegible]	[illegible]	[illegible]	[illegible]	[illegible]	[illegible]

Vd991 vs JR2 and VdLs.17																
Scaffold-ID	Star	End	Gene-ID	Type[#]	Similarity or coverage ratio lower than 50%[*]	Specific of orthologue clusters[*]	Locus variation[*]	JR2	Similarity	Coverage Ratio of JR2	Coverage Ratio of Vd991	VdLs.17	Similarity	Coverage Ratio of VdLs.17	Coverage Ratio of Vd991	
[illegible]	[illegible]	[illegible]	[illegible]	[illegible]	[illegible]	[illegible]	[illegible]	[illegible]	[illegible]	[illegible]	[illegible]	[illegible]	[illegible]	[illegible]	[illegible]	

JR2 vs Vd991 and VdLs.17																
Chr	Star	End	JR2	Type	Similarity or coverage ratio lower than 50%	Specific of orthologue clusters	Locus variation	Vd991	Similarity	Coverage Ratio of Vd991	Coverage Ratio of JR2	VdLs.17	Similarity	Coverage Ratio of VdLs.17	Coverage Ratio of JR2	
[illegible]	[illegible]	[illegible]	[illegible]	[illegible]	[illegible]	[illegible]	[illegible]	[illegible]	[illegible]	[illegible]	[illegible]	[illegible]	[illegible]	[illegible]	[illegible]	

VdLs.17 vs Vd991 and JR2																	
Chr	Scaffold	Star	End	Gene-ID	Type	Similarity or coverage ratio lower than 50%	Specific of orthologue clusters	Locus variation	Vd991	Similarity	Coverage Ratio of Vd991	Coverage Ratio of VdLs.17	JR2	Similarity	Coverage Ratio of JR2	Coverage Ratio of VdLs.17	
[illegible]	[illegible]	[illegible]	[illegible]	[illegible]	[illegible]	[illegible]	[illegible]	[illegible]	[illegible]	[illegible]	[illegible]	[illegible]	[illegible]	[illegible]	[illegible]	[illegible]	

Vd991 vs JR2 and VdLs.17															
Scaffold-ID	Star	End	Gene-ID	Type*	Similarity or coverage ratio lower than 50%*	Specific of orthologue clusters*	Locus variation*	JR2	Similarity	Coverage Ratio of JR2	Coverage Ratio of Vd991	VdLs.17	Similarity	Coverage Ratio of VdLs.17	Coverage Ratio of Vd991
[illegible]	[illegible]	[illegible]	[illegible]	[illegible]	[illegible]	[illegible]	[illegible]	[illegible]	[illegible]	[illegible]	[illegible]	[illegible]	[illegible]	[illegible]	[illegible]

JR2 vs Vd991 and VdLs.17															
Chr	Star	End	JR2	Type	Similarity or coverage ratio lower than 50%	Specific of orthologue clusters	Locus variation	Vd991	Similarity	Coverage Ratio of Vd991	Coverage Ratio of JR2	VdLs.17	Similarity	Coverage Ratio of VdLs.17	Coverage Ratio of JR2
[illegible]	[illegible]	[illegible]	[illegible]	[illegible]	[illegible]	[illegible]	[illegible]	[illegible]	[illegible]	[illegible]	[illegible]	[illegible]	[illegible]	[illegible]	[illegible]

VdLs.17 vs Vd991 and JR2																
Chr	Scaffold	Star	End	Gene-ID	Type	Similarity or coverage ratio lower than 50%	Specific of orthologue clusters	Locus variation	Vd991	Similarity	Coverage Ratio of Vd991	Coverage Ratio of VdLs.17	JR2	Similarity	Coverage Ratio of JR2	Coverage Ratio of VdLs.17
[illegible]	[illegible]	[illegible]	[illegible]	[illegible]	[illegible]	[illegible]	[illegible]	[illegible]	[illegible]	[illegible]	[illegible]	[illegible]	[illegible]	[illegible]	[illegible]	[illegible]

[illegible]																	
Scaffold-ID	Star	End	Gene-ID	Type[a]	Similarity or coverage ratio lower than 50%[*]	Specific of orthologue clusters[*]	Locus variation[*]	JR2	Similarity	Coverage Ratio of JR2	Coverage Ratio of Vd991	VdLs.17	Similarity	Coverage Ratio of VdLs.17	Coverage Ratio of Vd991		
[illegible]																	

[illegible]																
Chr	Star	End	JR2	Type	Similarity or coverage ratio lower than 50%	Specific of orthologue clusters	Locus variation	Vd991	Similarity	Coverage Ratio of Vd991	Coverage Ratio of JR2	VdLs.17	Similarity	Coverage Ratio of VdLs.17	Coverage Ratio of JR2	
[illegible]																

[illegible]																	
Chr	Scaffold	Star	End	Gene-ID	Type	Similarity or coverage ratio lower than 50%	Specific of orthologue clusters	Locus variation	Vd991	Similarity	Coverage Ratio of Vd991	Coverage Ratio of VdLs.17	JR2	Similarity	Coverage Ratio of JR2	Coverage Ratio of VdLs.17	
[illegible]																	

Vd991 vs JR2 and VdLs.17																		
Scaffold-ID	Star	End	Gene-ID	Type#	Similarity or coverage ratio lower than 50%*	Specific of orthologue clusters*	Locus variation*	JR2	Similarity	Coverage Ratio of JR2	Coverage Ratio of Vd991	VdLs.17	Similarity	Coverage Ratio of VdLs.17	Coverage Ratio of Vd991			
[illegible]	[illegible]	[illegible]	[illegible]	[illegible]	[illegible]	[illegible]	[illegible]	[illegible]	[illegible]	[illegible]	[illegible]	[illegible]	[illegible]	[illegible]	[illegible]			

JR2 vs Vd991 and VdLs.17																	
Chr	Star	End	JR2	Type	Similarity or coverage ratio lower than 50%	Specific of orthologue clusters	Locus variation	Vd991	Similarity	Coverage Ratio of Vd991	Coverage Ratio of JR2	VdLs.17	Similarity	Coverage Ratio of VdLs.17	Coverage Ratio of JR2		
[illegible]	[illegible]	[illegible]	[illegible]	[illegible]	[illegible]	[illegible]	[illegible]	[illegible]	[illegible]	[illegible]	[illegible]	[illegible]	[illegible]	[illegible]	[illegible]		

VdLs.17 vs Vd991 and JR2																	
Chr	Scaffold	Star	End	Gene-ID	Type	Similarity or coverage ratio lower than 50%	Specific of orthologue clusters	Locus variation	Vd991	Similarity	Coverage Ratio of Vd991	Coverage Ratio of VdLs.17	JR2	Similarity	Coverage Ratio of JR2	Coverage Ratio of VdLs.17	
[illegible]	[illegible]	[illegible]	[illegible]	[illegible]	[illegible]	[illegible]	[illegible]	[illegible]	[illegible]	[illegible]	[illegible]	[illegible]	[illegible]	[illegible]	[illegible]	[illegible]	

Vd991 vs JR2 and VdLs.17															
Scaffold-ID	Star	End	Gene-ID	Type*	Similarity or coverage ratio lower than 50%*	Specific of orthologue clusters*	Locus variation*	JR2	Similarity	Coverage Ratio of JR2	Coverage Ratio of Vd991	VdLs.17	Similarity	Coverage Ratio of VdLs.17	Coverage Ratio of Vd991
[illegible]	[illegible]	[illegible]	[illegible]	[illegible]	[illegible]	[illegible]	[illegible]	[illegible]	[illegible]	[illegible]	[illegible]	[illegible]	[illegible]	[illegible]	[illegible]

JR2 vs Vd991 and VdLs.17															
Chr	Star	End	JR2	Type	Similarity or coverage ratio lower than 50%	Specific of orthologue clusters	Locus variation	Vd991	Similarity	Coverage Ratio of Vd991	Coverage Ratio of JR2	VdLs.17	Similarity	Coverage Ratio of VdLs.17	Coverage Ratio of JR2
[illegible]	[illegible]	[illegible]	[illegible]	[illegible]	[illegible]	[illegible]	[illegible]	[illegible]	[illegible]	[illegible]	[illegible]	[illegible]	[illegible]	[illegible]	[illegible]

VdLs.17 vs Vd991 and JR2																
Chr	Scaffold	Star	End	Gene-ID	Type	Similarity or coverage ratio lower than 50%	Specific of orthologue clusters	Locus variation	Vd991	Similarity	Coverage Ratio of Vd991	Coverage Ratio of VdLs.17	JR2	Similarity	Coverage Ratio of JR2	Coverage Ratio of VdLs.17
[illegible]	[illegible]	[illegible]	[illegible]	[illegible]	[illegible]	[illegible]	[illegible]	[illegible]	[illegible]	[illegible]	[illegible]	[illegible]	[illegible]	[illegible]	[illegible]	[illegible]

Vd991 vs JR2 and VdLs.17															
Scaffold-ID	Star	End	Gene-ID	Type#	Similarity or coverage ratio lower than 50%*	Specific of orthologue clusters*	Locus variation*	JR2	Similarity	Coverage Ratio of JR2	Coverage Ratio of Vd991	VdLs.17	Similarity	Coverage Ratio of VdLs.17	Coverage Ratio of Vd991
[illegible]	[illegible]	[illegible]	[illegible]	[illegible]	[illegible]	[illegible]	[illegible]	[illegible]	[illegible]	[illegible]	[illegible]	[illegible]	[illegible]	[illegible]	[illegible]

JR2 vs Vd991 and VdLs.17															
Chr	Star	End	JR2	Type	Similarity or coverage ratio lower than 50%	Specific of orthologue clusters	Locus variation	Vd991	Similarity	Coverage Ratio of Vd991	Coverage Ratio of JR2	VdLs.17	Similarity	Coverage Ratio of VdLs.17	Coverage Ratio of JR2
[illegible]	[illegible]	[illegible]	[illegible]	[illegible]	[illegible]	[illegible]	[illegible]	[illegible]	[illegible]	[illegible]	[illegible]	[illegible]	[illegible]	[illegible]	[illegible]

VdLs.17 vs Vd991 and JR2																
Chr	Scaffold	Star	End	Gene-ID	Type	Similarity or coverage ratio lower than 50%	Specific of orthologue clusters	Locus variation	Vd991	Similarity	Coverage Ratio of Vd991	Coverage Ratio of VdLs.17	JR2	Similarity	Coverage Ratio of JR2	Coverage Ratio of VdLs.17
[illegible]	[illegible]	[illegible]	[illegible]	[illegible]	[illegible]	[illegible]	[illegible]	[illegible]	[illegible]	[illegible]	[illegible]	[illegible]	[illegible]	[illegible]	[illegible]	[illegible]

Vd991 vs JR2 and VdLs.17																	
Scaffold-ID	Star	End	Gene-ID	Type[a]	Similarity or coverage ratio lower than 50%[b]	Specific of orthologue clusters[c]	Locus variation[d]	JR2	Similarity	Coverage Ratio of JR2	Coverage Ratio of Vd991	VdLs.17	Similarity	Coverage Ratio of VdLs.17	Coverage Ratio of Vd991		
[illegible]	[illegible]	[illegible]	[illegible]	[illegible]	[illegible]	[illegible]	[illegible]	[illegible]	[illegible]	[illegible]	[illegible]	[illegible]	[illegible]	[illegible]	[illegible]		

JR2 vs Vd991 and VdLs.17																
Chr	Star	End	JR2	Type	Similarity or coverage ratio lower than 50%	Specific of orthologue clusters	Locus variation	Vd991	Similarity	Coverage Ratio of Vd991	Coverage Ratio of JR2	VdLs.17	Similarity	Coverage Ratio of VdLs.17	Coverage Ratio of JR2	
[illegible]	[illegible]	[illegible]	[illegible]	[illegible]	[illegible]	[illegible]	[illegible]	[illegible]	[illegible]	[illegible]	[illegible]	[illegible]	[illegible]	[illegible]	[illegible]	

VdLs.17 vs Vd991 and JR2																	
Chr	Scaffold	Star	End	Gene-ID	Type	Similarity or coverage ratio lower than 50%	Specific of orthologue clusters	Locus variation	Vd991	Similarity	Coverage Ratio of Vd991	Coverage Ratio of VdLs.17	JR2	Similarity	Coverage Ratio of JR2	Coverage Ratio of VdLs.17	
[illegible]	[illegible]	[illegible]	[illegible]	[illegible]	[illegible]	[illegible]	[illegible]	[illegible]	[illegible]	[illegible]	[illegible]	[illegible]	[illegible]	[illegible]	[illegible]	[illegible]	

Vd991 vs JR2 and VdLs.17															
Scaffold-ID	Star	End	Gene-ID	Type[a]	Similarity or coverage ratio lower than 50%[b]	Specific of orthologue clusters[c]	Locus variation[d]	JR2	Similarity	Coverage Ratio of JR2	Coverage Ratio of Vd991	VdLs.17	Similarity	Coverage Ratio of VdLs.17	Coverage Ratio of Vd991
[illegible]	[illegible]	[illegible]	[illegible]	[illegible]	[illegible]	[illegible]	[illegible]	[illegible]	[illegible]	[illegible]	[illegible]	[illegible]	[illegible]	[illegible]	[illegible]

JR2 vs Vd991 and VdLs.17															
Chr	Star	End	JR2	Type	Similarity or coverage ratio lower than 50%	Specific of orthologue clusters	Locus variation	Vd991	Similarity	Coverage Ratio of Vd991	Coverage Ratio of JR2	VdLs.17	Similarity	Coverage Ratio of VdLs.17	Coverage Ratio of JR2
[illegible]	[illegible]	[illegible]	[illegible]	[illegible]	[illegible]	[illegible]	[illegible]	[illegible]	[illegible]	[illegible]	[illegible]	[illegible]	[illegible]	[illegible]	[illegible]

VdLs.17 vs Vd991 and JR2																
Chr	Scaffold	Star	End	Gene-ID	Type	Similarity or coverage ratio lower than 50%	Specific of orthologue clusters	Locus variation	Vd991	Similarity	Coverage Ratio of Vd991	Coverage Ratio of VdLs.17	JR2	Similarity	Coverage Ratio of JR2	Coverage Ratio of VdLs.17
[illegible]	[illegible]	[illegible]	[illegible]	[illegible]	[illegible]	[illegible]	[illegible]	[illegible]	[illegible]	[illegible]	[illegible]	[illegible]	[illegible]	[illegible]	[illegible]	[illegible]

Vd991 vs JR2 and VdLs.17															
Scaffold-ID	Star	End	Gene-ID	Type#	Similarity or coverage ratio lower than 50%*	Specific of orthologue clusters*	Locus variation*	JR2	Similarity	Coverage Ratio of JR2	Coverage Ratio of Vd991	VdLs.17	Similarity	Coverage Ratio of VdLs.17	Coverage Ratio of Vd991
[illegible]	[illegible]	[illegible]	[illegible]	[illegible]	[illegible]	[illegible]	[illegible]	[illegible]	[illegible]	[illegible]	[illegible]	[illegible]	[illegible]	[illegible]	[illegible]

JR2 vs Vd991 and VdLs.17															
Chr	Star	End	JR2	Type	Similarity or coverage ratio lower than 50%	Specific of orthologue clusters	Locus variation	Vd991	Similarity	Coverage Ratio of Vd991	Coverage Ratio of JR2	VdLs.17	Similarity	Coverage Ratio of VdLs.17	Coverage Ratio of JR2
[illegible]	[illegible]	[illegible]	[illegible]	[illegible]	[illegible]	[illegible]	[illegible]	[illegible]	[illegible]	[illegible]	[illegible]	[illegible]	[illegible]	[illegible]	[illegible]

VdLs.17 vs Vd991 and JR2																
Chr	Scaffold	Star	End	Gene-ID	Type	Similarity or coverage ratio lower than 50%	Specific of orthologue clusters	Locus variation	Vd991	Similarity	Coverage Ratio of Vd991	Coverage Ratio of VdLs.17	JR2	Similarity	Coverage Ratio of JR2	Coverage Ratio of VdLs.17
[illegible]	[illegible]	[illegible]	[illegible]	[illegible]	[illegible]	[illegible]	[illegible]	[illegible]	[illegible]	[illegible]	[illegible]	[illegible]	[illegible]	[illegible]	[illegible]	[illegible]

Vd991 vs JR2 and VdLs.17																	
Scaffold-ID	Star	End	Gene-ID	Type[a]	Similarity or coverage ratio lower than 50%[*]	Specific of orthologue clusters[*]	Locus variation[*]	JR2	Similarity	Coverage Ratio of JR2	Coverage Ratio of Vd991	VdLs.17	Similarity	Coverage Ratio of VdLs.17	Coverage Ratio of Vd991		
[illegible]	[illegible]	[illegible]	[illegible]	[illegible]	[illegible]	[illegible]	[illegible]	[illegible]	[illegible]	[illegible]	[illegible]	[illegible]	[illegible]	[illegible]	[illegible]		

JR2 vs Vd991 and VdLs.17																
Chr	Star	End	JR2	Type	Similarity or coverage ratio lower than 50%	Specific of orthologue clusters	Locus variation	Vd991	Similarity	Coverage Ratio of Vd991	Coverage Ratio of JR2	VdLs.17	Similarity	Coverage Ratio of VdLs.17	Coverage Ratio of JR2	
[illegible]	[illegible]	[illegible]	[illegible]	[illegible]	[illegible]	[illegible]	[illegible]	[illegible]	[illegible]	[illegible]	[illegible]	[illegible]	[illegible]	[illegible]	[illegible]	

VdLs.17 vs Vd991 and JR2																	
Chr	Scaffold	Star	End	Gene-ID	Type	Similarity or coverage ratio lower than 50%	Specific of orthologue clusters	Locus variation	Vd991	Similarity	Coverage Ratio of Vd991	Coverage Ratio of VdLs.17	JR2	Similarity	Coverage Ratio of JR2	Coverage Ratio of VdLs.17	
[illegible]	[illegible]	[illegible]	[illegible]	[illegible]	[illegible]	[illegible]	[illegible]	[illegible]	[illegible]	[illegible]	[illegible]	[illegible]	[illegible]	[illegible]	[illegible]	[illegible]	

Vd991 vs JR2 and VdLs.17

Scaffold-ID	Star	End	Gene-ID	Type[a]	Similarity or coverage ratio lower than 50%[b]	Specific of orthologue clusters[c]	Locus variation[d]	JR2	Similarity	Coverage Ratio of JR2	Coverage Ratio of Vd991	VdLs.17	Similarity	Coverage Ratio of VdLs.17	Coverage Ratio of Vd991
[illegible]															

JR2 vs Vd991 and VdLs.17

Chr	Star	End	JR2	Type	Similarity or coverage ratio lower than 50%	Specific of orthologue clusters	Locus variation	Vd991	Similarity	Coverage Ratio of Vd991	Coverage Ratio of JR2	VdLs.17	Similarity	Coverage Ratio of VdLs.17	Coverage Ratio of JR2
[illegible]															

VdLs.17 vs Vd991 and JR2

Chr	Scaffold	Star	End	Gene-ID	Type	Similarity or coverage ratio lower than 50%	Specific of orthologue clusters	Locus variation	Vd991	Similarity	Coverage Ratio of Vd991	Coverage Ratio of VdLs.17	JR2	Similarity	Coverage Ratio of JR2	Coverage Ratio of VdLs.17
[illegible]																

Vd991 vs JR2 and VdLs.17															
Scaffold-ID	Star	End	Gene-ID	Type[a]	Similarity or coverage ratio lower than 50%[*]	Specific of orthologue clusters[*]	Locus variation[*]	JR2	Similarity	Coverage Ratio of JR2	Coverage Ratio of Vd991	VdLs.17	Similarity	Coverage Ratio of VdLs.17	Coverage Ratio of Vd991
[illegible]															

JR2 vs Vd991 and VdLs.17																
Chr	Star	End	JR2	Type	Similarity or coverage ratio lower than 50%	Specific of orthologue clusters	Locus variation	Vd991	Similarity	Coverage Ratio of Vd991	Coverage Ratio of JR2	VdLs.17	Similarity	Coverage Ratio of VdLs.17	Coverage Ratio of JR2
[illegible]																

VdLs.17 vs Vd991 and JR2																	
Chr	Scaffold	Star	End	Gene-ID	Type	Similarity or coverage ratio lower than 50%	Specific of orthologue clusters	Locus variation	Vd991	Similarity	Coverage Ratio of Vd991	Coverage Ratio of VdLs.17	JR2	Similarity	Coverage Ratio of JR2	Coverage Ratio of VdLs.17
[illegible]																	

Vd991 vs JR2 and VdLs.17

Scaffold-ID	Star	End	Gene-ID	Type[a]	Similarity or coverage ratio lower than 50%[*]	Specific of orthologue clusters[*]	Locus variation[*]	JR2	Similarity	Coverage Ratio of JR2	Coverage Ratio of Vd991	VdLs.17	Similarity	Coverage Ratio of VdLs.17	Coverage Ratio of Vd991
[illegible]	[illegible]	[illegible]	[illegible]	[illegible]	[illegible]	[illegible]	[illegible]	[illegible]	[illegible]	[illegible]	[illegible]	[illegible]	[illegible]	[illegible]	[illegible]

JR2 vs Vd991 and VdLs.17

Chr	Star	End	JR2	Type	Similarity or coverage ratio lower than 50%	Specific of orthologue clusters	Locus variation	Vd991	Similarity	Coverage Ratio of Vd991	Coverage Ratio of JR2	VdLs.17	Similarity	Coverage Ratio of VdLs.17	Coverage Ratio of JR2
[illegible]	[illegible]	[illegible]	[illegible]	[illegible]	[illegible]	[illegible]	[illegible]	[illegible]	[illegible]	[illegible]	[illegible]	[illegible]	[illegible]	[illegible]	[illegible]

VdLs.17 vs Vd991 and JR2

Chr	Scaffold	Star	End	Gene-ID	Type	Similarity or coverage ratio lower than 50%	Specific of orthologue clusters	Locus variation	Vd991	Similarity	Coverage Ratio of Vd991	Coverage Ratio of VdLs.17	JR2	Similarity	Coverage Ratio of JR2	Coverage Ratio of VdLs.17
[illegible]	[illegible]	[illegible]	[illegible]	[illegible]	[illegible]	[illegible]	[illegible]	[illegible]	[illegible]	[illegible]	[illegible]	[illegible]	[illegible]	[illegible]	[illegible]	[illegible]

Vd991 vs JR2 and VdLs.17															
Scaffold-ID	Star	End	Gene-ID	Type[a]	Similarity or coverage ratio lower than 50%[b]	Specific of orthologue clusters[c]	Locus variation[d]	JR2	Similarity	Coverage Ratio of JR2	Coverage Ratio of Vd991	VdLs.17	Similarity	Coverage Ratio of VdLs.17	Coverage Ratio of Vd991
[illegible]	[illegible]	[illegible]	[illegible]	[illegible]	[illegible]	[illegible]	[illegible]	[illegible]	[illegible]	[illegible]	[illegible]	[illegible]	[illegible]	[illegible]	[illegible]

JR2 vs Vd991 and VdLs.17															
Chr	Star	End	JR2	Type	Similarity or coverage ratio lower than 50%	Specific of orthologue clusters	Locus variation	Vd991	Similarity	Coverage Ratio of Vd991	Coverage Ratio of JR2	VdLs.17	Similarity	Coverage Ratio of VdLs.17	Coverage Ratio of JR2
[illegible]	[illegible]	[illegible]	[illegible]	[illegible]	[illegible]	[illegible]	[illegible]	[illegible]	[illegible]	[illegible]	[illegible]	[illegible]	[illegible]	[illegible]	[illegible]

VdLs.17 vs Vd991 and JR2																
Chr	Scaffold	Star	End	Gene-ID	Type	Similarity or coverage ratio lower than 50%	Specific of orthologue clusters	Locus variation	Vd991	Similarity	Coverage Ratio of Vd991	Coverage Ratio of VdLs.17	JR2	Similarity	Coverage Ratio of JR2	Coverage Ratio of VdLs.17
[illegible]	[illegible]	[illegible]	[illegible]	[illegible]	[illegible]	[illegible]	[illegible]	[illegible]	[illegible]	[illegible]	[illegible]	[illegible]	[illegible]	[illegible]	[illegible]	[illegible]

Vd991 vs JR2 and VdLs.17																	
Scaffold-ID	Star	End	Gene-ID	Type[a]	Similarity or coverage ratio lower than 50%[*]	Specific of orthologue clusters[*]	Locus variation[*]	JR2	Similarity	Coverage Ratio of JR2	Coverage Ratio of Vd991	VdLs.17	Similarity	Coverage Ratio of VdLs.17	Coverage Ratio of Vd991		
[illegible]																	

JR2 vs Vd991 and VdLs.17																	
Chr	Star	End	JR2	Type	Similarity or coverage ratio lower than 50%	Specific of orthologue clusters	Locus variation	Vd991	Similarity	Coverage Ratio of Vd991	Coverage Ratio of JR2	VdLs.17	Similarity	Coverage Ratio of VdLs.17	Coverage Ratio of JR2		
[illegible]																	

VdLs.17 vs Vd991 and JR2																	
Chr	Scaffold	Star	End	Gene-ID	Type	Similarity or coverage ratio lower than 50%	Specific of orthologue clusters	Locus variation	Vd991	Similarity	Coverage Ratio of Vd991	Coverage Ratio of VdLs.17	JR2	Similarity	Coverage Ratio of JR2	Coverage Ratio of VdLs.17	
[illegible]																	

Vd991 vs JR2 and VdLs.17															
Scaffold-ID	Star	End	Gene-ID	Type#	Similarity or coverage ratio lower than 50%*	Specific of orthologue clusters*	Locus variation*	JR2	Similarity	Coverage Ratio of JR2	Coverage Ratio of Vd991	VdLs.17	Similarity	Coverage Ratio of VdLs.17	Coverage Ratio of Vd991
[illegible]	[illegible]	[illegible]	[illegible]	[illegible]	[illegible]	[illegible]	[illegible]	[illegible]	[illegible]	[illegible]	[illegible]	[illegible]	[illegible]	[illegible]	[illegible]

JR2 vs Vd991 and VdLs.17															
Chr	Star	End	JR2	Type	Similarity or coverage ratio lower than 50%	Specific of orthologue clusters	Locus variation	Vd991	Similarity	Coverage Ratio of Vd991	Coverage Ratio of JR2	VdLs.17	Similarity	Coverage Ratio of VdLs.17	Coverage Ratio of JR2
[illegible]	[illegible]	[illegible]	[illegible]	[illegible]	[illegible]	[illegible]	[illegible]	[illegible]	[illegible]	[illegible]	[illegible]	[illegible]	[illegible]	[illegible]	[illegible]

VdLs.17 vs Vd991 and JR2																
Chr	Scaffold	Star	End	Gene-ID	Type	Similarity or coverage ratio lower than 50%	Specific of orthologue clusters	Locus variation	Vd991	Similarity	Coverage Ratio of Vd991	Coverage Ratio of VdLs.17	JR2	Similarity	Coverage Ratio of JR2	Coverage Ratio of VdLs.17
[illegible]	[illegible]	[illegible]	[illegible]	[illegible]	[illegible]	[illegible]	[illegible]	[illegible]	[illegible]	[illegible]	[illegible]	[illegible]	[illegible]	[illegible]	[illegible]	[illegible]

Vd991 vs JR2 and VdLs.17																	
Scaffold-ID	Star	End	Gene-ID	Type[a]	Similarity or coverage ratio lower than 50%[*]	Specific of orthologue clusters[*]	Locus variation[*]	JR2	Similarity	Coverage Ratio of JR2	Coverage Ratio of Vd991	VdLs.17	Similarity	Coverage Ratio of VdLs.17	Coverage Ratio of Vd991		
[illegible]	[illegible]	[illegible]	[illegible]	[illegible]	[illegible]	[illegible]	[illegible]	[illegible]	[illegible]	[illegible]	[illegible]	[illegible]	[illegible]	[illegible]	[illegible]		

JR2 vs Vd991 and VdLs.17																
Chr	Star	End	JR2	Type	Similarity or coverage ratio lower than 50%	Specific of orthologue clusters	Locus variation	Vd991	Similarity	Coverage Ratio of Vd991	Coverage Ratio of JR2	VdLs.17	Similarity	Coverage Ratio of VdLs.17	Coverage Ratio of JR2	
[illegible]	[illegible]	[illegible]	[illegible]	[illegible]	[illegible]	[illegible]	[illegible]	[illegible]	[illegible]	[illegible]	[illegible]	[illegible]	[illegible]	[illegible]	[illegible]	

VdLs.17 vs Vd991 and JR2																	
Chr	Scaffold	Star	End	Gene-ID	Type	Similarity or coverage ratio lower than 50%	Specific of orthologue clusters	Locus variation	Vd991	Similarity	Coverage Ratio of Vd991	Coverage Ratio of VdLs.17	JR2	Similarity	Coverage Ratio of JR2	Coverage Ratio of VdLs.17	
[illegible]	[illegible]	[illegible]	[illegible]	[illegible]	[illegible]	[illegible]	[illegible]	[illegible]	[illegible]	[illegible]	[illegible]	[illegible]	[illegible]	[illegible]	[illegible]	[illegible]	

Vd991 vs JR2 and VdLs.17

Scaffold-ID	Star	End	Gene-ID	Type[a]	Similarity or coverage ratio lower than 50%[b]	Specific of orthologue clusters[c]	Locus variation[d]	JR2	Similarity	Coverage Ratio of JR2	Coverage Ratio of Vd991	VdLs.17	Similarity	Coverage Ratio of VdLs.17	Coverage Ratio of Vd991
[illegible]															

JR2 vs Vd991 and VdLs.17

Chr	Star	End	JR2	Type	Similarity or coverage ratio lower than 50%	Specific of orthologue clusters	Locus variation	Vd991	Similarity	Coverage Ratio of Vd991	Coverage Ratio of JR2	VdLs.17	Similarity	Coverage Ratio of VdLs.17	Coverage Ratio of JR2
[illegible]															

VdLs.17 vs Vd991 and JR2

Chr	Scaffold	Star	End	Gene-ID	Type	Similarity or coverage ratio lower than 50%	Specific of orthologue clusters	Locus variation	Vd991	Similarity	Coverage Ratio of Vd991	Coverage Ratio of VdLs.17	JR2	Similarity	Coverage Ratio of JR2	Coverage Ratio of VdLs.17
[illegible]																

Vd991 vs JR2 and VdLs.17															
Scaffold-ID	Star	End	Gene-ID	Type[a]	Similarity or coverage ratio lower than 50%[b]	Specific of orthologue clusters[c]	Locus variation[d]	JR2	Similarity	Coverage Ratio of JR2	Coverage Ratio of Vd991	VdLs.17	Similarity	Coverage Ratio of VdLs.17	Coverage Ratio of Vd991
[illegible]	[illegible]	[illegible]	[illegible]	[illegible]	[illegible]	[illegible]	[illegible]	[illegible]	[illegible]	[illegible]	[illegible]	[illegible]	[illegible]	[illegible]	[illegible]

JR2 vs Vd991 and VdLs.17															
Chr	Star	End	JR2	Type	Similarity or coverage ratio lower than 50%	Specific of orthologue clusters	Locus variation	Vd991	Similarity	Coverage Ratio of Vd991	Coverage Ratio of JR2	VdLs.17	Similarity	Coverage Ratio of VdLs.17	Coverage Ratio of JR2
[illegible]	[illegible]	[illegible]	[illegible]	[illegible]	[illegible]	[illegible]	[illegible]	[illegible]	[illegible]	[illegible]	[illegible]	[illegible]	[illegible]	[illegible]	[illegible]

VdLs.17 vs Vd991 and JR2																
Chr	Scaffold	Star	End	Gene-ID	Type	Similarity or coverage ratio lower than 50%	Specific of orthologue clusters	Locus variation	Vd991	Similarity	Coverage Ratio of Vd991	Coverage Ratio of VdLs.17	JR2	Similarity	Coverage Ratio of JR2	Coverage Ratio of VdLs.17
[illegible]	[illegible]	[illegible]	[illegible]	[illegible]	[illegible]	[illegible]	[illegible]	[illegible]	[illegible]	[illegible]	[illegible]	[illegible]	[illegible]	[illegible]	[illegible]	[illegible]

Vd991 vs JR2 and VdLs.17															
Scaffold-ID	Star	End	Gene-ID	Type[a]	Similarity or coverage ratio lower than 50%[b]	Specific of orthologue clusters[c]	Locus variation[d]	JR2	Similarity	Coverage Ratio of JR2	Coverage Ratio of Vd991	VdLs.17	Similarity	Coverage Ratio of VdLs.17	Coverage Ratio of Vd991
[illegible]	[illegible]	[illegible]	[illegible]	[illegible]	[illegible]	[illegible]	[illegible]	[illegible]	[illegible]	[illegible]	[illegible]	[illegible]	[illegible]	[illegible]	[illegible]

JR2 vs Vd991 and VdLs.17															
Chr	Star	End	JR2	Type	Similarity or coverage ratio lower than 50%	Specific of orthologue clusters	Locus variation	Vd991	Similarity	Coverage Ratio of Vd991	Coverage Ratio of JR2	VdLs.17	Similarity	Coverage Ratio of VdLs.17	Coverage Ratio of JR2
[illegible]	[illegible]	[illegible]	[illegible]	[illegible]	[illegible]	[illegible]	[illegible]	[illegible]	[illegible]	[illegible]	[illegible]	[illegible]	[illegible]	[illegible]	[illegible]

VdLs.17 vs Vd991 and JR2																
Chr	Scaffold	Star	End	Gene-ID	Type	Similarity or coverage ratio lower than 50%	Specific of orthologue clusters	Locus variation	Vd991	Similarity	Coverage Ratio of Vd991	Coverage Ratio of VdLs.17	JR2	Similarity	Coverage Ratio of JR2	Coverage Ratio of VdLs.17
[illegible]	[illegible]	[illegible]	[illegible]	[illegible]	[illegible]	[illegible]	[illegible]	[illegible]	[illegible]	[illegible]	[illegible]	[illegible]	[illegible]	[illegible]	[illegible]	[illegible]

Vd991 vs JR2 and VdLs.17

Scaffold-ID	Star	End	Gene-ID	Type#	Similarity or coverage ratio lower than 50%*	Specific of orthologue clusters*	Locus variation*	JR2	Similarity	Coverage Ratio of JR2	Coverage Ratio of Vd991	VdLs.17	Similarity	Coverage Ratio of VdLs.17	Coverage Ratio of Vd991
[illegible]	[illegible]	[illegible]	[illegible]	[illegible]	[illegible]	[illegible]	[illegible]	[illegible]	[illegible]	[illegible]	[illegible]	[illegible]	[illegible]	[illegible]	[illegible]

JR2 vs Vd991 and VdLs.17

Chr	Star	End	JR2	Type	Similarity or coverage ratio lower than 50%	Specific of orthologue clusters	Locus variation	Vd991	Similarity	Coverage Ratio of Vd991	Coverage Ratio of JR2	VdLs.17	Similarity	Coverage Ratio of VdLs.17	Coverage Ratio of JR2
[illegible]	[illegible]	[illegible]	[illegible]	[illegible]	[illegible]	[illegible]	[illegible]	[illegible]	[illegible]	[illegible]	[illegible]	[illegible]	[illegible]	[illegible]	[illegible]

VdLs.17 vs Vd991 and JR2

Chr	Scaffold	Star	End	Gene-ID	Type	Similarity or coverage ratio lower than 50%	Specific of orthologue clusters	Locus variation	Vd991	Similarity	Coverage Ratio of Vd991	Coverage Ratio of VdLs.17	JR2	Similarity	Coverage Ratio of JR2	Coverage Ratio of VdLs.17
[illegible]	[illegible]	[illegible]	[illegible]	[illegible]	[illegible]	[illegible]	[illegible]	[illegible]	[illegible]	[illegible]	[illegible]	[illegible]	[illegible]	[illegible]	[illegible]	[illegible]

Scaffold-ID	Star	End	Gene-ID	Type#	Similarity or coverage ratio lower than 50%*	Specific of orthologue clusters*	Locus variation*	Vd991 vs JR2 and VdLs.17: JR2	Similarity	Coverage Ratio of JR2	Coverage Ratio of Vd991	VdLs.17	Similarity	Coverage Ratio of VdLs.17	Coverage Ratio of Vd991
[illegible]	[illegible]	[illegible]	[illegible]	[illegible]	[illegible]	[illegible]	[illegible]	[illegible]	[illegible]	[illegible]	[illegible]	[illegible]	[illegible]	[illegible]	[illegible]

Chr	Star	End	JR2	Type	Similarity or coverage ratio lower than 50%	Specific of orthologue clusters	Locus variation	JR2 vs Vd991 and VdLs.17: Vd991	Similarity	Coverage Ratio of Vd991	Coverage Ratio of JR2	VdLs.17	Similarity	Coverage Ratio of VdLs.17	Coverage Ratio of JR2
[illegible]	[illegible]	[illegible]	[illegible]	[illegible]	[illegible]	[illegible]	[illegible]	[illegible]	[illegible]	[illegible]	[illegible]	[illegible]	[illegible]	[illegible]	[illegible]

Chr	Scaffold	Star	End	Gene-ID	Type	Similarity or coverage ratio lower than 50%	Specific of orthologue clusters	Locus variation	VdLs.17 vs Vd991 and JR2: Vd991	Similarity	Coverage Ratio of Vd991	Coverage Ratio of VdLs.17	JR2	Similarity	Coverage Ratio of JR2	Coverage Ratio of VdLs.17
[illegible]	[illegible]	[illegible]	[illegible]	[illegible]	[illegible]	[illegible]	[illegible]	[illegible]	[illegible]	[illegible]	[illegible]	[illegible]	[illegible]	[illegible]	[illegible]	[illegible]

Vd991 vs JR2 and VdLs.17																JR2 vs Vd991 and VdLs.17																VdLs.17 vs Vd991 and JR2																
Scaffold-ID	Star	End	Gene-ID	Type#	Similarity or coverage ratio lower than 50%*	Specific of orthologue clusters*	Locus variation*	JR2	Similarity	Coverage Ratio of JR2	Coverage Ratio of Vd991	VdLs.17	Similarity	Coverage Ratio of VdLs.17	Coverage Ratio of Vd991	Chr	Star	End	JR2	Type	Similarity or coverage ratio lower than 50%	Specific of orthologue clusters	Locus variation	Vd991	Similarity	Coverage Ratio of Vd991	Coverage Ratio of JR2	VdLs.17	Similarity	Coverage Ratio of VdLs.17	Coverage Ratio of JR2	Chr	Scaffold	Star	End	Gene-ID	Type	Similarity or coverage ratio lower than 50%	Specific of orthologue clusters	Locus variation	Vd991	Similarity	Coverage Ratio of Vd991	Coverage Ratio of VdLs.17	JR2	Similarity	Coverage Ratio of JR2	Coverage Ratio of VdLs.17
[illegible]	[illegible]	[illegible]	[illegible]	[illegible]	[illegible]	[illegible]	[illegible]	[illegible]	[illegible]	[illegible]	[illegible]	[illegible]	[illegible]	[illegible]	[illegible]	[illegible]	[illegible]	[illegible]	[illegible]	[illegible]	[illegible]	[illegible]	[illegible]	[illegible]	[illegible]	[illegible]	[illegible]	[illegible]	[illegible]	[illegible]	[illegible]	[illegible]	[illegible]	[illegible]	[illegible]	[illegible]	[illegible]	[illegible]	[illegible]	[illegible]	[illegible]	[illegible]	[illegible]	[illegible]	[illegible]	[illegible]	[illegible]	[illegible]

Vd991 vs JR2 and VdLs.17

Scaffold-ID	Star	End	Gene-ID	Type[a]	Similarity or coverage ratio lower than 50%[*]	Specific of orthologue clusters[*]	Locus variation[*]	JR2	Similarity	Coverage Ratio of JR2	Coverage Ratio of Vd991	VdLs.17	Similarity	Coverage Ratio of VdLs.17	Coverage Ratio of Vd991
[illegible]	[illegible]	[illegible]	[illegible]	[illegible]	[illegible]	[illegible]	[illegible]	[illegible]	[illegible]	[illegible]	[illegible]	[illegible]	[illegible]	[illegible]	[illegible]

JR2 vs Vd991 and VdLs.17

Chr	Star	End	JR2	Type	Similarity or coverage ratio lower than 50%	Specific of orthologue clusters	Locus variation	Vd991	Similarity	Coverage Ratio of Vd991	Coverage Ratio of JR2	VdLs.17	Similarity	Coverage Ratio of VdLs.17	Coverage Ratio of JR2
[illegible]	[illegible]	[illegible]	[illegible]	[illegible]	[illegible]	[illegible]	[illegible]	[illegible]	[illegible]	[illegible]	[illegible]	[illegible]	[illegible]	[illegible]	[illegible]

VdLs.17 vs Vd991 and JR2

Chr	Scaffold	Star	End	Gene-ID	Type	Similarity or coverage ratio lower than 50%	Specific of orthologue clusters	Locus variation	Vd991	Similarity	Coverage Ratio of Vd991	Coverage Ratio of VdLs.17	JR2	Similarity	Coverage Ratio of JR2	Coverage Ratio of VdLs.17
[illegible]	[illegible]	[illegible]	[illegible]	[illegible]	[illegible]	[illegible]	[illegible]	[illegible]	[illegible]	[illegible]	[illegible]	[illegible]	[illegible]	[illegible]	[illegible]	[illegible]

Vd991 vs JR2 and VdLs.17																	
Scaffold-ID	Star	End	Gene-ID	Type[a]	Similarity or coverage ratio lower than 50%[b]	Specific of orthologue clusters[c]	Locus variation[d]	JR2	Similarity	Coverage Ratio of JR2	Coverage Ratio of Vd991	VdLs.17	Similarity	Coverage Ratio of VdLs.17	Coverage Ratio of Vd991		
[illegible]	[illegible]	[illegible]	[illegible]	[illegible]	[illegible]	[illegible]	[illegible]	[illegible]	[illegible]	[illegible]	[illegible]	[illegible]	[illegible]	[illegible]	[illegible]		

JR2 vs Vd991 and VdLs.17															
Chr	Star	End	JR2	Type	Similarity or coverage ratio lower than 50%	Specific of orthologue clusters	Locus variation	Vd991	Similarity	Coverage Ratio of Vd991	Coverage Ratio of JR2	VdLs.17	Similarity	Coverage Ratio of VdLs.17	Coverage Ratio of JR2
[illegible]	[illegible]	[illegible]	[illegible]	[illegible]	[illegible]	[illegible]	[illegible]	[illegible]	[illegible]	[illegible]	[illegible]	[illegible]	[illegible]	[illegible]	[illegible]

VdLs.17 vs Vd991 and JR2																
Chr	Scaffold	Star	End	Gene-ID	Type	Similarity or coverage ratio lower than 50%	Specific of orthologue clusters	Locus variation	Vd991	Similarity	Coverage Ratio of Vd991	Coverage Ratio of VdLs.17	JR2	Similarity	Coverage Ratio of JR2	Coverage Ratio of VdLs.17
[illegible]	[illegible]	[illegible]	[illegible]	[illegible]	[illegible]	[illegible]	[illegible]	[illegible]	[illegible]	[illegible]	[illegible]	[illegible]	[illegible]	[illegible]	[illegible]	[illegible]

Vd991 vs JR2 and VdLs.17															
Scaffold-ID	Star	End	Gene-ID	Type[#]	Similarity or coverage ratio lower than 50%[*]	Specific of orthologue clusters[*]	Locus variation[*]	JR2	Similarity	Coverage Ratio of JR2	Coverage Ratio of Vd991	VdLs.17	Similarity	Coverage Ratio of VdLs.17	Coverage Ratio of Vd991
[illegible]	[illegible]	[illegible]	[illegible]	[illegible]	[illegible]	[illegible]	[illegible]	[illegible]	[illegible]	[illegible]	[illegible]	[illegible]	[illegible]	[illegible]	[illegible]

JR2 vs Vd991 and VdLs.17															
Chr	Star	End	JR2	Type	Similarity or coverage ratio lower than 50%	Specific of orthologue clusters	Locus variation	Vd991	Similarity	Coverage Ratio of Vd991	Coverage Ratio of JR2	VdLs.17	Similarity	Coverage Ratio of VdLs.17	Coverage Ratio of JR2
[illegible]	[illegible]	[illegible]	[illegible]	[illegible]	[illegible]	[illegible]	[illegible]	[illegible]	[illegible]	[illegible]	[illegible]	[illegible]	[illegible]	[illegible]	[illegible]

VdLs.17 vs Vd991 and JR2																
Chr	Scaffold	Star	End	Gene-ID	Type	Similarity or coverage ratio lower than 50%	Specific of orthologue clusters	Locus variation	Vd991	Similarity	Coverage Ratio of Vd991	Coverage Ratio of VdLs.17	JR2	Similarity	Coverage Ratio of JR2	Coverage Ratio of VdLs.17
[illegible]	[illegible]	[illegible]	[illegible]	[illegible]	[illegible]	[illegible]	[illegible]	[illegible]	[illegible]	[illegible]	[illegible]	[illegible]	[illegible]	[illegible]	[illegible]	[illegible]

Vd991 vs JR2 and VdLs.17															
Scaffold-ID	Star	End	Gene-ID	Type#	Similarity or coverage ratio lower than 50%*	Specific of orthologue clusters*	Locus variation*	JR2	Similarity	Coverage Ratio of JR2	Coverage Ratio of Vd991	VdLs.17	Similarity	Coverage Ratio of VdLs.17	Coverage Ratio of Vd991
[illegible]	[illegible]	[illegible]	[illegible]	[illegible]	[illegible]	[illegible]	[illegible]	[illegible]	[illegible]	[illegible]	[illegible]	[illegible]	[illegible]	[illegible]	[illegible]

JR2 vs Vd991 and VdLs.17															
Chr	Star	End	JR2	Type	Similarity or coverage ratio lower than 50%	Specific of orthologue clusters	Locus variation	Vd991	Similarity	Coverage Ratio of Vd991	Coverage Ratio of JR2	VdLs.17	Similarity	Coverage Ratio of VdLs.17	Coverage Ratio of JR2
[illegible]	[illegible]	[illegible]	[illegible]	[illegible]	[illegible]	[illegible]	[illegible]	[illegible]	[illegible]	[illegible]	[illegible]	[illegible]	[illegible]	[illegible]	[illegible]

VdLs.17 vs Vd991 and JR2																
Chr	Scaffold	Star	End	Gene-ID	Type	Similarity or coverage ratio lower than 50%	Specific of orthologue clusters	Locus variation	Vd991	Similarity	Coverage Ratio of Vd991	Coverage Ratio of VdLs.17	JR2	Similarity	Coverage Ratio of JR2	Coverage Ratio of VdLs.17
[illegible]	[illegible]	[illegible]	[illegible]	[illegible]	[illegible]	[illegible]	[illegible]	[illegible]	[illegible]	[illegible]	[illegible]	[illegible]	[illegible]	[illegible]	[illegible]	[illegible

Vd991 vs JR2 and VdLs.17															
Scaffold-ID	Star	End	Gene-ID	Type#	Similarity or coverage ratio lower than 50%*	Specific of orthologue clusters*	Locus variation*	JR2	Similarity	Coverage Ratio of JR2	Coverage Ratio of Vd991	VdLs.17	Similarity	Coverage Ratio of VdLs.17	Coverage Ratio of Vd991
[illegible]	[illegible]	[illegible]	[illegible]	[illegible]	[illegible]	[illegible]	[illegible]	[illegible]	[illegible]	[illegible]	[illegible]	[illegible]	[illegible]	[illegible]	[illegible]

JR2 vs Vd991 and VdLs.17															
Chr	Star	End	JR2	Type	Similarity or coverage ratio lower than 50%	Specific of orthologue clusters	Locus variation	Vd991	Similarity	Coverage Ratio of Vd991	Coverage Ratio of JR2	VdLs.17	Similarity	Coverage Ratio of VdLs.17	Coverage Ratio of JR2
[illegible]	[illegible]	[illegible]	[illegible]	[illegible]	[illegible]	[illegible]	[illegible]	[illegible]	[illegible]	[illegible]	[illegible]	[illegible]	[illegible]	[illegible]	[illegible]

VdLs.17 vs Vd991 and JR2																
Chr	Scaffold	Star	End	Gene-ID	Type	Similarity or coverage ratio lower than 50%	Specific of orthologue clusters	Locus variation	Vd991	Similarity	Coverage Ratio of Vd991	Coverage Ratio of VdLs.17	JR2	Similarity	Coverage Ratio of JR2	Coverage Ratio of VdLs.17
[illegible]	[illegible]	[illegible]	[illegible]	[illegible]	[illegible]	[illegible]	[illegible]	[illegible]	[illegible]	[illegible]	[illegible]	[illegible]	[illegible]	[illegible]	[illegible]	[illegible]

Scaffold-ID	Star	End	Gene-ID	Type[a]	Similarity or coverage ratio lower than 50%[*]	Specific of orthologue clusters[*]	Locus variation[*]	Vd991 vs JR2 and VdLs.17							
								JR2	Similarity	Coverage Ratio of JR2	Coverage Ratio of Vd991	VdLs.17	Similarity	Coverage Ratio of VdLs.17	Coverage Ratio of Vd991
[illegible]	[illegible]	[illegible]	[illegible]	[illegible]	[illegible]	[illegible]	[illegible]	[illegible]	[illegible]	[illegible]	[illegible]	[illegible]	[illegible]	[illegible]	[illegible]

Chr	Star	End	JR2	Type	Similarity or coverage ratio lower than 50%	Specific of orthologue clusters	Locus variation	JR2 vs Vd991 and VdLs.17							
								Vd991	Similarity	Coverage Ratio of Vd991	Coverage Ratio of JR2	VdLs.17	Similarity	Coverage Ratio of VdLs.17	Coverage Ratio of JR2
[illegible]	[illegible]	[illegible]	[illegible]	[illegible]	[illegible]	[illegible]	[illegible]	[illegible]	[illegible]	[illegible]	[illegible]	[illegible]	[illegible]	[illegible]	[illegible]

Chr	Scaffold	Star	End	Gene-ID	Type	Similarity or coverage ratio lower than 50%	Specific of orthologue clusters	Locus variation	VdLs.17 vs Vd991 and JR2							
									Vd991	Similarity	Coverage Ratio of Vd991	Coverage Ratio of VdLs.17	JR2	Similarity	Coverage Ratio of JR2	Coverage Ratio of VdLs.17
[illegible]	[illegible]	[illegible]	[illegible]	[illegible]	[illegible]	[illegible]	[illegible]	[illegible]	[illegible]	[illegible]	[illegible]	[illegible]	[illegible]	[illegible]	[illegible]	[illegible]

Vd991 vs JR2 and VdLs.17															
Scaffold-ID	Star	End	Gene-ID	Type#	Similarity or coverage ratio lower than 50%*	Specific of orthologue clusters*	Locus variation*	JR2	Similarity	Coverage Ratio of JR2	Coverage Ratio of Vd991	VdLs.17	Similarity	Coverage Ratio of VdLs.17	Coverage Ratio of Vd991
[illegible]	[illegible]	[illegible]	[illegible]	[illegible]	[illegible]	[illegible]	[illegible]	[illegible]	[illegible]	[illegible]	[illegible]	[illegible]	[illegible]	[illegible]	[illegible]

JR2 vs Vd991 and VdLs.17															
Chr	Star	End	JR2	Type	Similarity or coverage ratio lower than 50%	Specific of orthologue clusters	Locus variation	Vd991	Similarity	Coverage Ratio of Vd991	Coverage Ratio of JR2	VdLs.17	Similarity	Coverage Ratio of VdLs.17	Coverage Ratio of JR2
[illegible]	[illegible]	[illegible]	[illegible]	[illegible]	[illegible]	[illegible]	[illegible]	[illegible]	[illegible]	[illegible]	[illegible]	[illegible]	[illegible]	[illegible]	[illegible]

VdLs.17 vs Vd991 and JR2																
Chr	Scaffold	Star	End	Gene-ID	Type	Similarity or coverage ratio lower than 50%	Specific of orthologue clusters	Locus variation	Vd991	Similarity	Coverage Ratio of Vd991	Coverage Ratio of VdLs.17	JR2	Similarity	Coverage Ratio of JR2	Coverage Ratio of VdLs.17
[illegible]	[illegible]	[illegible]	[illegible]	[illegible]	[illegible]	[illegible]	[illegible]	[illegible]	[illegible]	[illegible]	[illegible]	[illegible]	[illegible]	[illegible]	[illegible]	[illegible]

Vd991 vs JR2 and VdLs.17

Scaffold-ID	Star	End	Gene-ID	Type[a]	Similarity or coverage ratio lower than 50%[b]	Specific of orthologue clusters[c]	Locus variation[d]	JR2	Similarity	Coverage Ratio of JR2	Coverage Ratio of Vd991	VdLs.17	Similarity	Coverage Ratio of VdLs.17	Coverage Ratio of Vd991
[illegible]	[illegible]	[illegible]	[illegible]	[illegible]	[illegible]	[illegible]	[illegible]	[illegible]	[illegible]	[illegible]	[illegible]	[illegible]	[illegible]	[illegible]	[illegible]

JR2 vs Vd991 and VdLs.17

Chr	Star	End	JR2	Type	Similarity or coverage ratio lower than 50%	Specific of orthologue clusters	Locus variation	Vd991	Similarity	Coverage Ratio of Vd991	Coverage Ratio of JR2	VdLs.17	Similarity	Coverage Ratio of VdLs.17	Coverage Ratio of JR2
[illegible]	[illegible]	[illegible]	[illegible]	[illegible]	[illegible]	[illegible]	[illegible]	[illegible]	[illegible]	[illegible]	[illegible]	[illegible]	[illegible]	[illegible]	[illegible]

VdLs.17 vs Vd991 and JR2

Chr	Scaffold	Star	End	Gene-ID	Type	Similarity or coverage ratio lower than 50%	Specific of orthologue clusters	Locus variation	Vd991	Similarity	Coverage Ratio of Vd991	Coverage Ratio of VdLs.17	JR2	Similarity	Coverage Ratio of JR2	Coverage Ratio of VdLs.17
[illegible]	[illegible]	[illegible]	[illegible]	[illegible]	[illegible]	[illegible]	[illegible]	[illegible]	[illegible]	[illegible]	[illegible]	[illegible]	[illegible]	[illegible]	[illegible]	[illegible]

Vd991 vs JR2 and VdLs.17																	
Scaffold-ID	Star	End	Gene-ID	Type[a]	Similarity or coverage ratio lower than 50%[b]	Specific of orthologue clusters[c]	Locus variation[d]	JR2	Similarity	Coverage Ratio of JR2	Coverage Ratio of Vd991	VdLs.17	Similarity	Coverage Ratio of VdLs.17	Coverage Ratio of Vd991		
[illegible]																	

JR2 vs Vd991 and VdLs.17																
Chr	Star	End	JR2	Type	Similarity or coverage ratio lower than 50%	Specific of orthologue clusters	Locus variation	Vd991	Similarity	Coverage Ratio of Vd991	Coverage Ratio of JR2	VdLs.17	Similarity	Coverage Ratio of VdLs.17	Coverage Ratio of JR2	
[illegible]																

VdLs.17 vs Vd991 and JR2																	
Chr	Scaffold	Star	End	Gene-ID	Type	Similarity or coverage ratio lower than 50%	Specific of orthologue clusters	Locus variation	Vd991	Similarity	Coverage Ratio of Vd991	Coverage Ratio of VdLs.17	JR2	Similarity	Coverage Ratio of JR2	Coverage Ratio of VdLs.17	
[illegible]																	

[illegible]
