# Supplementary material for: Comparative genomics reveals cotton‐specific virulence factors in flexible genomic regions in Verticillium dahliae and evidence of horizontal gene transfer from Fusarium
Source: New Phytol. 2017 Oct 30;217(2):756–70. doi: 10.1111/nph.14861 (PMC5765495; doi:10.1111/nph.14861)
Supplement: Supplementary file 3 [file NPH-217-756-s003.pdf]

Table S18. Annotations of the protein-coding genes within LSRs among the three genomes of *Verticillium dahliae* isolates Vd991, JR2 and VdLs.17

Scaffold:position	Gene-ID	Pathogen	Secretome	LysM	NLP	SCRPs	CAZymes	Pectin degradation	Cellulose degradation	Hemicellulose degradation	Lignin degradation	PHI_define	Kinase	Transcription factor	eggNOG_ID	eggNOG_class	eggNOG_define	IPR_define	KO_ID	KEGG_Third	BR_family/PATH_way	Responsed to cotton	Responsed to tomato	Responsed to lettuce
**Vd991 LSRs**																								
**G-LSR1 (32 genes)**																								
Scaffold24:428946-431306	VEDA_04892																							
Scaffold24:431809-433728	VEDA_04893														fuNOG03080	A	Protein involved in mRNA metab	IPR006568; PSP, proline-rich//IPR007180; I	K12829	Spliceosome	[PATH:ko03040][BR:ko03041]			
Scaffold24:434987-437007	VEDA_04894														fuNOG02110	EG	Protein involved in ER to Golgi v	IPR004853; Domain of unknown function DUF						
Scaffold24:437401-439071	VEDA_04895														fuNOG02078	L	DNA replication origin binding pr	IPR020796; Origin recognition complex, subun	K02607	DNA replicati	[BR:ko03032][PATH:ko04110			
Scaffold24:439894-441480	VEDA_04896	Y												Zn2Cys6	fuNOG09598	K	Transcription factor with zinc fing	IPR001138; Zn(2)-C6 fungal-type DNA-binding	K00681	Arachidonic a	[PATH:ko00590][PATH:ko004			
Scaffold24:442429-444445	VEDA_04897														fuNOG02193	C	Produces ATP from ADP in the p	IPR000194; ATPase, F1/V1/A1 complex, alpha	K02133	Oxidative phc	[PATH:ko00190][PATH:ko050			
Scaffold24:446179-447063	VEDA_04898														fuNOG06541	C	Cytochrome c oxidase	IPR004203; Cytochrome c oxidase subunit IV	K02263	Oxidative phc	[PATH:ko00190][PATH:ko042			
Scaffold24:447497-449852	VEDA_04899	Y					CBM48, GH13								fuNOG01545	G	1,4-Alpha-Glucan branching enz	IPR004193; Glycoside hydrolase, family 13, N	K00700	Starch and su	[PATH:ko00500]			
Scaffold24:451346-453229	VEDA_04900														fuNOG02938	O	Atp-Dependent Clp protease	IPR003593; AAA+ ATPase domain//IPR00448	K03544	Chaperones a	[BR:ko03110][PATH:ko04112			
Scaffold24:453324-460198	VEDA_04901														fuNOG04749	S	Protein involved in replication for	IPR019021; Methyl methanesulphonate-sensiti						
Scaffold24:460844-464088	VEDA_04902														fuNOG00309	G	2-Oxoglutarate dehydrogenase	IPR001017; Dehydrogenase, E1 component//I	K00164	Citrate cycle	[PATH:ko00020][PATH:ko003			
Scaffold24:465415-467456	VEDA_04903														fuNOG04353	K	RNA polymerase II transcription	IPR005607; BSD//IPR011993; Pleckstrin homo	K03141	Basal transcr	[PATH:ko03022][BR:ko03021			
Scaffold24:467808-472670	VEDA_04904														fuNOG05373	O	Mitochondrial AAA ATPase	IPR001680; WD40 repeat//IPR003593; AAA+	K01509	Purine metab	[PATH:ko00230]			
Scaffold24:477710-480679	VEDA_04905														fuNOG01145	S		IPR005330; MHYT						
Scaffold24:482962-484038	VEDA_04906														fuNOG07146	S	Protein involved in G-protein cou	IPR000342; Regulator of G protein signalling//I						
Scaffold24:488963-492394	VEDA_04907														fuNOG04470	O	Cysteine-Type peptidase	IPR003653; Peptidase C48, SUMO/Sentrin/Ub						
Scaffold24:493348-495460	VEDA_04908														fuNOG04317	CH	Monooxygenase	IPR002938; Monooxygenase, FAD-binding//IP	K00492	Histidine met	[PATH:ko00340][PATH:ko009			
Scaffold24:497282-499145	VEDA_04909														fuNOG06871	S								
Scaffold24:499535-500305	VEDA_04910																							
Scaffold24:500900-502393	VEDA_04911														fuNOG00803	J	S-Adenosyl-L-Methionine-Depen	IPR015915; Kelch-type beta propeller//IPR021						
Scaffold24:504169-504511	VEDA_04912														fuNOG01860	U	Protein involved in autophagy							
Scaffold24:504646-505765	VEDA_04913														fuNOG01860	U	Protein involved in autophagy	IPR001680; WD40 repeat//IPR015943; WD40						
Scaffold24:506537-508655	VEDA_04914														fuNOG04404	V	Protein tyrosine/serine/threonine	IPR000340; Dual specificity phosphatase, cata	K01104	Signal transd				
Scaffold24:509754-512884	VEDA_04915														fuNOG04867	IU	Phospholipase	IPR004177; DDHD	K03018	Purine metab	[PATH:ko00230][PATH:ko002			
Scaffold24:513588-516444	VEDA_04916														fuNOG01609	L	Protein involved in nucleotide-ex	IPR001945; Xeroderma pigmentosum group D	K10844	Basal transcr	[PATH:ko03022][BR:ko03021			
Scaffold24:519522-520537	VEDA_04917	Y												C2H2 zinc finger	fuNOG06200	R	Zinc finger	IPR007087; Zinc finger, C2H2//IPR013087; Zi	K09225	Transcription	[BR:ko03000]			
Scaffold24:522002-527199	VEDA_04918														fuNOG05075	K	Dna-Directed RNA polymerase	IPR000722; RNA polymerase, alpha subunit//I	K02999	Purine metab	[PATH:ko00230][PATH:ko002			
Scaffold24:527859-529235	VEDA_04919														fuNOG03699	A	Protein involved in mRNA metab	IPR010541; Domain of unknown function DUF	K12843	Spliceosome	[PATH:ko03040][BR:ko03041			
Scaffold24:530199-530723	VEDA_04920														fuNOG00777	B	Core component of nucleosome.	IPR000164; Histone H3//IPR007125; Histone (	K11253	Chromosome	[BR:ko03036][PATH:ko05322			
Scaffold24:532040-532462	VEDA_04921														fuNOG07305	B	Core component of nucleosome.	IPR001951; Histone H4//IPR007125; Histone (	K11254	Chromosome	[BR:ko03036][PATH:ko05322			
Scaffold24:536435-537662	VEDA_04922														fuNOG01907	S	Transcription activator protein							
Scaffold24:539579-544378	VEDA_04923														fuNOG00354	E	Pentafunctional AROM polypepti	IPR000623; Shikimate kinase//IPR001381; De	K13830	Phenylalanine	[PATH:ko00400]			
**G-LSR2 (23 genes)**																								
Scaffold4:245621-246493	VEDA_05181																					-5.75	1.23	
Scaffold4:261421-264079	VEDA_05182														fuNOG13091	S		IPR021842; Protein of unknown function DUF3						
Scaffold4:265251-268279	VEDA_05183														fuNOG10889	Z	Protein involved in apoptosis	IPR001440; Tetratricopeptide TPR-1//IPR0021	K10407	Cytoskeleton	[BR:ko04812]	-2.20	-1.55	-1.39
Scaffold4:268562-269884	VEDA_05184														fuNOG11289	S	Protein involved in nucleoside me	IPR000845; Nucleoside phosphorylase domain	K00777	Others		-1.85	-1.30	-2.42
Scaffold4:270400-273676	VEDA_05185														fuNOG10889	Z	Protein involved in apoptosis	IPR001440; Tetratricopeptide TPR-1//IPR0021	K10407	Cytoskeleton	[BR:ko04812]	-2.58		
Scaffold4:280589-284968	VEDA_05186														fuNOG12125	R		IPR001680; WD40 repeat//IPR007111; NACHT	K14855	Ribosome Bic	[BR:ko03009]	-2.48		
Scaffold4:289823-292057	VEDA_05187	Y											CAMK					IPR000719; Protein kinase, catalytic domain//I						1.30
Scaffold4:294043-294735	VEDA_05188														fuNOG03155	S						1.24	1.45	1.63
Scaffold4:299108-299412	VEDA_05189																					-1.07	-1.22	
Scaffold4:301918-303222	VEDA_05190														fuNOG04437	O		IPR003593; AAA+ ATPase domain//IPR00395	K08900	Others		-2.83		
Scaffold4:307357-307932	VEDA_05191																							
Scaffold4:313225-314835	VEDA_05192														fuNOG05935	R		IPR007111; NACHT nucleoside triphosphatase	K00777	Others		-2.35	-2.12	-6.34
Scaffold4:317053-318168	VEDA_05193	Y	Y															IPR011051; Cupin, RmlC-type//IPR013096; Cu				2.60		
Scaffold4:323081-324022	VEDA_05194																	IPR008030; NmrA-like//IPR016040; NAD(P)-bi				3.31	1.09	
Scaffold4:327568-328705	VEDA_05195	Y	Y															IPR000172; Glucose-methanol-choline oxidore	K00108	Glycine, serir	[PATH:ko00260]	7.91		
Scaffold4:332126-333935	VEDA_05196	Y										PHI:544, Botrytis cinerea,						IPR011701; Major facilitator superfamily//IPR0	K01952	Purine metab	[PATH:ko00230]	2.52		
Scaffold4:335027-336067	VEDA_05197														fuNOG05388	Q	Short-Chain dehydrogenase/redu	IPR002198; Short-chain dehydrogenase/reduc				5.57	1.61	
Scaffold4:336752-337753	VEDA_05198														fuNOG06137	Q		IPR002085; Alcohol dehydrogenase superfami	K00344	Energy metal		7.06	3.11	
Scaffold4:338456-339726	VEDA_05199														fuNOG04377	R	Protein involved in N-acylphosph		K13985	Lipid metabol		5.60	1.27	
Scaffold4:342673-343408	VEDA_05200	Y	Y				GH11			Y		PHI:546, Botrytis cinerea,			fuNOG03461	G	Endo-1,4-Beta-Xylanase	IPR001137; Glycoside hydrolase, family 11//IP	K01181	Carbohydrate		-6.60	-1.52	-3.02
Scaffold4:344956-345875	VEDA_05201																	IPR002198; Short-chain dehydrogenase/reduc	K00100	Fructose and	[PATH:ko00051][PATH:ko006	2.31	3.00	3.22
Scaffold4:349504-352947	VEDA_05202																	IPR010285; DNA helicase PIF1, ATP-depende	K15255	DNA replicati	[BR:ko03032]	-4.02	-2.04	-1.97
Scaffold4:353626-355314	VEDA_05203	Y												Homeodomain-like				IPR009057; Homeodomain-like//IPR017877; M				-2.31	-1.02	
**G-LSR3 (101 genes)**																								
Scaffold10:525584-526363	VEDA_06170													MADS-box				IPR002100; Transcription factor, MADS-box						
Scaffold10:526996-528240	VEDA_06171																	IPR001357; BRCT domain						
Scaffold10:530283-530972	VEDA_06172																	IPR001357; BRCT domain						
Scaffold10:533722-535347	VEDA_06173																	IPR020683; Ankyrin repeat-containing domain						
Scaffold10:536880-537215	VEDA_06174																							
Scaffold10:537946-538175	VEDA_06175																							
Scaffold10:538702-539346	VEDA_06176																							
Scaffold10:539539-539989	VEDA_06177	Y	Y																					
Scaffold10:540586-541308	VEDA_06178																							
Scaffold10:554922-556060	VEDA_06179																							
Scaffold10:565505-566035	VEDA_06180																							
Scaffold10:574541-575846	VEDA_06181	Y											CMGC					IPR000719; Protein kinase, catalytic domain//I	K08282	Signal transd		-1.88		
Scaffold10:576403-576885	VEDA_06182																							
Scaffold10:582738-583076	VEDA_06183																							
Scaffold10:585926-586114	VEDA_06184																					1.74		
Scaffold10:589297-589516	VEDA_06185																					1.14	2.02	3.87
Scaffold10:589850-590158	VEDA_06186														fuNOG14415	S		IPR003812; Filamentation induced by cAMP/d	K07341	Cell motility a				
Scaffold10:593309-593686	VEDA_06187														fuNOG10077	S								
Scaffold10:595427-597336	VEDA_06188														fuNOG13091	S		IPR021842; Protein of unknown function DUF3				-5.21		
Scaffold10:598634-599930	VEDA_06189																							
Scaffold10:602639-603089	VEDA_06190	Y	Y																					
Scaffold10:603282-603890	VEDA_06191																							
Scaffold10:604417-604646	VEDA_06192																							
Scaffold10:605377-605712	VEDA_06193																							
Scaffold10:607245-608237	VEDA_06194																	IPR020683; Ankyrin repeat-containing domain						
Scaffold10:611584-612273	VEDA_06195																	IPR001357; BRCT domain						
Scaffold10:614316-615728	VEDA_06196																	IPR001357; BRCT domain						
Scaffold10:616361-617140	VEDA_06197	Y												MADS-box				IPR002100; Transcription factor, MADS-box						
Scaffold10:618067-620055	VEDA_06198														fuNOG04523	C	Ferric reductase	IPR013130; Flavoprotein transmembrane comp						
Scaffold10:620612-621873	VEDA_06199														fuNOG05451	KLO	Poly(Adp)-Ribose polymerase	IPR001357; BRCT domain	K10798	DNA replicati	[BR:ko03032][PATH:ko03410			
Scaffold10:625040-626017	VEDA_06200																	IPR001810; F-box domain, cyclin-like						
Scaffold10:628279-628560	VEDA_06201	Y												MADS-box				IPR002100; Transcription factor, MADS-box						
Scaffold10:636952-637860	VEDA_06202														fuNOG13481	S	Complete genome protein		K14946	Spliceosome	[BR:ko03041]			1.07
Scaffold10:654562-656257	VEDA_06203														fuNOG05451	KLO	Poly(Adp)-Ribose polymerase	IPR001357; BRCT domain					4.36	
Scaffold10:656986-657510	VEDA_06204																							
Scaffold10:668703-669517	VEDA_06205																							
Scaffold10:672641-673732	VEDA_06206																						-1.03	
Scaffold10:674376-674782	VEDA_06207																							
Scaffold10:675089-675745	VEDA_06208																							
Scaffold10:675892-676371	VEDA_06209	Y	Y																					
Scaffold10:676929-677636	VEDA_06210																						-1.68	-1.86
Scaffold10:678391-683442	VEDA_06211																	IPR002156; Ribonuclease H domain//IPR0123						
Scaffold10:685667-686257	VEDA_06212																					-1.05		
Scaffold10:688535-688760	VEDA_06213														fuNOG04235	I		IPR002641; Patatin/Phospholipase A2-related/						
Scaffold10:692255-693515	VEDA_06214																	IPR008397; Alginate lyase domain//IPR00892						
Scaffold10:693851-695341	VEDA_06215	Y	Y															IPR006626; Parallel beta-helix repeat//IPR011						
Scaffold10:696905-697531	VEDA_06216														fuNOG15493	S								
Scaffold10:697780-699057	VEDA_06217														fuNOG07258	S								
Scaffold10:700621-702111	VEDA_06218	Y	Y															IPR006626; Parallel beta-helix repeat//IPR011						
Scaffold10:702447-703707	VEDA_06219																	IPR008397; Alginate lyase domain//IPR00892						
Scaffold10:707202-707427	VEDA_06220														fuNOG04235	I		IPR002641; Patatin/Phospholipase A2-related/						
Scaffold10:710097-710291	VEDA_06221																							
Scaffold10:712515-715736	VEDA_06222														fuNOG13481	S	Complete genome protein	IPR000477; Reverse transcriptase//IPR00215						
Scaffold10:716634-717566	VEDA_06223																							
Scaffold10:718385-720694	VEDA_06224														fuNOG07495	S	Protein involved in lipopolysacch	IPR000719; Protein kinase, catalytic domain//I						
Scaffold10:723495-725102	VEDA_06225																							
Scaffold10:725159-726277	VEDA_06226																							

Scaffold:position	Gene-ID	Pathogen	Secretome	LysM	NLP	SCRPs	CAZymes	Pectin degradation	Cellulose degradation	Hemicellulose degradation	Ligin degradation	PHI_define	Kinase	Transcription factor	eggNOG_ID	eggNOG_class	eggNOG_define	IPR_define	KO_ID	KEGG_Third	BR_family/PATH_way	Responsed to cotton	Responsed to tomato	Responsed to lettuce
Scaffold10:729172-730008	VEDA_06227	Y											AGC					IPR000719; Protein kinase, catalytic domain//I				-1.70		
Scaffold10:731283-732626	VEDA_06228																		K12584	RNA degrada	[PATH:ko03018]		-1.13	-1.15
Scaffold10:734747-735718	VEDA_06229														fuNOG09918	R		IPR000210; BTB/POZ-like//IPR011333; BTB/F				-2.96	-2.24	-1.93
Scaffold10:737399-738085	VEDA_06230																							
Scaffold10:758043-767382	VEDA_06231																					-1.55		
Scaffold10:775416-777143	VEDA_06232	Y										PHI:820, Venturia inaequa			fuNOG04589	Z	Tubulin is the major constituent c	IPR000217; Tubulin//IPR002453; Beta tubulin/	K07375	Phagosome /	[PATH:ko04145][BR:ko04812	-1.52		
Scaffold10:778208-778718	VEDA_06233	Y												Zn2Cys6	fuNOG11881	K		IPR001138; Zn(2)-C6 fungal-type DNA-binding	K09241	Transcription	[BR:ko03000]	-2.38	-2.72	-2.01
Scaffold10:786078-786911	VEDA_06234	Y	Y			Y									fuNOG08344	S						-1.67		
Scaffold10:787215-788193	VEDA_06235	Y											TK					IPR000719; Protein kinase, catalytic domain//I	K00924	Signal transd		-1.21		-1.51
Scaffold10:789205-790461	VEDA_06236														fuNOG11553	S		IPR022198; Protein of unknown function DUF3						
Scaffold10:792314-792937	VEDA_06237	Y											STE					IPR000719; Protein kinase, catalytic domain//I	K08293	Protein kinas	[BR:ko01001]			
Scaffold10:793371-796014	VEDA_06238	Y												C2H2 zinc finger				IPR007087; Zinc finger, C2H2						
Scaffold10:796195-796893	VEDA_06239																							
Scaffold10:798356-798961	VEDA_06240																					1.18		
Scaffold10:799784-800184	VEDA_06241																					1.43		
Scaffold10:804974-805261	VEDA_06242																							
Scaffold10:811498-811908	VEDA_06243																					-8.51		-1.75
Scaffold10:813636-815238	VEDA_06244																					-4.12	-2.81	-2.05
Scaffold10:820874-831003	VEDA_06245																	IPR001214; SET domain						1.42
Scaffold10:831879-832595	VEDA_06246																					-2.10		
Scaffold10:835156-835846	VEDA_06247																							
Scaffold10:839301-840990	VEDA_06248																	IPR013112; FAD-binding 8//IPR013121; Ferric	K05920	Others		-2.04	-1.35	
Scaffold10:842338-843695	VEDA_06249	Y											CMGC		fuNOG06963	T		IPR000719; Protein kinase, catalytic domain//I	K08282	Signal transd		-1.45		
Scaffold10:848311-849478	VEDA_06250	Y												bZIP	fuNOG05088	K	Transcription factor	IPR002112; Transcription factor Jun//IPR0048	K09046	Transcription	[BR:ko03000]			
Scaffold10:849968-850450	VEDA_06251																							
Scaffold10:850478-851628	VEDA_06252																					2.01		1.50
Scaffold10:856322-857159	VEDA_06253														fuNOG13246	S	Phospholipase, patatin family prc	IPR016035; Acyl transferase/acyl hydrolase/ly				3.51		
Scaffold10:858294-859376	VEDA_06254																					-3.85	-1.04	-2.89
Scaffold10:860133-860315	VEDA_06255																					-7.96		
Scaffold10:865336-877840	VEDA_06256	Y											CK1		fuNOG02172	T	Casein kinase I	IPR011009; Protein kinase-like domain	K02218	Protein kinas	[BR:ko01001][PATH:ko04340	-2.48		
Scaffold10:883525-883740	VEDA_06257																							
Scaffold10:886400-887089	VEDA_06258																							
Scaffold10:888914-889099	VEDA_06259																							
Scaffold10:889844-891538	VEDA_06260																	IPR013103; Reverse transcriptase, RNA-depe	K01514	Purine metab	[PATH:ko00230]	-2.79		
Scaffold10:892490-892699	VEDA_06261																							
Scaffold10:899604-900056	VEDA_06262																							
Scaffold10:902951-905574	VEDA_06263																	IPR013103; Reverse transcriptase, RNA-depe	K01514	Purine metab	[PATH:ko00230]	-2.13		
Scaffold10:907399-908088	VEDA_06264																							
Scaffold10:911546-913837	VEDA_06265	Y											TKL					IPR000719; Protein kinase, catalytic domain//I				-1.06		
Scaffold10:931904-932905	VEDA_06266																					-1.92	1.21	1.32
Scaffold10:936873-940700	VEDA_06267														fuNOG13701	R		IPR000477; Reverse transcriptase//IPR00215				-1.60	-1.21	-1.15
Scaffold10:947084-948247	VEDA_06268	Y												Zn2Cys6	fuNOG11570	K		IPR001138; Zn(2)-C6 fungal-type DNA-binding				-5.03	2.29	4.13
Scaffold10:950211-954591	VEDA_06269	Y										PHI:202, Botrytis cinerea,			fuNOG12522	Q	ABC transporter protein	IPR003439; ABC transporter-like//IPR003593;	K08712	Transporters	[BR:ko02000][PATH:ko02010	-3.97		2.66
Scaffold10:958255-960942	VEDA_06270														fuNOG13091	S		IPR021842; Protein of unknown function DUF3				-2.33		
**G-LSR4 (60 genes)**																								
Scaffold17:2211-2992	VEDA_06654																							
Scaffold17:4713-5185	VEDA_06655																							
Scaffold17:8325-9605	VEDA_06656																							
Scaffold17:13126-13656	VEDA_06657																							
Scaffold17:20316-20866	VEDA_06658																		K15053	Endocytosis	[PATH:ko04144]			
Scaffold17:26038-26950	VEDA_06659																					-5.54		
Scaffold17:38395-40743	VEDA_06660														fuNOG07495	S	Protein involved in lipopolysacch	IPR000719; Protein kinase, catalytic domain//I				1.26		1.22
Scaffold17:41495-42295	VEDA_06661																							
Scaffold17:44986-45708	VEDA_06662																							
Scaffold17:46306-46756	VEDA_06663	Y	Y																					
Scaffold17:46936-47616	VEDA_06664																							
Scaffold17:56546-58243	VEDA_06665																							
Scaffold17:62124-62339	VEDA_06666																							
Scaffold17:79707-80076	VEDA_06667																					1.58		
Scaffold17:84473-85170	VEDA_06668																							
Scaffold17:85225-86323	VEDA_06669																							
Scaffold17:87941-89294	VEDA_06670																					-2.02		-1.35
Scaffold17:90170-99522	VEDA_06671																							
Scaffold17:102437-108737	VEDA_06672	Y											CK1		fuNOG02172	T	Casein kinase I	IPR000719; Protein kinase, catalytic domain//I	K02218	Protein kinas	[BR:ko01001][PATH:ko04340	-3.31	-2.40	-2.29
Scaffold17:116212-117299	VEDA_06673																							
Scaffold17:118280-119603	VEDA_06674																					-1.03		
Scaffold17:120673-121101	VEDA_06675																	IPR002110; Ankyrin repeat//IPR020683; Ankyı	K12460	Neurotrophin	[PATH:ko04722]			
Scaffold17:123000-127284	VEDA_06676	Y												Zinc finger, CCHC-type	fuNOG13481	S	Complete genome protein	IPR000477; Reverse transcriptase//IPR00187					1.28	
Scaffold17:129930-130932	VEDA_06677														fuNOG13724	S							3.26	4.43
Scaffold17:131646-132475	VEDA_06678																					-1.67	-1.29	
Scaffold17:136931-139403	VEDA_06679	Y											CAMK					IPR000719; Protein kinase, catalytic domain//I						-1.02
Scaffold17:140950-141618	VEDA_06680																					-1.78		
Scaffold17:143144-143761	VEDA_06681														fuNOG13673	S						-2.04	-1.87	-2.51
Scaffold17:144443-144971	VEDA_06682																					-1.73	-2.04	-1.41
Scaffold17:146861-149378	VEDA_06683																					-2.79	-1.99	-1.78
Scaffold17:150765-151855	VEDA_06684																					-6.33		
Scaffold17:153108-154181	VEDA_06685	Y											STE					IPR000719; Protein kinase, catalytic domain//I				-3.19		
Scaffold17:154874-155251	VEDA_06686														fuNOG10889	Z	Protein involved in apoptosis					-2.60		
Scaffold17:161667-162032	VEDA_06687																	IPR002110; Ankyrin repeat//IPR020683; Ankyı	K00777	Others				6.63
Scaffold17:185586-186020	VEDA_06688																					1.18	1.56	2.75
Scaffold17:189850-190908	VEDA_06689																	IPR008030; NmrA-like//IPR016040; NAD(P)-bi	K00224	Propanoate n	[PATH:ko00640][PATH:ko003	3.91		
Scaffold17:191542-191964	VEDA_06690														fuNOG08741	S							-3.80	-1.68
Scaffold17:192494-193913	VEDA_06691																	IPR013027; FAD-dependent pyridine nucleotid	K00540	Others				
Scaffold17:194657-196069	VEDA_06692														fuNOG00299	G	Monocarboxylic acid transmembr	IPR011701; Major facilitator superfamily//IPR0						
Scaffold17:196734-197912	VEDA_06693														fuNOG05274	K			K11230	Protein kinas	[BR:ko01001][PATH:ko04011	-1.22		
Scaffold17:199901-205782	VEDA_06694																	IPR013103; Reverse transcriptase, RNA-depe	K01514	Purine metab	[PATH:ko00230]	-1.49		
Scaffold17:206326-207006	VEDA_06695																							
Scaffold17:207186-207636	VEDA_06696	Y	Y																					
Scaffold17:208234-208956	VEDA_06697																							
Scaffold17:210868-211332	VEDA_06698																							
Scaffold17:223826-228689	VEDA_06699																					-2.58		
Scaffold17:230815-232020	VEDA_06700																	IPR001357; BRCT domain						
Scaffold17:232650-233429	VEDA_06701	Y												MADS-box				IPR002100; Transcription factor, MADS-box						
Scaffold17:234356-236344	VEDA_06702														fuNOG04523	C	Ferric reductase	IPR013130; Flavoprotein transmembrane comp						
Scaffold17:236901-238526	VEDA_06703														fuNOG05451	KLO	Poly(Adp)-Ribose polymerase	IPR001357; BRCT domain	K10798	DNA replicati	[BR:ko03032][PATH:ko03410		4.19	
Scaffold17:239136-239624	VEDA_06704																	IPR001810; F-box domain, cyclin-like						
Scaffold17:243171-243614	VEDA_06705																	IPR001357; BRCT domain						
Scaffold17:244372-244587	VEDA_06706																							
Scaffold17:245316-255299	VEDA_06707																							
Scaffold17:257298-258112	VEDA_06708																					-3.58	-6.95	-6.95
Scaffold17:262540-262926	VEDA_06709																							
Scaffold17:265124-267377	VEDA_06710														fuNOG14265	S						-2.86	-1.02	
Scaffold17:272562-272852	VEDA_06711																							
Scaffold17:277377-277913	VEDA_06712	Y												Zinc finger, CCHC-type				IPR001878; Zinc finger, CCHC-type				-2.78		
Scaffold17:278086-281718	VEDA_06713														fuNOG13481	S	Complete genome protein	IPR000477; Reverse transcriptase//IPR00513				-2.48		
**JR2 LSRs**																								
**S-LSR1 (70 genes)**																								
contig44576:3178648-3180405	evm.model.contig44576.973																	IPR001023; Heat shock protein Hsp70//IPR01	K03283	Spliceosom	[PATH:ko03040][BR:ko03			
contig44576:3183836-3184060	evm.model.contig44576.974														fuNOG13946	S								
contig44576:3187043-3188146	evm.model.contig44576.975														fuNOG13057	S	C6 transcription factor					-1.47	-1.09	
contig44576:3196834-3198252	evm.model.contig44576.976														fuNOG13583	S							-1.33	-2.08
contig44576:3198428-3199129	evm.model.contig44576.977																							
contig44576:3201786-3203342	evm.model.contig44576.978																	IPR000719; Protein kinase, catalytic domain//I				-1.84	-1.72	-1.88
contig44576:3207509-3209916	evm.model.contig44576.979														fuNOG11553	S		IPR022198; Protein of unknown function DUF3				-1.86	-3.46	
contig44576:3209993-3210823	evm.model.contig44576.980	Y	Y			Y									fuNOG08344	S								
contig44576:3212145-3215154	evm.model.contig44576.981														fuNOG04158	R	Protein involved in nucleoside me	IPR000845; Nucleoside phosphorylase domain	K00777	Others		-2.62	-2.76	-1.91

Scaffold:position	Gene-ID	Pathogen	Secretome	LysM	NLP	SCRPs	CAZymes	Pectin degradation	Cellulose degradation	Hemicellulose degradation	Ligin degradation	PHI_define	Kinase	Transcription factor	eggNOG_ID	eggNOG_class	eggNOG_define	IPR_define	KO_ID	KEGG_Third	BR_family/PATH_way	Responsed to cotton	Responsed to tomato	Responsed to lettuce
contig44576:3221630-3222240	evm.model.contig44576.982														fuNOG06665	S		IPR012312; Haemerythrin/HHE cation-binding				-1.36	-1.74	-2.16
contig44576:3223097-3223723	evm.model.contig44576.983														fuNOG00443	G	Transaldolase is important for the		K00616	Pentose ph	[PATH:ko00030]	-1.21	-1.26	
contig44576:3225402-3227546	evm.model.contig44576.984														fuNOG12237	S						-2.16		
contig44576:3238004-3238690	evm.model.contig44576.985																	IPR007232; Rad52/22 double-strand break rep	K10873	Homologou	[PATH:ko03440][BR:ko03	1.11	1.02	
contig44576:3252602-3253773	evm.model.contig44576.986	Y	Y			Y									fuNOG05714	GO		IPR018535; Domain of unknown function DUF	K01238	Others				
contig44576:3256464-3258092	evm.model.contig44576.987																					-4.31	-5.11	-6.33
contig44576:3259692-3260864	evm.model.contig44576.988	Y	Y			Y									fuNOG05714	GO		IPR018535; Domain of unknown function DUF	K01238	Others			-3.03	-5.34
contig44576:3261570-3262894	evm.model.contig44576.989																	IPR002156; Ribonuclease H domain//IPR0123						
contig44576:3265724-3266718	evm.model.contig44576.990																	IPR003653; Peptidase C48, SUMO/Sentrin/Ub						
contig44576:3270146-3271683	evm.model.contig44576.991	Y												Zn2Cys6	fuNOG05274	K		IPR001138; Zn(2)-C6 fungal-type DNA-binding	K11230	Protein kina	[BR:ko01001][PATH:ko04		-1.70	
contig44576:3272677-3273735	evm.model.contig44576.992																	IPR008030; NmrA-like//IPR016040; NAD(P)-bi	K00224	Propanoate	[PATH:ko00640][PATH:kc	2.25		
contig44576:3275320-3275989	evm.model.contig44576.993																		K00540	Others		-3.13	-9.52	-4.66
contig44576:3277482-3279184	evm.model.contig44576.994														fuNOG00299	G	Monocarboxylic acid transmembr	IPR011701; Major facilitator superfamily//IPR0				-3.50	-3.56	-2.31
contig44576:3279559-3282213	evm.model.contig44576.995	Y												Zn2Cys6				IPR001138; Zn(2)-C6 fungal-type DNA-binding	K11230	Protein kina	[BR:ko01001][PATH:ko04		-1.02	
contig44576:3289449-3290281	evm.model.contig44576.996														fuNOG07601	S	Regulator of secondary metaboli					-1.62	-1.05	
contig44576:3290339-3291010	evm.model.contig44576.997														fuNOG07601	S	Regulator of secondary metaboli		K12584	RNA degra	[PATH:ko03018]	-1.31		
contig44576:3291558-3293947	evm.model.contig44576.998																	IPR000719; Protein kinase, catalytic domain//I						1.44
contig44576:3294937-3296001	evm.model.contig44576.999																	IPR002575; Aminoglycoside phosphotransfera				-1.01	-1.15	
contig44576:3298554-3299258	evm.model.contig44576.1000																							1.26
contig44576:3299579-3300358	evm.model.contig44576.1001																							
contig44576:3302945-3303853	evm.model.contig44576.1002														fuNOG00911	G	Beta glucosidase	IPR001764; Glycoside hydrolase, family 3, N-t	K01188	Starch and	[PATH:ko00500][PATH:kc			
contig44576:3306501-3306695	evm.model.contig44576.1003																							
contig44576:3307348-3308498	evm.model.contig44576.1004																					-2.21	-1.15	-1.75
contig44576:3311436-3313145	evm.model.contig44576.1005	Y										chosporium secalis, Resist			fuNOG04589	Z	Tubulin is the major constituent c	IPR000217; Tubulin//IPR002453; Beta tubulin/	K07375	Phagosome	[PATH:ko04145][BR:ko04	-1.07		
contig44576:3314177-3315442	evm.model.contig44576.1006																							
contig44576:3316211-3319168	evm.model.contig44576.1007														fuNOG04363	R	Protein involved in DNA integrati	IPR001584; Integrase, catalytic core//IPR0123	K01514	Purine meta	[PATH:ko00230]			
contig44576:3321586-3323338	evm.model.contig44576.1008														fuNOG03696	S						-1.79	-2.05	
contig44576:3324655-3325109	evm.model.contig44576.1009														fuNOG08398	TU	Protein involved in mRNA stabiliz	IPR006760; Endosulphine				-1.22	1.24	
contig44576:3325247-3325804	evm.model.contig44576.1010																							
contig44576:3327613-3328461	evm.model.contig44576.1011														fuNOG08344	S						-1.07	-1.96	-2.49
contig44576:3330651-3334386	evm.model.contig44576.1012	Y											STE					IPR000719; Protein kinase, catalytic domain//I	K08819	Protein kina	[BR:ko01001]	1.40	-1.48	
contig44576:3334871-3335593	evm.model.contig44576.1013																							
contig44576:3337643-3338341	evm.model.contig44576.1014																							
contig44576:3338473-3339255	evm.model.contig44576.1015																					-2.23	-2.76	-1.78
contig44576:3340102-3340707	evm.model.contig44576.1016																						-1.97	
contig44576:3341530-3341930	evm.model.contig44576.1017																							1.23
contig44576:3346275-3346939	evm.model.contig44576.1018																							1.28
contig44576:3476721-3476921	evm.model.contig44576.1019														fuNOG02805	S	Transmembrane receptor							
contig44576:3479183-3479572	evm.model.contig44576.1020																							
contig44576:3480092-3480663	evm.model.contig44576.1021																							
contig44576:3490381-3491181	evm.model.contig44576.1022																	IPR002641; Patatin/Phospholipase A2-related/					-1.97	
contig44576:3496285-3497020	evm.model.contig44576.1023														fuNOG03867	R		IPR013761; Sterile alpha motif/pointed domain						
contig44576:3499664-3500251	evm.model.contig44576.1024	Y												Zn2Cys6				IPR001138; Zn(2)-C6 fungal-type DNA-binding				-1.20		-1.08
contig44576:3503683-3504051	evm.model.contig44576.1025																							1.54
contig44576:3505539-3505769	evm.model.contig44576.1026																							
contig44576:3506509-3506739	evm.model.contig44576.1027																							
contig44576:3507627-3508115	evm.model.contig44576.1028														fuNOG06415	S						-1.51		
contig44576:3510920-3511120	evm.model.contig44576.1029														fuNOG13481	S	Complete genome protein							
contig44576:3511599-3513437	evm.model.contig44576.1030														fuNOG00593	S	BZIP transcription factor							
contig44576:3516390-3516689	evm.model.contig44576.1031																						-2.13	
contig44576:3519654-3519863	evm.model.contig44576.1032														fuNOG00593	S	BZIP transcription factor						-1.48	
contig44576:3521212-3521622	evm.model.contig44576.1033																							
contig44576:3525250-3525645	evm.model.contig44576.1034																							
contig44576:3527864-3531751	evm.model.contig44576.1035																	IPR001584; Integrase, catalytic core//IPR0123						
contig44576:3532055-3532729	evm.model.contig44576.1036																					1.70		
contig44576:3533623-3534792	evm.model.contig44576.1037	Y	Y			Y									fuNOG05714	GO		IPR018535; Domain of unknown function DUF	K01238	Others		-3.52	-3.39	-4.77
contig44576:3540766-3541536	evm.model.contig44576.1038																						-6.17	-6.17
contig44576:3542977-3543512	evm.model.contig44576.1039	Y												C2H2 zinc finger				IPR007087; Zinc finger, C2H2//IPR015880; Zi				-2.05	-4.78	-8.18
contig44576:3550818-3553026	evm.model.contig44576.1040														fuNOG13057	S	C6 transcription factor					-1.40		
contig44576:3553306-3553533	evm.model.contig44576.1041																					-1.74		
contig44576:3660332-3661954	evm.model.contig44576.1042														fuNOG00346	A	Pre-Mrna splicing factor	IPR000061; SWAP/Surp//IPR022030; Pre-mR	K12825	Spliceosom	[PATH:ko03040][BR:ko03	1.80	1.24	1.92
**S-LSR2 (46 genes)**																								
contig1569:936436-937538	evm.model.contig1569.316	Y												C2H2 zinc finger				IPR007087; Zinc finger, C2H2				-4.12	-4.92	-2.28
contig1569:1027733-1028056	evm.model.contig1569.317																					-2.83	-2.96	-2.39
contig1569:1036061-1036471	evm.model.contig1569.318																					-1.87		
contig1569:1036943-1037116	evm.model.contig1569.319																					-1.36		
contig1569:1037270-1037452	evm.model.contig1569.320																					-2.40	-2.74	-2.53
contig1569:1042909-1044683	evm.model.contig1569.321	Y											CAMK					IPR000253; Forkhead-associated (FHA) doma						1.42
contig1569:1047491-1049151	evm.model.contig1569.322														fuNOG12639	L		IPR006055; Exonuclease//IPR012337; Ribonu						
contig1569:1053315-1053719	evm.model.contig1569.323																							
contig1569:1060414-1061134	evm.model.contig1569.324																						1.24	1.15
contig1569:1063263-1063415	evm.model.contig1569.325																							
contig1569:1063900-1065798	evm.model.contig1569.326	Y												Homeodomain-like				IPR009057; Homeodomain-like						
contig1569:1067000-1067905	evm.model.contig1569.327																	IPR002575; Aminoglycoside phosphotransfera				-2.77	-2.72	-1.13
contig1569:1068529-1072212	evm.model.contig1569.328														fuNOG11965	R		IPR001680; WD40 repeat//IPR007111; NACH	K00777	Others		-2.58	-1.80	-1.72
contig1569:1072769-1074575	evm.model.contig1569.329														fuNOG01304	G	Iron transporter protein	IPR011701; Major facilitator superfamily//IPR0				1.44	1.98	2.57
contig1569:1074664-1075083	evm.model.contig1569.330																	IPR001680; WD40 repeat//IPR015943; WD40/				-3.19	-2.32	-2.89
contig1569:1075507-1078387	evm.model.contig1569.331																	IPR002110; Ankyrin repeat//IPR020683; Ankyı	K07126	General fur		-1.73	-1.19	
contig1569:1080763-1081095	evm.model.contig1569.332																					-1.94		
contig1569:1082300-1083247	evm.model.contig1569.333														fuNOG00529	R	Zinc finger	IPR013087; Zinc finger, C2H2-type/integrase,						
contig1569:1083667-1084179	evm.model.contig1569.334																					-2.32		-1.29
contig1569:1085202-1087332	evm.model.contig1569.335														fuNOG13091	S		IPR021842; Protein of unknown function DUF3					-1.39	
contig1569:1089003-1090441	evm.model.contig1569.336																	IPR021842; Protein of unknown function DUF3						
contig1569:1104769-1105344	evm.model.contig1569.337																							
contig1569:1111007-1111866	evm.model.contig1569.338																						-3.14	
contig1569:1118839-1119864	evm.model.contig1569.339																							
contig1569:1121763-1122417	evm.model.contig1569.340														fuNOG11028	M								
contig1569:1123281-1123771	evm.model.contig1569.341																	IPR002110; Ankyrin repeat//IPR020683; Ankyı	K10380	Cytoskeleto	[BR:ko04812]			
contig1569:1127413-1127760	evm.model.contig1569.342	Y	Y																					
contig1569:1144884-1145515	evm.model.contig1569.343																							
contig1569:1156509-1156887	evm.model.contig1569.344	Y	Y																			-3.93	-3.73	-3.96
contig1569:1158471-1158905	evm.model.contig1569.345																							
contig1569:1159639-1161294	evm.model.contig1569.346	Y												Zinc finger, CCHC-type				IPR001878; Zinc finger, CCHC-type//IPR0048	K06125	Ubiquinone	[PATH:ko00130][BR:ko01			
contig1569:1165565-1166327	XLOC_00170.mRNA	Y	Y			Y																-1.56	-1.92	-1.59
contig1569:1167830-1169209	evm.model.contig1569.347																					-3.17	-5.78	-3.43
contig1569:1272675-1273097	evm.model.contig1569.348																		K04605	G protein-c	[BR:ko04030][PATH:ko04			
contig1569:1290546-1291232	evm.model.contig1569.349																							
contig1569:1292913-1293884	evm.model.contig1569.350														fuNOG09918	R		IPR000210; BTB/POZ-like//IPR011333; BTB/F					-1.18	-1.80
contig1569:1296005-1297769	evm.model.contig1569.351																		K12584	RNA degra	[PATH:ko03018]	-3.39	-3.03	-3.43
contig1569:1299044-1299880	evm.model.contig1569.352	Y											AGC					IPR000719; Protein kinase, catalytic domain//I						
contig1569:1300781-1301569	evm.model.contig1569.353																					-1.06	-3.21	
contig1569:1302380-1303893	evm.model.contig1569.354																						-2.68	-1.40
contig1569:1304168-1305536	evm.model.contig1569.355																							
contig1569:1308358-1310667	evm.model.contig1569.356														fuNOG07495	S	Protein involved in lipopolysacch	IPR000719; Protein kinase, catalytic domain//I						
contig1569:1310936-1312591	evm.model.contig1569.357	Y												Zinc finger, CCHC-type				IPR001878; Zinc finger, CCHC-type//IPR0048	K06125	Ubiquinone	[PATH:ko00130][BR:ko01			
contig1569:1326205-1327113	evm.model.contig1569.358														fuNOG13481	S	Complete genome protein		K14946	Spliceosom	[BR:ko03041]		-1.14	
contig1569:1441557-1441987	evm.model.contig1569.359	Y												MADS-box				IPR002100; Transcription factor, MADS-box						
contig1569:1449547-1450337	evm.model.contig1569.360																					-1.43	-3.47	-4.59
**S-LSR3 (57 genes)**																								
contig45503:522437-522976	evm.model.contig45503.186																							2.03
contig45503:524215-525792	evm.model.contig45503.187	Y												Zn2Cys6				IPR007219; Transcription factor, fungi				1.33		2.51
contig45503:530847-533446	evm.model.contig45503.188														fuNOG03071	P	Potassium ion transmembrane tr	IPR003445; Cation transporter//IPR004773; P				-2.75	-2.58	-2.69
contig45503:534519-536064	evm.model.contig45503.189														fuNOG04633	P	Solute:Hydrogen antiporter prote	IPR006153; Cation/H+ exchanger	K03316	Other ion-c				

Scaffold:position	Gene-ID	Pathogen	Secretome	LysM	NLP	SCRPs	CAZymes	Pectin degradation	Cellulose degradation	Hemicellulose degradation	Ligin degradation	PHI_define	Kinase	Transcription factor	eggNOG_ID	eggNOG_class	eggNOG_define	IPR_define	KO_ID	KEGG_Third	BR_family/PATH_way	Responsed to cotton	Responsed to tomato	Responsed to lettuce
contig45503:639492-640644	evm.model.contig45503.190																					-1.08	-1.21	
contig45503:654464-655793	evm.model.contig45503.191														fuNOG06483	R			K12584	RNA degra	[PATH:ko03018]	-1.92	-1.32	
contig45503:658271-659689	evm.model.contig45503.192																							
contig45503:662091-662600	evm.model.contig45503.193														fuNOG01084	B	Protein involved in protein deace	IPR003000; NAD-dependent deacetylase, sirtu	K01463	Metabolism				
contig45503:663198-663623	evm.model.contig45503.194																							
contig45503:664293-664766	evm.model.contig45503.195																							
contig45503:667907-669307	evm.model.contig45503.196														fuNOG06483	R			K12584	RNA degra	[PATH:ko03018]	-4.14	-2.46	-4.62
contig45503:670812-671375	evm.model.contig45503.197	Y	Y															IPR007112; Expansin/pollen allergen, DPBB d						-1.79
contig45503:682610-682885	evm.model.contig45503.198														fuNOG00899	P	Alpha-Glucoside:Hydrogen symp		K08141	Transporter	[BR:ko02000]			
contig45503:688525-689154	evm.model.contig45503.199	Y												Zn2Cys6				IPR001138; Zn(2)-C6 fungal-type DNA-binding				-3.34	-3.60	-9.25
contig45503:690149-690385	evm.model.contig45503.200																							
contig45503:690559-692145	evm.model.contig45503.201														fuNOG05451	KLO	Poly(Adp)-Ribose polymerase	IPR001357; BRCT domain	K10798	DNA replica	[BR:ko03032][PATH:ko03	-2.24	-2.49	-1.21
contig45503:692660-694662	evm.model.contig45503.202														fuNOG04523	C	Ferric reductase	IPR013130; Flavoprotein transmembrane com				-1.38	-2.01	-1.67
contig45503:771769-774101	evm.model.contig45503.203																	IPR021842; Protein of unknown function DUF					-5.61	
contig45503:791893-792165	evm.model.contig45503.204																							
contig45503:799310-801705	evm.model.contig45503.205																					1.47		1.93
contig45503:804567-805064	evm.model.contig45503.206																					1.41	1.92	1.86
contig45503:807510-810755	evm.model.contig45503.207																					2.09	2.02	2.46
contig45503:812474-813767	evm.model.contig45503.208	Y												Zn2Cys6	fuNOG10087	K		IPR001138; Zn(2)-C6 fungal-type DNA-binding	K00681	Arachidonic	[PATH:ko00590][PATH:kc			1.04
contig45503:814635-816162	evm.model.contig45503.209																						-1.36	
contig45503:816479-817424	evm.model.contig45503.210																	IPR007568; RTA-like protein				5.02	4.04	3.30
contig45503:818236-821074	evm.model.contig45503.211	Y					GT1								fuNOG03623	G	Protein involved in lipid glycosyla	IPR002213; UDP-glucuronosyl/UDP-glucosyltr	K05841	Glycosyltra	[BR:ko01003]			
contig45503:821867-824379	evm.model.contig45503.212	Y	Y				GH78	Y							fuNOG05571	R		IPR008902; Bacterial alpha-L-rhamnosidase//I	K05989	Others			2.24	1.77
contig45503:824805-825484	evm.model.contig45503.213																							
contig45503:826243-827064	evm.model.contig45503.214																					-3.64	-2.70	-3.68
contig45503:829607-830379	evm.model.contig45503.215																							
contig45503:833418-834914	evm.model.contig45503.216														fuNOG12723	H	Tetrahydrofolylpolyglutamate syr	IPR001645; Folylpolyglutamate synthetase//IP	K01930	Folate bios	[PATH:ko00790]			
contig45503:834943-836852	evm.model.contig45503.217																	IPR011990; Tetratricopeptide-like helical				-2.55	-2.20	-1.67
contig45503:840484-841732	evm.model.contig45503.218	Y												C2H2 zinc finger	fuNOG03341	K	Protein involved in response to c	IPR007087; Zinc finger, C2H2//IPR013087; Zi	K09234	Transcriptic	[BR:ko03000]			
contig45503:843733-844484	evm.model.contig45503.219																							
contig45503:846957-847676	evm.model.contig45503.220																							
contig45503:848020-849789	evm.model.contig45503.221																							
contig45503:850531-850929	evm.model.contig45503.222																					1.18		1.83
contig45503:852422-855960	evm.model.contig45503.223																	IPR002110; Ankyrin repeat//IPR020683; Ankyr	K00777	Others				
contig45503:859160-859321	evm.model.contig45503.224																							
contig45503:861923-865412	evm.model.contig45503.225																	IPR007111; NACHT nucleoside triphosphatase						1.87
contig45503:868961-870002	evm.model.contig45503.226																							1.10
contig45503:870848-872118	evm.model.contig45503.227																							
contig45503:872305-872535	evm.model.contig45503.228																							1.29
contig45503:872916-873215	evm.model.contig45503.229																					1.70	1.61	2.38
contig45503:875095-876368	evm.model.contig45503.230																	IPR021842; Protein of unknown function DUF				1.17	1.56	1.57
contig45503:916541-918064	evm.model.contig45503.231														fuNOG05614	I	3-Ketoacyl-Coa protein	IPR002155; Thiolase//IPR016038; Thiolase-lik	K00632	Fatty acid r	[PATH:ko00071][PATH:kc			
contig45503:918628-921079	evm.model.contig45503.232	Y												Zn2Cys6	fuNOG04931	K		IPR001138; Zn(2)-C6 fungal-type DNA-binding				-2.88	-2.56	-2.41
contig45503:921499-924540	evm.model.contig45503.233														fuNOG02319	Q	Protein involved in fatty acid met	IPR002198; Short-chain dehydrogenase/reduc	K14729	Lipid metab		-1.50	-1.44	
contig45503:925609-926916	evm.model.contig45503.234														fuNOG04046	C	Monooxygenase	IPR002938; Monooxygenase, FAD-binding//IP	K00480	Dioxin degr	[PATH:ko00621][PATH:kc	-3.50	-1.53	-1.22
contig45503:927492-929034	evm.model.contig45503.235														fuNOG04232	C	Aldehyde dehydrogenase	IPR015590; Aldehyde dehydrogenase domain/	K00155	Limonene a	[PATH:ko00903][PATH:kc	-6.11	-3.21	-4.73
contig45503:929764-931481	evm.model.contig45503.236	Y					AA7				Y				fuNOG12271	R	FAD binding domain protein	IPR006094; FAD linked oxidase, N-terminal//IF	K00309	Others			-4.61	-3.85
contig45503:933073-934902	evm.model.contig45503.237																	IPR001202; WW/Rsp5/WWP//IPR010730; He	K00688	Starch and	[PATH:ko00500][PATH:kc	-1.84	-1.87	-2.13
contig45503:997456-998187	evm.model.contig45503.238																	IPR001357; BRCT domain				-2.60	-2.88	-4.70
contig45503:1002130-1002537	evm.model.contig45503.239																	IPR001357; BRCT domain						-6.89
contig45503:1008018-1009070	evm.model.contig45503.240																	IPR001810; F-box domain, cyclin-like				-3.57	-2.96	-4.19
contig45503:1011302-1011732	evm.model.contig45503.241	Y												MADS-box				IPR002100; Transcription factor, MADS-box						
contig45503:1017875-1019520	evm.model.contig45503.242														fuNOG05263	R		IPR006680; Amidohydrolase 1//IPR011059; M	K01564	Chloroalkar	[PATH:ko00625][PATH:kc	-1.95	-3.03	-1.85
**S-LSR4 (43 genes)**																								
contig45503:3321055-3321276	evm.model.contig45503.948																							
contig45503:3322352-3330466	evm.model.contig45503.949	Y										ne grisea, Effector (plant av			fuNOG04527	I	Polyketide synthase	IPR000794; Beta-ketoacyl synthase//IPR0012	K00344	Energy met		-2.23		-2.26
contig45503:3342607-3343833	evm.model.contig45503.950																					-1.90	-2.60	-1.74
contig45503:3345170-3346147	evm.model.contig45503.951	Y											CAMK		fuNOG12577	T		IPR000719; Protein kinase, catalytic domain//I	K08884	Protein kina	[BR:ko01001]			
contig45503:3346150-3346445	evm.model.contig45503.952																					-1.63	-1.41	-1.46
contig45503:3347781-3348185	evm.model.contig45503.953																					-1.01		
contig45503:3351152-3351827	evm.model.contig45503.954																						1.00	
contig45503:3352444-3353717	evm.model.contig45503.955																					-1.63		
contig45503:3357649-3358068	evm.model.contig45503.956																					2.30		3.73
contig45503:3359923-3360561	evm.model.contig45503.957	Y											CK1		fuNOG02172	T	Casein kinase I	IPR011009; Protein kinase-like domain//IPR01	K02218	Protein kina	[BR:ko01001][PATH:ko04			
contig45503:3363273-3364109	evm.model.contig45503.958														fuNOG10550	S			K12584	RNA degra	[PATH:ko03018]	1.25		2.31
contig45503:3365040-3366695	evm.model.contig45503.959	Y												Zinc finger, CCHC-type				IPR001878; Zinc finger, CCHC-type//IPR0048	K06125	Ubiquinone	[PATH:ko00130][BR:ko01			
contig45503:3367429-3367758	evm.model.contig45503.960																	IPR009799; EthD domain//IPR011008; Dimeric						
contig45503:3369497-3370606	evm.model.contig45503.961	Y					CE10								fuNOG05578	V		IPR002168; Lipase, GDXG, active site//IPR01	K01066	Tropane, pi	[PATH:ko00960][PATH:kc	3.49	1.57	3.42
contig45503:3371319-3371948	evm.model.contig45503.962														fuNOG05935	R		IPR002110; Ankyrin repeat//IPR020683; Ankyr	K06867	General fur		-1.47		
contig45503:3487777-3490696	evm.model.contig45503.963																	IPR001584; Integrase, catalytic core//IPR0123	K12619	Ribosome b	[PATH:ko03008][BR:ko03		-1.23	
contig45503:3492391-3493092	evm.model.contig45503.964																						-1.43	
contig45503:3493107-3495275	evm.model.contig45503.965														fuNOG13481	S	Complete genome protein	IPR000477; Reverse transcriptase//IPR00513					-1.34	
contig45503:3495925-3496869	evm.model.contig45503.966																							
contig45503:3496933-3497130	evm.model.contig45503.967																							
contig45503:3498529-3500115	evm.model.contig45503.968														fuNOG03679	G		IPR005829; Sugar transporter, conserved site	K00492	Histidine m	[PATH:ko00340][PATH:kc			
contig45503:3500632-3503536	evm.model.contig45503.969														fuNOG15292	L	Telomerase inhibitor	IPR010285; DNA helicase PIF1, ATP-depende				-1.41	-1.76	-1.64
contig45503:3503846-3504927	evm.model.contig45503.970																	IPR008030; NmrA-like//IPR016040; NAD(P)-bi	K00831	Methane m	[PATH:ko00680][PATH:kc			1.09
contig45503:3506624-3507280	evm.model.contig45503.971														fuNOG08233	Q		IPR002198; Short-chain dehydrogenase/reduc	K00540	Others		-1.14	-1.57	
contig45503:3507944-3510594	evm.model.contig45503.972	Y												Homeobox	fuNOG04242	K	Zinc finger	IPR001356; Homeodomain//IPR007087; Zinc f					-1.42	
contig45503:3510905-3511105	evm.model.contig45503.973																					1.32		1.21
contig45503:3512265-3512529	evm.model.contig45503.974																							
contig45503:3513386-3514643	evm.model.contig45503.975														fuNOG02450	G	Alpha-Glucosidase	IPR000322; Glycoside hydrolase, family 31	K01187	Galactose r	[PATH:ko00052][PATH:kc		-1.43	
contig45503:3516338-3518194	evm.model.contig45503.976														fuNOG01728	E	Amino acid protein	IPR002293; Amino acid/polyamine transporter	K03293	Other ion-c			1.57	3.52
contig45503:3520749-3522023	evm.model.contig45503.977														fuNOG08750	E	1-Aminocyclopropane-1-Carboxy	IPR001176; 1-aminocyclopropane-1-carboxyla	K14270	Amino acid	[BR:ko01007]	-1.29		
contig45503:3524237-3524670	evm.model.contig45503.978																					-1.12		
contig45503:3525235-3527018	evm.model.contig45503.979																							
contig45503:3531243-3532891	evm.model.contig45503.980																							-3.35
contig45503:3533470-3534486	evm.model.contig45503.981																	IPR002156; Ribonuclease H domain//IPR0123						
contig45503:3534587-3535401	evm.model.contig45503.982																	IPR000477; Reverse transcriptase						
contig45503:3539408-3539821	evm.model.contig45503.983																					-1.16		
contig45503:3540908-3543937	evm.model.contig45503.984														fuNOG13481	S	Complete genome protein	IPR000477; Reverse transcriptase//IPR00215					-1.47	
contig45503:3543950-3544405	evm.model.contig45503.985																						-1.94	
contig45503:3545020-3547701	evm.model.contig45503.986	Y					GT32											IPR007577; Glycosyltransferase, DXD sugar-b					-1.38	
contig45503:3550031-3551368	evm.model.contig45503.987																	IPR022698; Protein of unknown function DUF					-1.99	
contig45503:3644203-3645319	evm.model.contig45503.988	Y					GH109								fuNOG04660	R	Protein involved in NADPH reger	IPR000683; Oxidoreductase, N-terminal//IPR0	K00118	Others				
contig45503:3647615-3650111	evm.model.contig45503.989	Y												Zn2Cys6	fuNOG05332	K		IPR001138; Zn(2)-C6 fungal-type DNA-binding						
contig45503:3650613-3652612	evm.model.contig45503.990														fuNOG00351	P	Transporter protein	IPR001046; Natural resistance-associated ma	K12346	Other ion-c			-3.13	
**VdLs.17 LSRs**																								
**L-LSR1 (89 genes)**																								
Supercontig_1.4:43-761	VDAG_02345																	IPR021842; Protein of unknown function DUF						
Supercontig_1.4:6893-8118	VDAG_02346																	IPR003817; Phosphatidylserine decarboxylase	K01613	Glyceropho	[PATH:ko00564]			
Supercontig_1.4:8620-10422	VDAG_02347	Y												Zn2Cys6	fuNOG09546	K	Bzip transcription factor	IPR001138; Zn(2)-C6 fungal-type DNA-binding					-1.16	
Supercontig_1.4:11332-12090	VDAG_02348	Y												bZIP	fuNOG05088	K	Transcription factor	IPR002112; Transcription factor Jun//IPR0048	K09046	Transcriptic	[BR:ko03000]			
Supercontig_1.4:12584-12844	VDAG_02349																					1.25	1.79	2.13
Supercontig_1.4:19224-20787	VDAG_02350														fuNOG02140	T	PAS domain-containing protein	IPR000014; PAS//IPR013655; PAS fold-3	K10838	Nucleotide	[PATH:ko03420][BR:ko03			
Supercontig_1.4:27364-28193	VDAG_02351																							
Supercontig_1.4:28892-30214	VDAG_02352																					1.17	1.89	1.19
Supercontig_1.4:30383-31705	VDAG_02353																							
Supercontig_1.4:42898-44265	VDAG_02354	Y										PHI:153, Magnaporthe gri	CMGC		fuNOG01722	T	Mitogen-Activated protein kinase	IPR000719; Protein kinase, catalytic domain//I	K04441	Protein kina	[BR:ko01001][PATH:ko04	1.52	1.03	1.15
Supercontig_1.4:54817-56316	VDAG_02355																							
Supercontig_1.4:58973-60235	VDAG_02356	Y					GH18					PHI:144, Trichoderma vire			fuNOG00900	G	Chitinase	IPR001223; Glycoside hydrolase, family 18, ca	K01183	Amino suga	[PATH:ko00520]			
Supercontig_1.4:63899-65932	VDAG_02357														fuNOG04516	D	Protein involved in regulation of (	IPR013763; Cyclin-like//IPR013922; Cyclin PH	K06656	Cell cycle -	[PATH:ko04111]			
Supercontig_1.4:66286-67359	VDAG_02358	Y					GH128								fuNOG07224	G		IPR013781; Glycoside hydrolase, subgroup, ca						

Scaffold:position	Gene-ID	Pathogen	Secretome	LysM	NLP	SCRPs	CAZymes	Pectin degradation	Cellulose degradation	Hemicellulose degradation	Ligin degradation	PHI_define	Kinase	Transcription factor	eggNOG_ID	eggNOG_class	eggNOG_define	IPR_define	KO_ID	KEGG_Third	BR_family/PATH_way	Responsed to cotton	Responsed to tomato	Responsed to lettuce
Supercontig_1.4:67586-68626	VDAG_02359																							
Supercontig_1.4:68939-70763	VDAG_02360														fuNOG04391	C	Ferric-Chelate reductase	IPR013112; FAD-binding 8//IPR013121; Ferric				3.28	4.56	4.36
Supercontig_1.4:72150-72773	VDAG_02361																							
Supercontig_1.4:73188-73964	VDAG_02362																							
Supercontig_1.4:79139-80455	VDAG_02363																							
Supercontig_1.4:82524-84786	VDAG_02364																							
Supercontig_1.4:86718-88347	VDAG_02365														fuNOG10844	S		IPR002575; Aminoglycoside phosphotransfera						
Supercontig_1.4:91897-92550	VDAG_02366																					4.14	4.51	4.46
Supercontig_1.4:93104-94428	VDAG_02367																					1.90	2.69	2.58
Supercontig_1.4:95932-97984	VDAG_02368																							
Supercontig_1.4:99502-99900	VDAG_02369																							
Supercontig_1.4:100494-102315	VDAG_02370	Y											CK1		fuNOG02172	T	Casein kinase I	IPR000719; Protein kinase, catalytic domain//I	K02218	Protein kina	[BR:ko01001][PATH:ko04			
Supercontig_1.4:103013-103645	VDAG_02371																							
Supercontig_1.4:104251-105920	VDAG_02372														fuNOG10550	S								
Supercontig_1.4:106419-107091	VDAG_02373																						1.29	1.09
Supercontig_1.4:108324-108999	VDAG_02374																					1.71	1.50	2.07
Supercontig_1.4:109974-115586	VDAG_02375																	IPR010285; DNA helicase PIF1, ATP-depende				-2.82	-3.36	-3.10
Supercontig_1.4:120078-122989	VDAG_02376	Y					GT1								fuNOG03623	G	Protein involved in lipid glycosyla	IPR002213; UDP-glucuronosyl/UDP-glucosyltr	K05841	Glycosyltra	[BR:ko01003]			1.27
Supercontig_1.4:123487-124852	VDAG_02377	Y												Zn2Cys6	fuNOG10087	K		IPR001138; Zn(2)-C6 fungal-type DNA-binding				2.30	2.12	2.32
Supercontig_1.4:125988-126947	VDAG_02378																	IPR007568; RTA-like protein						
Supercontig_1.4:128100-133050	VDAG_02379																	IPR010285; DNA helicase PIF1, ATP-depende				-2.14	-2.47	-2.33
Supercontig_1.4:133287-134607	VDAG_02380																					-2.17	-1.70	-1.29
Supercontig_1.4:142521-143602	VDAG_02381																	IPR016040; NAD(P)-binding domain				3.36	3.43	-1.01
Supercontig_1.4:144371-145294	VDAG_02382																	IPR008030; NmrA-like//IPR016040; NAD(P)-bi				3.68	3.97	
Supercontig_1.4:145758-147831	VDAG_02383	Y												Zn2Cys6	fuNOG01717	K		IPR001138; Zn(2)-C6 fungal-type DNA-binding						
Supercontig_1.4:150139-151160	VDAG_02384														fuNOG02692	R		IPR008030; NmrA-like//IPR016040; NAD(P)-bi				6.57	6.80	
Supercontig_1.4:151420-151837	VDAG_02385																							
Supercontig_1.4:154067-154372	VDAG_02386														fuNOG10077	S								
Supercontig_1.4:159509-160925	VDAG_02387	Y										PHI:232, Cryptococcus ne		C2H2 zinc finger	fuNOG03341	K	Protein involved in response to c	IPR007087; Zinc finger, C2H2//IPR013087; Zi	K09201	Transcriptic	[BR:ko03000][BR:ko0303			
Supercontig_1.4:164601-165853	VDAG_02388																						-1.12	-1.33
Supercontig_1.4:168279-169073	VDAG_02389																	IPR008331; Ferritin/DPS protein domain//IPR0	K00522	Porphyrin a	[PATH:ko00860][PATH:kc			
Supercontig_1.4:171933-172278	VDAG_02390														fuNOG14491	S		IPR021331; Protein of unknown function DUF2					1.27	1.27
Supercontig_1.4:173141-174127	VDAG_02391														fuNOG13638	L	Reverse transcriptase							
Supercontig_1.4:175269-175979	VDAG_02392																							
Supercontig_1.4:176832-178242	VDAG_02393														fuNOG10890	K	Protein involved in hyperosmotic	IPR022210; Transcription activator, osmotic-st				-1.68		
Supercontig_1.4:183825-185366	VDAG_02394														fuNOG13117	R		IPR002110; Ankyrin repeat//IPR020683; Ankyı	K06867	General fun				
Supercontig_1.4:188512-190095	VDAG_02395														fuNOG13859	S		IPR018289; MULE transposase domain				-1.92	-1.94	-1.94
Supercontig_1.4:190492-191872	VDAG_02396	Y	Y				GH18					PHI:144, Trichoderma vire			fuNOG00900	G	Chitinase	IPR001223; Glycoside hydrolase, family 18, ca	K01183	Amino suga	[PATH:ko00520]			
Supercontig_1.4:193040-195412	VDAG_02397														fuNOG13246	S	Phospholipase, patatin family prc					-6.06	-4.94	-4.92
Supercontig_1.4:197332-198478	VDAG_02398	Y	Y			Y									fuNOG05714	GO		IPR018535; Domain of unknown function DUF	K01238	Others				
Supercontig_1.4:203505-206122	VDAG_02399																					-1.23	-1.35	
Supercontig_1.4:206758-207617	VDAG_02400																					-3.58		-2.03
Supercontig_1.4:207951-210612	VDAG_02401														fuNOG01097	S		IPR011043; Galactose oxidase/kelch, beta-prc						
Supercontig_1.4:214154-215990	VDAG_02402														fuNOG01948	P	Protein involved in cellular zinc ic	IPR002524; Cation efflux protein	K14688	Mineral abs	[PATH:ko04978]	1.26		
Supercontig_1.4:217530-217984	VDAG_02403																					-3.67	-2.49	-3.60
Supercontig_1.4:221124-222753	VDAG_02404																	IPR011701; Major facilitator superfamily//IPR0	K08157	Transporter	[BR:ko02000]			
Supercontig_1.4:223177-224870	VDAG_02405																							
Supercontig_1.4:225271-226734	VDAG_02406																		K00777	Others				
Supercontig_1.4:228022-228515	VDAG_02407	Y												Zn2Cys6	fuNOG11881	K		IPR001138; Zn(2)-C6 fungal-type DNA-binding	K09241	Transcriptic	[BR:ko03000]	-1.31		
Supercontig_1.4:229424-230104	VDAG_02408	Y												bZIP	fuNOG05088	K	Transcription factor	IPR004827; Basic-leucine zipper domain//IPR	K04450	Transcriptic	[BR:ko03000][PATH:ko04	-1.70	-1.67	-1.41
Supercontig_1.4:236454-236861	VDAG_02409														fuNOG08182	O	Protein tag	IPR000626; Ubiquitin//IPR019955; Ubiquitin su	K12160	RNA transp	[PATH:ko03013][BR:ko04	2.45	2.77	2.94
Supercontig_1.4:238142-238814	VDAG_02410																					-3.81	-2.46	-2.52
Supercontig_1.4:244573-245400	VDAG_02411	Y												bZIP	fuNOG05088	K	Transcription factor	IPR002112; Transcription factor Jun//IPR0048					1.02	1.07
Supercontig_1.4:247984-248269	VDAG_02412														fuNOG09725	S								
Supercontig_1.4:249250-250218	VDAG_02413																	IPR003830; (2R)-phospho-3-sulpholactate syn	K06911	General fun				
Supercontig_1.4:252491-254314	VDAG_02414														fuNOG13859	S		IPR018289; MULE transposase domain				-1.33	-1.73	-2.07
Supercontig_1.4:258971-260336	VDAG_02415	Y												bZIP	fuNOG05088	K	Transcription factor	IPR004827; Basic-leucine zipper domain//IPR	K09046	Transcriptic	[BR:ko03000]	-1.39	-1.12	-1.01
Supercontig_1.4:261572-263215	VDAG_02416														fuNOG11260	G	Protein involved in alginic acid ca	IPR008397; Alginate lyase domain//IPR00892						
Supercontig_1.4:265252-266129	VDAG_02417														fuNOG06830	S	Calcium ion binding protein	IPR007736; Caleosin						1.09
Supercontig_1.4:266564-267420	VDAG_02418	Y	Y																					1.91
Supercontig_1.4:275086-276477	VDAG_02419	Y											CMGC					IPR000719; Protein kinase, catalytic domain//I				-1.24	-1.01	
Supercontig_1.4:277185-277505	VDAG_02420														fuNOG03902	K	Transcription factor							
Supercontig_1.4:278192-281272	VDAG_02421														fuNOG05704	U	Gtpase						1.77	2.08
Supercontig_1.4:281615-282025	VDAG_02422																						1.47	1.18
Supercontig_1.4:284209-285038	VDAG_02423																					1.42	1.74	1.52
Supercontig_1.4:285362-286147	VDAG_02424																							
Supercontig_1.4:289043-290323	VDAG_02425														fuNOG04437	O		IPR003593; AAA+ ATPase domain//IPR00395	K08900	Others				
Supercontig_1.4:290781-291188	VDAG_02426														fuNOG04437	O			K08900	Others		-2.98	-2.34	-2.64
Supercontig_1.4:291807-293462	VDAG_02427																	IPR010730; Heterokaryon incompatibility				-2.67	-2.59	-2.95
Supercontig_1.4:294652-297234	VDAG_02428														fuNOG13638	L	Reverse transcriptase					-1.48	-1.84	-1.64
Supercontig_1.4:299198-300193	VDAG_02429														fuNOG11724	S	Protein involved in apoptosis	IPR002641; Patatin/Phospholipase A2-related/						
Supercontig_1.4:302628-302792	VDAG_02430																					-1.19		
Supercontig_1.4:303820-304653	VDAG_02431																	IPR011009; Protein kinase-like domain//IPR01				-2.24	-1.83	-1.82
Supercontig_1.4:305375-306421	VDAG_02432														fuNOG07495	S	Protein involved in lipopolysacch					-2.96	-2.79	-2.82
Supercontig_1.4:309821-310677	VDAG_02433																							
**L-LSR2 (97 genes)**																								
Supercontig_1.8:884942-886247	VDAG_04831																	IPR000953; Chromo domain/shadow//IPR0161				-1.05		
Supercontig_1.8:890839-895795	VDAG_04832														fuNOG10494	S		IPR001584; Integrase, catalytic core//IPR0123				-1.12	-1.16	
Supercontig_1.8:895833-896211	VDAG_04833	Y										PHI:144, Trichoderma vire			fuNOG00900	G	Chitinase	IPR001223; Glycoside hydrolase, family 18, ca	K01183	Amino suga	[PATH:ko00520]	-1.09	-1.16	
Supercontig_1.8:896667-897122	VDAG_04834	Y			Y							PHI:740, Botrytis elliptica,						IPR008701; Necrosis inducing				-2.09	-2.23	-1.98
Supercontig_1.8:901876-902282	VDAG_04835																							
Supercontig_1.8:902596-903084	VDAG_04836																		K00831	Methane m	[PATH:ko00680][PATH:kc			
Supercontig_1.8:904019-906639	VDAG_04837	Y												Homeobox	fuNOG04242	K	Zinc finger	IPR001356; Homeodomain//IPR007087; Zinc f	K09359	Transcriptic	[BR:ko03000][PATH:ko04			
Supercontig_1.8:907326-908078	VDAG_04838	Y												Negative transcriptional r	fuNOG06801	R	FMN binding protein	IPR007396; Negative transcriptional regulator/	K07734	Transcriptic	[BR:ko03000]	1.67	1.80	2.03
Supercontig_1.8:909269-910342	VDAG_04839																	IPR003010; Nitrilase/cyanide hydratase	K01502	Styrene dec	[PATH:ko00643]	1.65	1.71	1.42
Supercontig_1.8:910845-912110	VDAG_04840														fuNOG08750	E	1-Aminocyclopropane-1-Carboxy	IPR001176; 1-aminocyclopropane-1-carboxyla	K01762	Cysteine ar	[PATH:ko00270][BR:ko01	1.85	1.84	1.45
Supercontig_1.8:914657-915473	VDAG_04841														fuNOG08233	Q		IPR002198; Short-chain dehydrogenase/reduc	K00100	Fructose ar	[PATH:ko00051][PATH:kc	1.94	2.46	2.38
Supercontig_1.8:915937-916538	VDAG_04842																					1.43	2.64	2.83
Supercontig_1.8:916591-917846	VDAG_04843	Y												Zn2Cys6	fuNOG10087	K		IPR001138; Zn(2)-C6 fungal-type DNA-binding	K00681	Arachidonic	[PATH:ko00590][PATH:kc			
Supercontig_1.8:921166-923481	VDAG_04844														fuNOG03071	P	Potassium ion transmembrane tr	IPR003445; Cation transporter				1.95	1.85	1.92
Supercontig_1.8:924991-925650	VDAG_04845																					2.18	2.64	2.78
Supercontig_1.8:925414-927042	VDAG_04846														fuNOG04633	P	Solute:Hydrogen antiporter prote	IPR006153; Cation/H+ exchanger	K03316	Other ion-c				
Supercontig_1.8:927730-930399	VDAG_04847														fuNOG03071	P	Potassium ion transmembrane tr	IPR003445; Cation transporter//IPR004773; P						
Supercontig_1.8:932206-933421	VDAG_04848														fuNOG13481	S	Complete genome protein					-4.55	-4.09	-4.16
Supercontig_1.8:934156-935984	VDAG_04849																					-1.94	-1.45	-1.41
Supercontig_1.8:937482-938825	VDAG_04850																							
Supercontig_1.8:942886-945202	VDAG_04851														fuNOG13859	S								
Supercontig_1.8:945639-948292	VDAG_04852																							
Supercontig_1.8:949120-951523	VDAG_04853	Y	Y												fuNOG00807	O	Heat shock protein	IPR001023; Heat shock protein Hsp70//IPR01	K03283	Spliceosom	[PATH:ko03040][BR:ko03	-2.00	-1.88	
Supercontig_1.8:956720-957392	VDAG_04854																					-1.01		
Supercontig_1.8:959577-963293	VDAG_04855																	IPR015880; Zinc finger, C2H2-like				-2.48	-2.51	-2.27
Supercontig_1.8:964540-965283	VDAG_04856																					-1.05		
Supercontig_1.8:966281-967433	VDAG_04857																					-2.61	-3.06	-3.12
Supercontig_1.8:968687-969086	VDAG_04858																							
Supercontig_1.8:969384-969922	VDAG_04859																							
Supercontig_1.8:973534-975277	VDAG_04860														fuNOG00149	S		IPR013860; Protein of unknown function DUF1				-1.45	-2.05	-1.43
Supercontig_1.8:980426-980980	VDAG_04861																					-1.35		
Supercontig_1.8:989409-991220	VDAG_04862																	IPR013112; FAD-binding 8//IPR013121; Ferric	K05920	Others				
Supercontig_1.8:991522-992586	VDAG_04863																							
Supercontig_1.8:1001233-1002237	VDAG_04864														fuNOG03138	Q	Protein involved in phosphatidic a	IPR002198; Short-chain dehydrogenase/reduc	K06123	Glycerophо	[PATH:ko00564][PATH:kc			
Supercontig_1.8:1006070-1008903	VDAG_04865														fuNOG02730	O	Serine-Type endopeptidase	IPR000209; Peptidase S8/S53, subtilisin/kexin						
Supercontig_1.8:1013150-1013410	VDAG_04866																							
Supercontig_1.8:1016718-1016834	VDAG_04867																							
Supercontig_1.8:1017502-1017642	VDAG_04868																						1.60	

Scaffold:position	Gene-ID	Pathogen	Secretome	LysM	NLP	SCRPs	CAZymes	Pectin degradation	Cellulose degradation	Hemicellulose degradation	Ligin degradation	PHI_define	Kinase	Transcription factor	eggNOG_ID	eggNOG_class	eggNOG_define	IPR_define	KO_ID	KEGG_Third	BR_family/PATH_way	Responsed to cotton	Responsed to tomato	Responsed to lettuce
Supercontig_1.8:1018651-1018862	VDAG_04869																	IPR016163; Aldehyde dehydrogenase, C-termi						
Supercontig_1.8:1041555-1042415	VDAG_04870																	IPR002575; Aminoglycoside phosphotransfera				-1.11		
Supercontig_1.8:1042667-1043418	VDAG_04871																						1.43	1.05
Supercontig_1.8:1043758-1044282	VDAG_04872																	IPR015880; Zinc finger, C2H2-like						
Supercontig_1.8:1049305-1049866	VDAG_04873																							
Supercontig_1.8:1053810-1054160	VDAG_04874																							
Supercontig_1.8:1054657-1055379	VDAG_04875																							
Supercontig_1.8:1056250-1057424	VDAG_04876	Y	Y			Y									fuNOG05714	GO		IPR018535; Domain of unknown function DUF	K01238	Others				
Supercontig_1.8:1057765-1058399	VDAG_04877																					3.08	2.98	3.56
Supercontig_1.8:1058576-1061788	VDAG_04878														fuNOG13246	S	Phospholipase, patatin family prc						1.53	1.77
Supercontig_1.8:1062318-1064517	VDAG_04879	Y					GH18					PHI:144, Trichoderma vire			fuNOG00900	G	Chitinase	IPR001223; Glycoside hydrolase, family 18, ca	K01183	Amino suga	[PATH:ko00520]			
Supercontig_1.8:1066821-1067579	VDAG_04880														fuNOG02520	P	Protein involved in drug transmei	IPR002528; Multi antimicrobial extrusion protei	K14445					
Supercontig_1.8:1079165-1082115	VDAG_04881	Y					GT32											IPR007577; Glycosyltransferase, DXD sugar-b				1.46	1.96	2.26
Supercontig_1.8:1094274-1095922	VDAG_04882																						-2.51	-1.71
Supercontig_1.8:1098244-1099014	VDAG_04883																							
Supercontig_1.8:1100147-1101930	VDAG_04884																					-1.04		
Supercontig_1.8:1102603-1104502	VDAG_04885																	IPR003010; Nitrilase/cyanide hydratase//IPR0	K01502	Styrene deg	[PATH:ko00643]	-1.28		
Supercontig_1.8:1105142-1106416	VDAG_04886														fuNOG08750	E	1-Aminocyclopropane-1-Carboxy	IPR001176; 1-aminocyclopropane-1-carboxyla	K14270	Amino acid	[BR:ko01007]		1.20	
Supercontig_1.8:1108935-1110791	VDAG_04887														fuNOG01728	E	Amino acid protein	IPR002293; Amino acid/polyamine transporter	K03293	Other ion-c				
Supercontig_1.8:1112486-1113743	VDAG_04888														fuNOG02450	G	Alpha-Glucosidase	IPR000322; Glycoside hydrolase, family 31	K01187	Galactose r	[PATH:ko00052][PATH:kc			-1.30
Supercontig_1.8:1114600-1115500	VDAG_04889																							
Supercontig_1.8:1115591-1116335	VDAG_04890																					1.59	2.06	1.90
Supercontig_1.8:1116535-1119185	VDAG_04891	Y												Homeobox	fuNOG04242	K	Zinc finger	IPR001356; Homeodomain//IPR007087; Zinc f						
Supercontig_1.8:1119849-1120559	VDAG_04892														fuNOG08233	Q		IPR002198; Short-chain dehydrogenase/reduc	K00540	Others				
Supercontig_1.8:1121471-1121984	VDAG_04893																					-1.02		
Supercontig_1.8:1122206-1123287	VDAG_04894																	IPR008030; NmrA-like//IPR016040; NAD(P)-bi	K00831	Methane m	[PATH:ko00680][PATH:kc			
Supercontig_1.8:1123597-1125772	VDAG_04895														fuNOG15292	L	Telomerase inhibitor	IPR010285; DNA helicase PIF1, ATP-depende				-3.73	-3.65	-3.92
Supercontig_1.8:1127435-1128579	VDAG_04896														fuNOG03679	G		IPR005829; Sugar transporter, conserved site	K12604	RNA degra	[PATH:ko03018]	-2.22	-2.21	-2.10
Supercontig_1.8:1142104-1142532	VDAG_04897																							
Supercontig_1.8:1143161-1143825	VDAG_04898																						1.28	
Supercontig_1.8:1148170-1148570	VDAG_04899																						2.25	1.78
Supercontig_1.8:1152395-1154981	VDAG_04900														fuNOG12562	S						2.74	3.06	
Supercontig_1.8:1155416-1156361	VDAG_04901																	IPR000719; Protein kinase, catalytic domain//I					1.30	
Supercontig_1.8:1157657-1159210	VDAG_04902														fuNOG11553	S		IPR022198; Protein of unknown function DUF3						
Supercontig_1.8:1160528-1162935	VDAG_04903														fuNOG11553	S		IPR022198; Protein of unknown function DUF3				-2.94	-3.30	-3.91
Supercontig_1.8:1163012-1163842	VDAG_04904	Y	Y			Y									fuNOG08344	S						-1.23		
Supercontig_1.8:1164857-1168584	VDAG_04905														fuNOG04158	R	Protein involved in nucleoside me	IPR000845; Nucleoside phosphorylase domain	K00777	Others		-1.53	-1.81	-3.27
Supercontig_1.8:1174696-1175306	VDAG_04906														fuNOG06665	S		IPR012312; Haemerythrin/HHE cation-binding						
Supercontig_1.8:1176163-1176789	VDAG_04907														fuNOG00443	G	Transaldolase is important for the		K00616	Pentose ph	[PATH:ko00030]			
Supercontig_1.8:1177017-1178517	VDAG_04908																	IPR023753; Pyridine nucleotide-disulphide oxic	K00359	Others				
Supercontig_1.8:1186107-1186541	VDAG_04909																					1.86	1.91	
Supercontig_1.8:1195121-1195729	VDAG_04910	Y												Zn2Cys6				IPR001138; Zn(2)-C6 fungal-type DNA-binding				1.96	2.27	2.98
Supercontig_1.8:1200228-1200933	VDAG_04911	Y												bZIP	fuNOG06421	K	Protein dimerization	IPR011616; bZIP transcription factor, bZIP-1				1.04	2.17	2.47
Supercontig_1.8:1202518-1204295	VDAG_04912														fuNOG00593	S	BZIP transcription factor						1.45	1.83
Supercontig_1.8:1205515-1205996	VDAG_04913														fuNOG06415	S								
Supercontig_1.8:1208045-1208679	VDAG_04914																							
Supercontig_1.8:1209496-1210004	VDAG_04915																							
Supercontig_1.8:1211016-1211369	VDAG_04916																							
Supercontig_1.8:1218177-1219041	VDAG_04917														fuNOG11375	S		IPR002110; Ankyrin repeat//IPR020683; Ankyı				-1.47		
Supercontig_1.8:1219701-1220834	VDAG_04918														fuNOG05935	R		IPR002110; Ankyrin repeat//IPR020683; Ankyı	K06867	General fur		2.33	3.20	2.64
Supercontig_1.8:1221733-1222655	VDAG_04919																	IPR002168; Lipase, GDXG, active site//IPR01	K05968	Others		3.15	2.94	1.12
Supercontig_1.8:1224394-1224723	VDAG_04920																	IPR009799; EthD domain//IPR011008; Dimeric				1.76	2.57	1.52
Supercontig_1.8:1227330-1229023	VDAG_04921														fuNOG10550	S						1.26	1.37	1.38
Supercontig_1.8:1231734-1232372	VDAG_04922	Y											CK1		fuNOG02172	T	Casein kinase I	IPR011009; Protein kinase-like domain//IPR01	K02218	Protein kina	[BR:ko01001][PATH:ko04			
Supercontig_1.8:1234227-1234646	VDAG_04923																							
Supercontig_1.8:1238578-1239851	VDAG_04924																							
Supercontig_1.8:1240468-1241143	VDAG_04925																					2.36	2.92	3.27
Supercontig_1.8:1243873-1244150	VDAG_04926																							
Supercontig_1.8:1244182-1245343	VDAG_04927																					-2.02	-1.89	-1.93
**L-LSR3 (149 genes)**																								
Supercontig_1.9:587769-588193	VDAG_05143	Y												MADS-box				IPR002100; Transcription factor, MADS-box						
Supercontig_1.9:589148-589937	VDAG_05144																							
Supercontig_1.9:592119-592442	VDAG_05145																							
Supercontig_1.9:594047-595662	VDAG_05146														fuNOG05451	KLO	Poly(Adp)-Ribose polymerase	IPR001357; BRCT domain	K10798	DNA replica	[BR:ko03032][PATH:ko03			
Supercontig_1.9:596219-598186	VDAG_05147														fuNOG04523	C	Ferric reductase	IPR013130; Flavoprotein transmembrane com						
Supercontig_1.9:599134-599913	VDAG_05148	Y												MADS-box				IPR002100; Transcription factor, MADS-box						
Supercontig_1.9:600543-601226	VDAG_05149																	IPR001357; BRCT domain					-1.17	
Supercontig_1.9:607262-608848	VDAG_05150																	IPR002110; Ankyrin repeat//IPR020683; Ankyı						
Supercontig_1.9:610370-610705	VDAG_05151																							
Supercontig_1.9:613949-614889	VDAG_05152																					-1.94	-1.91	-1.84
Supercontig_1.9:622857-624084	VDAG_05153	Y												Zn2Cys6				IPR001138; Zn(2)-C6 fungal-type DNA-binding				-2.36	-2.01	-2.69
Supercontig_1.9:628715-629496	VDAG_05154																							
Supercontig_1.9:631185-632089	VDAG_05155																							
Supercontig_1.9:632641-633663	VDAG_05156	Y												C2H2 zinc finger				IPR007087; Zinc finger, C2H2//IPR013087; Zi						
Supercontig_1.9:648780-650479	VDAG_05157																		K15053	Endocytosis	[PATH:ko04144]			
Supercontig_1.9:652499-653308	VDAG_05158														fuNOG11864	S						-3.81	-3.11	-2.09
Supercontig_1.9:655517-656554	VDAG_05159																	IPR001610; PAC motif						
Supercontig_1.9:673434-675782	VDAG_05160														fuNOG07495	S	Protein involved in lipopolysacch	IPR000719; Protein kinase, catalytic domain//I				3.29	3.29	3.25
Supercontig_1.9:676081-677334	VDAG_05161																					3.02	3.23	2.82
Supercontig_1.9:677553-678047	VDAG_05162																						1.90	
Supercontig_1.9:691930-693154	VDAG_05163																							
Supercontig_1.9:698884-699714	VDAG_05164																					6.11	5.97	4.61
Supercontig_1.9:701958-702550	VDAG_05165																						2.29	2.01
Supercontig_1.9:705992-706392	VDAG_05166																					2.74	3.06	2.59
Supercontig_1.9:707215-707820	VDAG_05167																							
Supercontig_1.9:709283-709981	VDAG_05168																							
Supercontig_1.9:710060-712805	VDAG_05169	Y												C2H2 zinc finger				IPR007087; Zinc finger, C2H2//IPR013762; Int						
Supercontig_1.9:713241-716976	VDAG_05170														fuNOG11553	S		IPR001245; Serine-threonine/tyrosine-protein k				-2.05		
Supercontig_1.9:718042-719020	VDAG_05171	Y											TK					IPR000719; Protein kinase, catalytic domain//I	K00924	Signal trans		2.46	2.76	2.59
Supercontig_1.9:722653-723031	VDAG_05172																						1.01	
Supercontig_1.9:724433-726189	VDAG_05173														fuNOG03696	S						-1.14		
Supercontig_1.9:727524-728034	VDAG_05174	Y												Zn2Cys6	fuNOG11881	K		IPR001138; Zn(2)-C6 fungal-type DNA-binding	K09241	Transcriptic	[BR:ko03000]			
Supercontig_1.9:729099-730826	VDAG_05175	Y										PHI:820, Venturia inaequa			fuNOG04589	Z	Tubulin is the major constituent c	IPR000217; Tubulin//IPR002453; Beta tubulin/	K07375	Phagosome	[PATH:ko04145][BR:ko04			
Supercontig_1.9:732485-733950	VDAG_05176														fuNOG10889	Z	Protein involved in apoptosis							
Supercontig_1.9:735540-736233	VDAG_05177																						1.36	1.19
Supercontig_1.9:737319-739830	VDAG_05178														fuNOG04158	R	Protein involved in nucleoside me	IPR002110; Ankyrin repeat//IPR020683; Ankyı						
Supercontig_1.9:741973-743038	VDAG_05179														fuNOG12971	S						1.31		1.02
Supercontig_1.9:764258-764695	VDAG_05180	Y	Y	Y		Y												IPR002482; Peptidoglycan-binding Lysin subgı	K03791	General fur				
Supercontig_1.9:765565-766910	VDAG_05181	Y										PHI:59, Colletotrichum lag			fuNOG05833	Q	Reductase	IPR002198; Short-chain dehydrogenase/reduc	K00059	Fatty acid b	[PATH:ko00061][PATH:kc	-2.68	-2.27	-2.05
Supercontig_1.9:767757-770284	VDAG_05182														fuNOG01097	S		IPR011043; Galactose oxidase/kelch, beta-prc				-1.63	-1.90	-1.57
Supercontig_1.9:794671-794991	VDAG_05183																					1.95	2.30	2.37
Supercontig_1.9:800373-801094	VDAG_05184																					1.03	1.26	1.27
Supercontig_1.9:805054-806713	VDAG_05185																						-2.65	
Supercontig_1.9:812684-813370	VDAG_05186																							
Supercontig_1.9:813646-814200	VDAG_05187														fuNOG13570	S						-1.05		
Supercontig_1.9:815051-816022	VDAG_05188																	IPR000210; BTB/POZ-like//IPR011333; BTB/F						
Supercontig_1.9:818143-819486	VDAG_05189																		K12584	RNA degra	[PATH:ko03018]	-2.45	-1.97	-2.07
Supercontig_1.9:820761-821597	VDAG_05190	Y											AGC					IPR000719; Protein kinase, catalytic domain//I					1.06	
Supercontig_1.9:822725-823473	VDAG_05191																					-2.22	-2.24	-1.60
Supercontig_1.9:824097-825610	VDAG_05192																					3.24	3.85	3.46
Supercontig_1.9:825885-827253	VDAG_05193																							
Supercontig_1.9:830075-832384	VDAG_05194														fuNOG07495	S	Protein involved in lipopolysacch	IPR000719; Protein kinase, catalytic domain//I						
Supercontig_1.9:836437-837286	VDAG_05195																						2.02	1.26
Supercontig_1.9:851205-852521	VDAG_05196																					-5.18	-5.23	-3.75

Scaffold:position	Gene-ID	Pathogen	Secretome	LysM	NLP	SCRPs	CAZymes	Pectin degradation	Cellulose degradation	Hemicellulose degradation	Ligin degradation	PHI_define	Kinase	Transcription factor	eggNOG_ID	eggNOG_class	eggNOG_define	IPR_define	KO_ID	KEGG_Third	BR_family/PATH_way	Responsed to cotton	Responsed to tomato	Responsed to lettuce
Supercontig_1.9:853115-853804	VDAG_05197																	IPR001357; BRCT domain						
Supercontig_1.9:861495-862121	VDAG_05198																							
Supercontig_1.9:865555-866624	VDAG_05199																	IPR001357; BRCT domain						
Supercontig_1.9:869535-869798	VDAG_05200																							
Supercontig_1.9:871189-872822	VDAG_05201																							
Supercontig_1.9:876798-876920	VDAG_05202																							
Supercontig_1.9:893271-893695	VDAG_05203	Y												MADS-box				IPR002100; Transcription factor, MADS-box						
Supercontig_1.9:894650-895439	VDAG_05204																							
Supercontig_1.9:895649-896786	VDAG_05205																	IPR001810; F-box domain, cyclin-like					-5.13	-5.13
Supercontig_1.9:897552-897947	VDAG_05206																							
Supercontig_1.9:899552-901213	VDAG_05207														fuNOG05451	KLO	Poly(Adp)-Ribose polymerase	IPR001357; BRCT domain	K10798	DNA replica	[BR:ko03032][PATH:ko03	-2.97	-5.19	
Supercontig_1.9:901770-903737	VDAG_05208														fuNOG04523	C	Ferric reductase	IPR013130; Flavoprotein transmembrane com				-6.30	-3.29	-4.59
Supercontig_1.9:904685-905464	VDAG_05209	Y												MADS-box				IPR002100; Transcription factor, MADS-box						
Supercontig_1.9:906095-907234	VDAG_05210																	IPR001357; BRCT domain				-1.87	-2.62	-2.26
Supercontig_1.9:907965-908686	VDAG_05211																	IPR001451; Bacterial transferase hexapeptide						
Supercontig_1.9:917031-917890	VDAG_05212																							
Supercontig_1.9:918477-919499	VDAG_05213	Y												C2H2 zinc finger				IPR007087; Zinc finger, C2H2//IPR013087; Zi						
Supercontig_1.9:925484-926014	VDAG_05214																							
Supercontig_1.9:927687-928645	VDAG_05215																	IPR001357; BRCT domain						
Supercontig_1.9:943947-944351	VDAG_05216																							
Supercontig_1.9:944918-945700	VDAG_05217																						-1.08	-1.13
Supercontig_1.9:947957-948340	VDAG_05218																							
Supercontig_1.9:950998-952414	VDAG_05219														fuNOG04437	O		IPR003593; AAA+ ATPase domain//IPR00395	K08900	Others		1.40		2.32
Supercontig_1.9:953565-953875	VDAG_05220																					-1.59		
Supercontig_1.9:954077-954729	VDAG_05221																						1.16	
Supercontig_1.9:956542-957501	VDAG_05222																							
Supercontig_1.9:960558-961206	VDAG_05223																							
Supercontig_1.9:976649-977520	VDAG_05224														fuNOG10723	R			K12584	RNA degra	[PATH:ko03018]			
Supercontig_1.9:979345-979705	VDAG_05225																					-1.54		
Supercontig_1.9:982778-983753	VDAG_05226	Y												C2H2 zinc finger	fuNOG03341	K	Protein involved in response to c	IPR007087; Zinc finger, C2H2//IPR013087; Zi	K09229	Transcriptic	[BR:ko03000]	-1.71	-1.01	-1.20
Supercontig_1.9:984947-985192	VDAG_05227																							
Supercontig_1.9:987792-988516	VDAG_05228																					-1.24		
Supercontig_1.9:992975-993687	VDAG_05229														fuNOG06421	K	Protein dimerization					1.63	2.27	1.53
Supercontig_1.9:1007634-1008260	VDAG_05230																							
Supercontig_1.9:1009671-1010741	VDAG_05231														fuNOG01413	T	Protein involved in cellular zinc ic	IPR004254; Hly-III-related	K07036	General fur				
Supercontig_1.9:1012001-1012853	VDAG_05232																							
Supercontig_1.9:1015182-1016445	VDAG_05233														fuNOG13718	S						-2.87	-2.59	-2.32
Supercontig_1.9:1016980-1017697	VDAG_05234																					-4.77	-4.01	-3.80
Supercontig_1.9:1024623-1025417	VDAG_05235	Y												MADS-box				IPR002100; Transcription factor, MADS-box						
Supercontig_1.9:1026040-1027260	VDAG_05236																	IPR001357; BRCT domain				-4.62	-4.26	-4.43
Supercontig_1.9:1028100-1029137	VDAG_05237														fuNOG02616	K		IPR001451; Bacterial transferase hexapeptide	K00661	Others				
Supercontig_1.9:1032227-1033606	VDAG_05238																					-4.85	-3.73	-5.30
Supercontig_1.9:1035169-1037709	VDAG_05239																	IPR002110; Ankyrin repeat//IPR020683; Anky						
Supercontig_1.9:1037940-1038281	VDAG_05240																							
Supercontig_1.9:1041691-1042416	VDAG_05241																							
Supercontig_1.9:1043882-1045362	VDAG_05242																	IPR022198; Protein of unknown function DUF3						
Supercontig_1.9:1048396-1050152	VDAG_05243	Y												Zn2Cys6				IPR001138; Zn(2)-C6 fungal-type DNA-binding				1.95	1.86	1.80
Supercontig_1.9:1078989-1079519	VDAG_05244																							
Supercontig_1.9:1084529-1084851	VDAG_05245																							
Supercontig_1.9:1085835-1086191	VDAG_05246																							
Supercontig_1.9:1087670-1088975	VDAG_05247	Y											CMGC					IPR000719; Protein kinase, catalytic domain//I						
Supercontig_1.9:1089532-1090014	VDAG_05248																							
Supercontig_1.9:1093013-1093996	VDAG_05249																							
Supercontig_1.9:1094376-1094732	VDAG_05250																							
Supercontig_1.9:1095500-1096775	VDAG_05251																	IPR001623; Heat shock protein DnaJ, N-termi				-2.97		
Supercontig_1.9:1099198-1099708	VDAG_05252	Y												Zn2Cys6	fuNOG04186	K		IPR001138; Zn(2)-C6 fungal-type DNA-binding				1.26		1.28
Supercontig_1.9:1101977-1102321	VDAG_05253																							
Supercontig_1.9:1102992-1103300	VDAG_05254														fuNOG14415	S		IPR003812; Filamentation induced by cAMP/d	K07341	Cell motility		-7.69	-7.69	
Supercontig_1.9:1104356-1104532	VDAG_05255																							
Supercontig_1.9:1105109-1105920	VDAG_05256	Y												Zn2Cys6	fuNOG11881	K		IPR001138; Zn(2)-C6 fungal-type DNA-binding				-1.05	-1.13	-1.11
Supercontig_1.9:1108545-1110836	VDAG_05257														fuNOG13091	S		IPR021842; Protein of unknown function DUF3						
Supercontig_1.9:1113862-1114932	VDAG_05258														fuNOG01413	T	Protein involved in cellular zinc ic	IPR004254; Hly-III-related	K07036	General fur				
Supercontig_1.9:1118622-1118957	VDAG_05259																							
Supercontig_1.9:1120479-1122065	VDAG_05260																	IPR002110; Ankyrin repeat//IPR020683; Anky						
Supercontig_1.9:1128281-1128658	VDAG_05261																					-3.81	-4.15	-3.99
Supercontig_1.9:1159357-1160882	VDAG_05262																							
Supercontig_1.9:1167913-1169266	VDAG_05263																							
Supercontig_1.9:1173076-1174496	VDAG_05264																	IPR002575; Aminoglycoside phosphotransfera						
Supercontig_1.9:1174624-1175398	VDAG_05265																					-1.33		
Supercontig_1.9:1179312-1181252	VDAG_05266														fuNOG00413	R								
Supercontig_1.9:1182836-1183975	VDAG_05267																					-4.00	-2.59	-1.94
Supercontig_1.9:1184505-1185907	VDAG_05268																							
Supercontig_1.9:1186628-1188709	VDAG_05269	Y											CK1		fuNOG02172	T	Casein kinase I	IPR000719; Protein kinase, catalytic domain//I	K02218	Protein kina	[BR:ko01001][PATH:ko04			
Supercontig_1.9:1200199-1201955	VDAG_05270														fuNOG04158	R	Protein involved in nucleoside me	IPR002110; Ankyrin repeat//IPR020683; Anky	K10380	Cytoskeleto	[BR:ko04812]	-1.43	-2.00	-2.70
Supercontig_1.9:1211116-1211774	VDAG_05271														fuNOG13724	S							2.12	1.83
Supercontig_1.9:1212488-1213317	VDAG_05272																						1.65	1.31
Supercontig_1.9:1217834-1220245	VDAG_05273	Y											CAMK					IPR000719; Protein kinase, catalytic domain//I				-1.39	-1.03	
Supercontig_1.9:1221792-1222460	VDAG_05274																					-1.45	-1.66	-1.08
Supercontig_1.9:1223709-1224603	VDAG_05275														fuNOG13673	S						1.29	1.50	1.27
Supercontig_1.9:1224862-1226595	VDAG_05276																					-1.10		
Supercontig_1.9:1227135-1230220	VDAG_05277																							
Supercontig_1.9:1233012-1233393	VDAG_05278																							
Supercontig_1.9:1233949-1235022	VDAG_05279	Y											STE					IPR000719; Protein kinase, catalytic domain//I				-1.25		
Supercontig_1.9:1235413-1236985	VDAG_05280																							
Supercontig_1.9:1238127-1238606	VDAG_05281																						1.34	1.27
Supercontig_1.9:1240357-1242872	VDAG_05282														fuNOG04158	R	Protein involved in nucleoside me	IPR002110; Ankyrin repeat//IPR020683; Anky				2.16	2.94	2.73
Supercontig_1.9:1245684-1246283	VDAG_05283														fuNOG12971	S						2.00		1.33
Supercontig_1.9:1246692-1247229	VDAG_05284																					-5.18	-4.65	-2.82
Supercontig_1.9:1249202-1250173	VDAG_05285																							
Supercontig_1.9:1252715-1254403	VDAG_05286	Y												Myb				IPR001005; SANT/Myb domain//IPR009057; H						
Supercontig_1.9:1257120-1259720	VDAG_05287														fuNOG02616	K		IPR011004; Trimeric LpxA-like//IPR024688; M	K00661	Others		-2.29	-1.31	-1.19
Supercontig_1.9:1261078-1262445	VDAG_05288																	IPR008266; Tyrosine-protein kinase, active sit				1.00	1.00	1.13
Supercontig_1.9:1264928-1265563	VDAG_05289																					-7.61	-3.48	-2.28
Supercontig_1.9:1266600-1268061	VDAG_05290																					-4.15	-1.71	-2.42
Supercontig_1.9:1270484-1272024	VDAG_05291																					-5.12	-5.58	-2.75
**L-LSR4 (110 genes)**																								
Supercontig_1.23:202659-203183	VDAG_09118																							-2.91
Supercontig_1.23:203812-207363	VDAG_09119														fuNOG03212	R	Leucine-Rich repeat protein	IPR001611; Leucine-rich repeat						
Supercontig_1.23:209816-210451	VDAG_09120																							1.31
Supercontig_1.23:212362-214296	VDAG_09121														fuNOG00899	P	Alpha-Glucoside:Hydrogen symp	IPR003663; Sugar/inositol transporter//IPR005	K08141	Transporter	[BR:ko02000]			1.17
Supercontig_1.23:214564-216889	VDAG_09122	Y	Y												fuNOG00159	S		IPR018871; GLEYA adhesin domain	K01106	Inositol pho	[PATH:ko00562][PATH:kc			
Supercontig_1.23:226071-227371	VDAG_09123														fuNOG02616	K		IPR001451; Bacterial transferase hexapeptide	K00661	Others				
Supercontig_1.23:228461-229501	VDAG_09124																							
Supercontig_1.23:231661-234819	VDAG_09125														fuNOG11553	S		IPR022198; Protein of unknown function DUF3					-3.58	-2.24
Supercontig_1.23:237277-237708	VDAG_09126																						2.35	1.78
Supercontig_1.23:238949-240294	VDAG_09127																					-1.21		
Supercontig_1.23:240964-242408	VDAG_09128														fuNOG13768	S								
Supercontig_1.23:244085-244558	VDAG_09129																							
Supercontig_1.23:247035-250143	VDAG_09130														fuNOG01097	S		IPR011043; Galactose oxidase/kelch, beta-prc				1.96	2.01	
Supercontig_1.23:251683-252108	VDAG_09131																							
Supercontig_1.23:252827-254017	VDAG_09132																							-1.60
Supercontig_1.23:254155-255300	VDAG_09133														fuNOG10077	S						1.07	1.23	1.65
Supercontig_1.23:257721-258458	VDAG_09134																							
Supercontig_1.23:259078-259470	VDAG_09135														fuNOG05714	GO		IPR018535; Domain of unknown function DUF	K01238	Others			1.32	

Scaffold:position	Gene-ID	Pathogen	Secretome	LysM	NLP	SCRPs	CAZymes	Pectin degradation	Cellulose degradation	Hemicellulose degradation	Ligin degradation	PHI_define	Kinase	Transcription factor	eggNOG_ID	eggNOG_class	eggNOG_define	IPR_define	KO_ID	KEGG_Third	BR_family/PATH_way	Responsed to cotton	Responsed to tomato	Responsed to lettuce
Supercontig_1.23:260977-262466	VDAG_09136														fuNOG00348	I	Protein involved in unsaturated fa	IPR005804; Fatty acid desaturase, type 1//IPF	K10256	Biosynthesi	[PATH:ko01040][BR:ko01			
Supercontig_1.23:262943-264629	VDAG_09137														fuNOG03696	S								
Supercontig_1.23:265052-266947	VDAG_09138																							
Supercontig_1.23:267924-268718	VDAG_09139																							
Supercontig_1.23:268853-269934	VDAG_09140														fuNOG02805	S	Transmembrane receptor	IPR017981; GPCR, family 2-like						
Supercontig_1.23:270365-270964	VDAG_09141																	IPR018289; MULE transposase domain						
Supercontig_1.23:271004-272276	VDAG_09142																							
Supercontig_1.23:272796-273367	VDAG_09143																							
Supercontig_1.23:275555-276250	VDAG_09144	Y												Zn2Cys6				IPR001138; Zn(2)-C6 fungal-type DNA-binding	K09247	Transcriptic	[BR:ko03000]			
Supercontig_1.23:277131-277585	VDAG_09145														fuNOG08398	TU	Protein involved in mRNA stabiliz	IPR006760; Endosulphine						
Supercontig_1.23:282555-284312	VDAG_09146																	IPR001023; Heat shock protein Hsp70//IPR01	K03283	Spliceosom	[PATH:ko03040][BR:ko03	1.00		
Supercontig_1.23:287456-287967	VDAG_09147														fuNOG13946	S								
Supercontig_1.23:289646-292055	VDAG_09148	Y												bZIP	fuNOG13057	S	C6 transcription factor	IPR004827; Basic-leucine zipper domain//IPR(						
Supercontig_1.23:294129-294747	VDAG_09149														fuNOG06139	O		IPR001841; Zinc finger, RING-type//IPR01308	K11982	Ubiquitin sy	[BR:ko04121]	-3.28	-1.95	-2.23
Supercontig_1.23:312142-312645	VDAG_09150														fuNOG12726	S		IPR010730; Heterokaryon incompatibility				-1.29		-1.28
Supercontig_1.23:315474-316469	VDAG_09151														fuNOG11850	Q	Short-Chain dehydrogenase	IPR002198; Short-chain dehydrogenase/reduc	K00100	Fructose ar	[PATH:ko00051][PATH:kc	3.76	3.49	1.55
Supercontig_1.23:316944-318053	VDAG_09152																	IPR021833; Protein of unknown function DUF3						
Supercontig_1.23:322029-322367	VDAG_09153																	IPR006913; Glutathione-dependent formaldehy				6.17	6.83	3.39
Supercontig_1.23:325144-326172	VDAG_09154																	IPR008030; NmrA-like//IPR016040; NAD(P)-bi	K00224	Propanoate	[PATH:ko00640][PATH:kc	4.91	5.16	2.11
Supercontig_1.23:327667-327990	VDAG_09155																					7.48	7.73	8.18
Supercontig_1.23:328834-329597	VDAG_09156																					4.36	4.84	4.61
Supercontig_1.23:330611-331059	VDAG_09157														fuNOG08349	S	BZIP transcription factor	IPR019357; Protein of unknown function DUF2					1.67	1.56
Supercontig_1.23:332314-335060	VDAG_09158																	IPR002641; Patatin/Phospholipase A2-related/	K00777	Others		-1.44	-1.45	-1.20
Supercontig_1.23:335882-339992	VDAG_09159														fuNOG03154	T	Sensor histidine kinase/response	IPR001789; Signal transduction response regu						
Supercontig_1.23:344407-345493	VDAG_09160																							
Supercontig_1.23:351337-353170	VDAG_09161	Y												HMG	fuNOG03867	R		IPR009071; High mobility group, superfamily//I	K11296	Chromoson	[BR:ko03036]	1.73	1.61	1.99
Supercontig_1.23:354854-355300	VDAG_09162																					-3.41	-2.02	-2.15
Supercontig_1.23:355323-356304	VDAG_09163																		K12584	RNA degra	[PATH:ko03018]	-1.47		
Supercontig_1.23:358001-358959	VDAG_09164	Y												C2H2 zinc finger	fuNOG04557	K	Protein involved in cation homeo	IPR007087; Zinc finger, C2H2//IPR015880; Zi						
Supercontig_1.23:360330-360819	VDAG_09165														fuNOG08398	TU	Protein involved in mRNA stabiliz	IPR006760; Endosulphine						
Supercontig_1.23:362488-362955	VDAG_09166														fuNOG06415	S						1.79	1.42	1.13
Supercontig_1.23:365504-367073	VDAG_09167														fuNOG02968	U	Protein involved in intracellular tr		K15053	Endocytosi	[PATH:ko04144]			
Supercontig_1.23:367187-367993	VDAG_09168														fuNOG01980	L	E3 RING-finger protein, member	IPR003034; DNA-binding SAP	K10627	Ubiquitin sy	[BR:ko04121][BR:ko0340(			
Supercontig_1.23:369772-370690	VDAG_09169														fuNOG00054	S	Protein involved in lagging stranc	IPR019038; DNA polymerase subunit Cdc27	K03504	Purine meta	[PATH:ko00230][PATH:kc			
Supercontig_1.23:371195-374242	VDAG_09170	Y											CMGC	Zn2Cys6	fuNOG06963	T		IPR000719; Protein kinase, catalytic domain//I	K08282	Signal trans				
Supercontig_1.23:377014-377949	VDAG_09171														fuNOG04149	I	Protein involved in protein targeti					1.25	1.49	1.74
Supercontig_1.23:380418-380675	VDAG_09172																					1.33	1.12	1.13
Supercontig_1.23:382684-383322	VDAG_09173																	IPR013078; Histidine phosphatase superfamily	K01834	Glycolysis /	[PATH:ko00010][PATH:kc		1.46	1.50
Supercontig_1.23:383350-385100	VDAG_09174														fuNOG03970	P	Chromate ion transporter protein	IPR003370; Chromate transporter	K07240	Other ion-c				
Supercontig_1.23:390242-391852	VDAG_09175																							
Supercontig_1.23:392737-394473	VDAG_09176														fuNOG03116	P	Phosphate protein	IPR001204; Phosphate transporter	K03306	Other ion-c		-1.01	-1.32	-1.18
Supercontig_1.23:396042-397680	VDAG_09177	Y												C2H2 zinc finger	fuNOG06285	K	Transcription factor that mediate	IPR007087; Zinc finger, C2H2//IPR013087; Zi				-1.12	-1.29	
Supercontig_1.23:399792-401302	VDAG_09178														fuNOG11719	E		IPR001991; Sodium:dicarboxylate symporter	K05613	Glutamater	[PATH:ko04724][PATH:kc			
Supercontig_1.23:402362-403050	VDAG_09179																							
Supercontig_1.23:404618-406716	VDAG_09180														fuNOG11852	P		IPR000175; Sodium:neurotransmitter symporte	K05037	Ion channel	[BR:ko04040]			
Supercontig_1.23:406994-408271	VDAG_09181																	IPR008030; NmrA-like//IPR016040; NAD(P)-bi				-2.04	-1.17	-2.29
Supercontig_1.23:408598-409871	VDAG_09182																					1.05	1.68	
Supercontig_1.23:410347-410842	VDAG_09183														fuNOG09060	S			K01759	Pyruvate m	[PATH:ko00620][PATH:kc		2.07	
Supercontig_1.23:411307-412361	VDAG_09184																	IPR008030; NmrA-like//IPR016040; NAD(P)-bi	K00831	Methane m	[PATH:ko00680][PATH:kc	5.01	4.88	
Supercontig_1.23:412629-413907	VDAG_09185														fuNOG04452	G	MFS multidrug transporter protei	IPR011701; Major facilitator superfamily//IPR0	K08157	Transporter	[BR:ko02000]		1.96	
Supercontig_1.23:416259-418324	VDAG_09186																	IPR021842; Protein of unknown function DUF3						
Supercontig_1.23:423557-424158	VDAG_09187	Y	Y			Y																		
Supercontig_1.23:424977-426005	VDAG_09188														fuNOG10889	Z	Protein involved in apoptosis							
Supercontig_1.23:426155-427380	VDAG_09189																	IPR002575; Aminoglycoside phosphotransfera						
Supercontig_1.23:427450-428106	VDAG_09190																					-3.13	-2.51	-2.12
Supercontig_1.23:429537-429955	VDAG_09191																					-1.09	-1.54	
Supercontig_1.23:430146-430337	VDAG_09192																					-2.60	-2.07	-1.22
Supercontig_1.23:430803-431153	VDAG_09193																							
Supercontig_1.23:443892-445250	VDAG_09194																							
Supercontig_1.23:447629-448144	VDAG_09195																							
Supercontig_1.23:448708-449366	VDAG_09196																							
Supercontig_1.23:449720-450338	VDAG_09197																							
Supercontig_1.23:451009-453001	VDAG_09198																	IPR013112; FAD-binding 8//IPR013121; Ferric						
Supercontig_1.23:453304-454368	VDAG_09199																							
Supercontig_1.23:454680-455537	VDAG_09200	Y	Y				GH128								fuNOG07224	G		IPR013781; Glycoside hydrolase, subgroup, ca						
Supercontig_1.23:456823-457116	VDAG_09201														fuNOG07506	S	Allergen Asp F7 protein	IPR009009; Barwin-related endoglucanase//IP						
Supercontig_1.23:460712-462080	VDAG_09202																							
Supercontig_1.23:463668-464498	VDAG_09203																							
Supercontig_1.23:464525-465670	VDAG_09204														fuNOG10415	S		IPR002575; Aminoglycoside phosphotransfera						
Supercontig_1.23:467562-469125	VDAG_09205																	IPR001223; Glycoside hydrolase, family 18, ca	K01183	Amino suga	[PATH:ko00520]			
Supercontig_1.23:470553-472281	VDAG_09206														fuNOG13246	S	Phospholipase, patatin family pro							
Supercontig_1.23:482730-484284	VDAG_09207																	IPR002575; Aminoglycoside phosphotransfera						
Supercontig_1.23:484295-484597	VDAG_09208																					2.57	3.06	2.96
Supercontig_1.23:491745-492158	VDAG_09209																					-1.71		
Supercontig_1.23:492698-494692	VDAG_09210														fuNOG13859	S		IPR018289; MULE transposase domain					-1.86	-1.04
Supercontig_1.23:496965-497977	VDAG_09211														fuNOG00853	D	G2/Mitotic-Specific cyclin	IPR004367; Cyclin, C-terminal//IPR006671; Cy	K02220	Cell cycle -	[PATH:ko04111]			
Supercontig_1.23:498373-500548	VDAG_09212																	IPR001128; Cytochrome P450				-1.29	-1.47	-1.13
Supercontig_1.23:500911-501883	VDAG_09213																	IPR001025; Bromo adjacent homology (BAH) (						
Supercontig_1.23:504198-506384	VDAG_09214																	IPR001623; Heat shock protein DnaJ, N-termi	K09511	Chaperones	[BR:ko03110]			
Supercontig_1.23:506477-507478	VDAG_09215	Y											AGC					IPR000719; Protein kinase, catalytic domain//I	K00924	Signal trans			1.15	
Supercontig_1.23:508320-508691	VDAG_09216																						7.30	6.63
Supercontig_1.23:509797-511030	VDAG_09217																							
Supercontig_1.23:511191-512401	VDAG_09218																							
Supercontig_1.23:513246-514238	VDAG_09219																							
Supercontig_1.23:514537-516540	VDAG_09220																	IPR013112; FAD-binding 8//IPR013121; Ferric						-4.41
Supercontig_1.23:518199-518857	VDAG_09221																							
Supercontig_1.23:519437-519952	VDAG_09222																							
Supercontig_1.23:522552-523907	VDAG_09223																							
Supercontig_1.23:526495-526707	VDAG_09224																							
Supercontig_1.23:533351-533920	VDAG_09225																							
Supercontig_1.23:536506-537773	VDAG_09226														fuNOG02860	C		IPR001107; Band 7 protein//IPR001972; Stom	K03364	Ubiquitin m	[PATH:ko04120][BR:ko04			1.13
Supercontig_1.23:538638-540607	VDAG_09227														fuNOG00180	A	Atp-Binding RNA helicase involv	IPR000629; RNA helicase, ATP-dependent, D	K14809	Ribosome E	[BR:ko03009]	3.22	2.86	2.61
