# Supplementary material for: Comparative genomics reveals cotton‐specific virulence factors in flexible genomic regions in Verticillium dahliae and evidence of horizontal gene transfer from Fusarium
Source: New Phytol. 2017 Oct 30;217(2):756–70. doi: 10.1111/nph.14861 (PMC5765495; doi:10.1111/nph.14861)
Supplement: Supplementary file 4 [file NPH-217-756-s004.pdf]

Table S20. List of genes in certain *V. dahliae* strains with best hits to protein-coding genes in *Fusarium*.

[illegible]

[illegible]	[illegible]	[illegible]
		[illegible]; Neutral/alkaline nonlysosomal ceramidase
		[illegible]; Glucose-methanol-choline oxidoreductase, N-terminal
		[illegible]; Pectin lyase fold/virulence factor/[illegible]; Pectin lyase fold
		[illegible]; Transferase/[illegible]; Chloramphenicol acetyltransferase-like domain
		[illegible]
		[illegible]; Tetratricopeptide TPR-1/[illegible]; Kinesin light chain/[illegible]; Tetratricopeptide-like helical/[illegible]; Tetratricopeptide repeat-containing domain/[illegible]; Tetratricopeptide repeat
		[illegible]; Nucleoside phosphorylase domain
		[illegible]; Tetratricopeptide TPR-1/[illegible]; Kinesin light chain/[illegible]; Tetratricopeptide-like helical/[illegible]; Tetratricopeptide repeat-containing domain/[illegible]; Tetratricopeptide repeat
34.73	4E-41	[illegible]; WD40 repeat/[illegible]; CHT nucleoside triphosphatase/[illegible]; Quinoprotein alcohol dehydrogenase-like/[illegible]; WD40/YVTN repeat-like-containing domain/[illegible]; WD40-repeat-containing domain/[illegible]; G-protein beta WD-40 repeat [illegible]
		[illegible]; Protein kinase, catalytic domain/[illegible]; Protein kinase-like domain
30.97	7E-10	[illegible]; AAA+ ATPase domain/[illegible]; ATPase, AAA-type, core/[illegible]; ATPase, AAA-type, conserved site/[illegible]; BCS1, N-terminal
		[illegible]; CHT nucleoside triphosphatase
		[illegible]; Cupin, RmlC-type/[illegible]; Cupin 2, conserved barrel/[illegible]; RmlC-like jelly roll fold
		[illegible]; NmrA-like/[illegible]; D(P)-binding domain
		[illegible]; Glucose-methanol-choline oxidoreductase, N-terminal
[illegible]	[illegible]	[illegible]; Major facilitator superfamily/[illegible]; Major facilitator superfamily domain, general substrate transporter/[illegible]; Major facilitator superfamily domain
		[illegible]; Short-chain dehydrogenase/reductase SDR/[illegible]; Glucose/ribitol dehydrogenase/[illegible]; D(P)-binding domain
31.43	5E-11	[illegible]; Alcohol dehydrogenase superfamily, zinc-type/[illegible]; GroES-like/[illegible]; Alcohol dehydrogenase GroES-like/[illegible]; D(P)-binding domain/[illegible]; Polyketide synthase, enoylreductase
		[illegible]; Short-chain dehydrogenase/reductase SDR/[illegible]; Glucose/ribitol dehydrogenase/[illegible]; D(P)-binding domain
		[illegible]; D helicase PIF1, ATP-dependent/[illegible]; Helitron helicase-like domain
		[illegible]; Homeodomain-like/[illegible]; Myb-like domain
		[illegible]; Glycoside hydrolase, family 38, core/[illegible]; Glycoside hydrolase/deacetylase, beta/alpha-barrel/[illegible]; Glycoside hydrolase, family 38, central domain
		IPR007138; Antibiotic biosynthesis monooxygenase/IPR011008; Dimeric alpha-beta barrel
		[illegible]; FAD-binding 8/[illegible]; Ferric reductase, D binding/[illegible]; Ferredoxin reductase-type FAD-binding domain
		[illegible]; Acyl transferase/acyl hydrolase/lysophospholipase
		[illegible]; Reverse transcriptase, R-dependent D polymerase
		[illegible]; Reverse transcriptase, R-dependent D polymerase
		[illegible]; Protein kinase, catalytic domain/[illegible]; Tyrosine-protein kinase, active site/[illegible]; Protein kinase-like domain
		IPR000477; Reverse transcriptase/IPR002156; Ribonuclease H domain/IPR005135; Endonuclease/exonuclease/phosphatase/IPR012337; Ribonuclease H-like domain
		IPR001138; Zn(2)-C6 fungal-type D-binding domain
59.87	0	IPR003439; ABC transporter-like/IPR003593; AAA+ ATPase domain/IPR010929; CDR ABC transporter/IPR013525; ABC-2 type transporter/IPR017871; ABC transporter, conserved site
		IPR021842; Protein of unknown function DUF3435
		IPR013103; Reverse transcriptase, R-dependent D polymerase
		IPR001878; Zinc finger, CCHC-type
		IPR000477; Reverse transcriptase/IPR005135; Endonuclease/exonuclease/phosphatase
		IPR001680; WD40 repeat/IPR015943; WD40/YVTN repeat-like-containing domain/IPR017986; WD40-repeat-containing domain
		IPR002125; CMP/dCMP deaminase, zinc-binding/IPR004794; Riboflavin biosynthesis protein RibD/IPR016193; Cytidine deaminase-like
		IPR011009; Protein kinase-like domain/IPR016477; Fructosamine/Ketosamine-3-kinase
		IPR011009; Protein kinase-like domain
		IPR024645; Mitochondrial export protein Som1
		IPR001765; Carbonic anhydrase
		IPR000254; Cellulose-binding domain, fungal/IPR004235; Scytalone dehydratase
		IPR021842; Protein of unknown function DUF3435
		IPR002151; Kinesin light chain/IPR011990; Tetratricopeptide-like helical
		IPR000845; Nucleoside phosphorylase domain
		IPR011990; Tetratricopeptide-like helical/IPR013026; Tetratricopeptide repeat-containing domain/IPR019734; Tetratricopeptide repeat
		IPR000719; Protein kinase, catalytic domain/IPR011009; Protein kinase-like domain
30.07	5E-10	IPR003593; AAA+ ATPase domain/IPR003959; ATPase, AAA-type, core/IPR003960; ATPase, AAA-type, conserved site/IPR014851; BCS1, N-terminal
36.76	1E-54	IPR000719; Protein kinase, catalytic domain/IPR001680; WD40 repeat/IPR002290; Serine/threonine- / dual-specificity protein kinase, catalytic domain/IPR008271; Serine/threonine-protein kinase, active site/IPR011009; Protein kinase-like domain/IPR015943; WD40/YVTN repeat-like-containing domain/IPR017986; WD40-repeat-containing domain/IPR019775; WD40 repeat, conserved site/IPR020472; G-protein beta WD-40 repeat/IPR020635; Tyrosine-protein kinase, catalytic domain
		IPR001878; Zinc finger, CCHC-type/IPR004875; DDE superfamily endonuclease, CENP-B-like/IPR006600; Pogo transposase / Cenp-B / PDC2, D-binding HTH domain/IPR007889; D binding HTH domain, Psq-type/IPR009057; Homeodomain-like
		IPR024222; Telomere length regulation/capping, TEN1
		IPR021833; Protein of unknown function DUF3425
38.12	1E-27	IPR007219; Transcription factor, fungi
		IPR004875; DDE superfamily endonuclease, CENP-B-like/IPR006600; Pogo transposase / Cenp-B / PDC2, D-binding HTH domain/IPR007889; D binding HTH domain, Psq-type/IPR009057; Homeodomain-like
		IPR008906; HAT dimerisation/IPR012337; Ribonuclease H-like domain
		IPR007232; Rad52/22 double-strand break repair protein
		IPR003653; Peptidase C48, SUMO/Sentrin/Ubl1
		IPR001138; Zn(2)-C6 fungal-type D-binding domain/IPR007219; Transcription factor, fungi
36.51	9E-39	
47.19	5E-17	
		IPR000719; Protein kinase, catalytic domain/IPR008266; Tyrosine-protein kinase, active site/IPR011009; Protein kinase-like domain
		IPR002575; Aminoglycoside phosphotransferase/IPR011009; Protein kinase-like domain
		IPR007087; Zinc finger, C2H2/IPR015880; Zinc finger, C2H2-like
		IPR001810; F-box domain, cyclin-like
		IPR021858; Protein of unknown function DUF3468
32.32	0.000003	IPR001623; Heat shock protein DnaJ, N-terminal/IPR007872; Zinc finger, DPH-type
38.57	3E-36	IPR001680; WD40 repeat/IPR007111; CHT nucleoside triphosphatase/IPR015943; WD40/YVTN repeat-like-containing domain/IPR017986; WD40-repeat-containing domain/IPR019775; WD40 repeat, conserved site/IPR020472; G-protein beta WD-40 repeat
		IPR001680; WD40 repeat/IPR015943; WD40/YVTN repeat-like-containing domain/IPR017986; WD40-repeat-containing domain
		IPR002110; Ankyrin repeat/IPR020683; Ankyrin repeat-containing domain
		IPR013087; Zinc finger, C2H2-type/integrase, D-binding/IPR015880; Zinc finger, C2H2-like
		IPR001878; Zinc finger, CCHC-type/IPR004875; DDE superfamily endonuclease, CENP-B-like/IPR006600; Pogo transposase / Cenp-B / PDC2, D-binding HTH domain/IPR007889; D binding HTH domain, Psq-type/IPR009057; Homeodomain-like
		IPR001878; Zinc finger, CCHC-type/IPR004875; DDE superfamily endonuclease, CENP-B-like/IPR006600; Pogo transposase / Cenp-B / PDC2, D-binding HTH domain/IPR007889; D binding HTH domain, Psq-type/IPR009057; Homeodomain-like
		IPR007213; Leucine carboxyl methyltransferase/IPR016651; Leucine carboxyl methyltransferase, LCTM1 1/IPR021121; Leucine carboxyl methyltransferase, eukaryotic
		IPR001878; Zinc finger, CCHC-type/IPR004875; DDE superfamily endonuclease, CENP-B-like/IPR006600; Pogo transposase / Cenp-B / PDC2, D-binding HTH domain/IPR007889; D binding HTH domain, Psq-type/IPR009057; Homeodomain-like
		IPR004875; DDE superfamily endonuclease, CENP-B-like/IPR006600; Pogo transposase / Cenp-B / PDC2, D-binding HTH domain/IPR007889; D binding HTH domain, Psq-type/IPR009057; Homeodomain-like
31.75	1E-41	
		IPR003000; D-dependent deacetylase, sirtuin family
33.1	8E-50	
		IPR007112; Expansin/pollen allergen, DPBB domain/IPR009009; Barwin-related endoglucanase/IPR014733; Barwin-like endoglucanase
		IPR001138; Zn(2)-C6 fungal-type D-binding domain
		IPR001138; Zn(2)-C6 fungal-type D-binding domain/IPR002409; Aflatoxin biosynthesis regulatory protein
47.23	3E-171	IPR002213; UDP-glucuronosyl/UDP-glucosyltransferase/IPR004276; Glycosyl transferase, family 28
		IPR008902; Bacterial alpha-L-rhamnosidase/IPR008928; Six-hairpin glycosidase-like
		IPR001645; Folylpolyglutamate synthetase/IPR004101; Mur ligase, C-terminal/IPR013221; Mur ligase, central
		IPR011990; Tetratricopeptide-like helical
		IPR002110; Ankyrin repeat/IPR020683; Ankyrin repeat-containing domain
		IPR007111; CHT nucleoside triphosphatase
		IPR004875; DDE superfamily endonuclease, CENP-B-like/IPR006600; Pogo transposase / Cenp-B / PDC2, D-binding HTH domain/IPR007889; D binding HTH domain, Psq-type/IPR009057; Homeodomain-like
		IPR001878; Zinc finger, CCHC-type/IPR004875; DDE superfamily endonuclease, CENP-B-like/IPR006600; Pogo transposase / Cenp-B / PDC2, D-binding HTH domain/IPR007889; D binding HTH domain, Psq-type/IPR009057; Homeodomain-like
		IPR000477; Reverse transcriptase/IPR002156; Ribonuclease H domain
		IPR022698; Protein of unknown function DUF3505
		IPR000182; GT domain/IPR016181; Acyl-CoA N-acyltransferase
		IPR000608; Ubiquitin-conjugating enzyme, E2/IPR016135; Ubiquitin-conjugating enzyme/RWD-like
		IPR001584; Integrase, catalytic core/IPR012337; Ribonuclease H-like domain/IPR013103; Reverse transcriptase, R-dependent D polymerase
		IPR002198; Short-chain dehydrogenase/reductase SDR/IPR016040; D(P)-binding domain
		IPR001878; Zinc finger, CCHC-type/IPR004875; DDE superfamily endonuclease, CENP-B-like/IPR006600; Pogo transposase / Cenp-B / PDC2, D-binding HTH domain/IPR007889; D binding HTH domain, Psq-type/IPR009057; Homeodomain-like
		IPR006677; tR intron endonuclease, catalytic domain-like/IPR011856; Endonuclease TnsA, N-terminal/resolvase Hjc/tR endonuclease, C-terminal/IPR016690; tR-splicing endonuclease, SEN34 subunit
		IPR000719; Protein kinase, catalytic domain/IPR008266; Tyrosine-protein kinase, active site/IPR011009; Protein kinase-like domain
63.64	0.000000005	IPR000254; Cellulose-binding domain, fungal/IPR002022; Pectate lyase/Amb allergen/IPR011050; Pectin lyase fold/virulence factor/IPR012334; Pectin lyase fold
		IPR001810; F-box domain, cyclin-like
77.32	0	IPR000629; R helicase, ATP-dependent, DEAD-box, conserved site/IPR001650; Helicase, C-terminal/IPR011545; D/R helicase, DEAD/DEAH box type, N-terminal/IPR014001; Helicase, superfamily 1/2, ATP-binding domain
		IPR002110; Ankyrin repeat/IPR020683; Ankyrin repeat-containing domain
		IPR003817; Phosphatidylserine decarboxylase-related/IPR005221; Phosphatidylserine decarboxylase
		IPR010285; D helicase PIF1, ATP-dependent/IPR025476; Helitron helicase-like domain
48.32	8E-74	IPR002213; UDP-glucuronosyl/UDP-glucosyltransferase/IPR004276; Glycosyl transferase, family 28
40	0.00000005	IPR001138; Zn(2)-C6 fungal-type D-binding domain
		IPR007568; RTA-like protein
		IPR010285; D helicase PIF1, ATP-dependent/IPR025476; Helitron helicase-like domain
		IPR016040; D(P)-binding domain
		IPR008030; NmrA-like/IPR016040; D(P)-binding domain
		IPR001138; Zn(2)-C6 fungal-type D-binding domain/IPR007219; Transcription factor, fungi
		IPR008030; NmrA-like/IPR016040; D(P)-binding domain
		IPR008331; Ferritin/DPS protein domain/IPR009040; Ferritin- like diiron domain/IPR009078; Ferritin/ribonucleotide reductase-like/IPR012347; Ferritin-related
		IPR021331; Protein of unknown function DUF2945
		IPR022210; Transcription activator, osmotic-stress induced
		IPR018289; MULE transposase domain
40.08	7E-110	IPR011701; Major facilitator superfamily/IPR016196; Major facilitator superfamily domain, general substrate transporter/IPR020846; Major facilitator superfamily domain
		IPR000626; Ubiquitin/IPR019955; Ubiquitin supergroup/IPR022617; Small ubiquitin-related modifier, SUMO
		IPR003830; (2R)-phospho-3-sulpholactate synthase, ComA/IPR013785; Aldolase-type TIM barrel
		IPR008397; Alginate lyase domain/IPR008929; Chondroitin AC/alginate lyase
		IPR007736; Caleosin
		IPR010730; Heterokaryon incompatibility
		IPR011009; Protein kinase-like domain/IPR018934; RIO-like kinase
		IPR001584; Integrase, catalytic core/IPR012337; Ribonuclease H-like domain
		IPR003010; Nitrilase/cyanide hydratase
		IPR002198; Short-chain dehydrogenase/reductase SDR/IPR002347; Glucose/ribitol dehydrogenase/IPR016040; D(P)-binding domain
		IPR001138; Zn(2)-C6 fungal-type D-binding domain/IPR002409; Aflatoxin biosynthesis regulatory protein
		IPR003445; Cation transporter
		IPR003445; Cation transporter/IPR004773; Potassium transport protein, high-affinity
		IPR015880; Zinc finger, C2H2-like
		IPR002528; Multi antimicrobial extrusion protein
38.71	8E-17	IPR003010; Nitrilase/cyanide hydratase/IPR018289; MULE transposase domain
		IPR023753; Pyridine nucleotide-disulphide oxidoreductase, FAD/D(P)-binding domain
		IPR002110; Ankyrin repeat/IPR020683; Ankyrin repeat-containing domain
		IPR002110; Ankyrin repeat/IPR020683; Ankyrin repeat-containing domain
		IPR000845; Nucleoside phosphorylase domain/IPR018017; Nucleoside phosphorylase
		IPR002482; Peptidoglycan-binding Lysin subgroup/IPR018392; Peptidoglycan-binding lysin domain
		IPR003593; AAA+ ATPase domain/IPR003959; ATPase, AAA-type, core/IPR014851; BCS1, N-terminal
		IPR001623; Heat shock protein DnaJ, N-terminal
		IPR011004; Trimeric LpxA-like/IPR024688; Maltose/galactoside acetyltransferase
		IPR001128; Cytochrome P450
		IPR001810; F-box domain, cyclin-like
		IPR001451; Bacterial transferase hexapeptide repeat/IPR011004; Trimeric LpxA-like
		IPR022198; Protein of unknown function DUF3723
		IPR018289; MULE transposase domain
43.48	0.0000001	IPR001138; Zn(2)-C6 fungal-type D-binding domain
		IPR001841; Zinc finger, RING-type/IPR013083; Zinc finger, RING/FYVE/PHD-type
		IPR010730; Heterokaryon incompatibility
		IPR002198; Short-chain dehydrogenase/reductase SDR/IPR002347; Glucose/ribitol dehydrogenase/IPR016040; D(P)-binding domain
		IPR021833; Protein of unknown function DUF3425
		IPR006913; Glutathione-dependent formaldehyde-activating enzyme/centromere protein V/IPR011057; Mss4-like
		IPR019357; Protein of unknown function DUF2205, coiled-coil
		IPR007087; Zinc finger, C2H2/IPR015880; Zinc finger, C2H2-like
		IPR003370; Chromate transporter
		IPR001204; Phosphate transporter
		IPR000175; Sodium:neurotransmitter symporter
		IPR008030; NmrA-like/IPR016040; D(P)-binding domain
		IPR008030; NmrA-like/IPR016040; D(P)-binding domain
30.61	7E-45	IPR011701; Major facilitator superfamily/IPR016196; Major facilitator superfamily domain, general substrate transporter/IPR020846; Major facilitator superfamily domain
		IPR002575; Aminoglycoside phosphotransferase/IPR011009; Protein kinase-like domain
		IPR001025; Bromo adjacent homology (BAH) domain
		IPR001128; Cytochrome P450
		IPR007087; Zinc finger, C2H2/IPR015880; Zinc finger, C2H2-like
		IPR002085; Alcohol dehydrogenase superfamily, zinc-type
		IPR002575; Aminoglycoside phosphotransferase/IPR011009; Protein kinase-like domain
31.13	0.000002	IPR000719; Protein kinase, catalytic domain/IPR011009; Protein kinase-like domain
		IPR000719; Protein kinase, catalytic domain/IPR011009; Protein kinase-like domain
		IPR000719; Protein kinase, catalytic domain/IPR008266; Tyrosine-protein kinase, active site/IPR011009; Protein kinase-like domain
		IPR024991; Anaphase-promoting complex subunit 11
		IPR021867; Protein of unknown function DUF3321
		IPR002575; Aminoglycoside phosphotransferase
		IPR000719; Protein kinase, catalytic domain/IPR008266; Tyrosine-protein kinase, active site/IPR011009; Protein kinase-like domain
